# An essential fifth coding ORF in the sobemoviruses^☆^

**DOI:** 10.1016/j.virol.2013.05.033

**Published:** 2013-11

**Authors:** Roger Ling, Adrienne E. Pate, John P. Carr, Andrew E. Firth

**Affiliations:** a Department of Pathology, University of Cambridge, Tennis Court Road, Cambridge CB2 1QP, UK; b Department of Plant Sciences, University of Cambridge, Downing Street, Cambridge CB2 3EA, UK

**Keywords:** Sobemovirus, Plant virus, Translation, Non-AUG initiation, Leaky scanning

## Abstract

The sobemoviruses have one of the smallest of all known RNA virus genomes. ORF1 encodes P1 which plays a role in suppression of silencing and virus movement, ORFs 2a and 2b encode the replicational polyproteins P2a and P2ab, and ORF3 encodes the coat protein. Translation of ORF2a from the genomic RNA is dependent on a leaky scanning mechanism. We report the presence of an additional ORF (ORFx), conserved in all sobemoviruses. ORFx overlaps the 5′ end of ORF2a in the +2 reading frame and also extends some distance upstream of ORF2a. ORFx lacks an AUG initiation codon and its expression is predicted to depend on low level initiation at near-cognate non-AUG codons, such as CUG, by a proportion of the ribosomes that are scanning the region between the ORF1 and ORF2a initiation codons. Mutations that disrupt translation of ORFx in turnip rosette virus prevent the establishment of infection.

## Highlights

• The plant-infecting sobemoviruses have a 4–4.5 kb genome with four know coding ORFs.
• We report an additional ORF (ORFx) that is conserved in all sobemoviruses.
• Translation of ORFx is predicted to depend on leaky scanning and non-AUG initiation.
• Mutations that disrupt translation of ORFx prevent the establishment of infection.

## Introduction

**Fig. 1:**
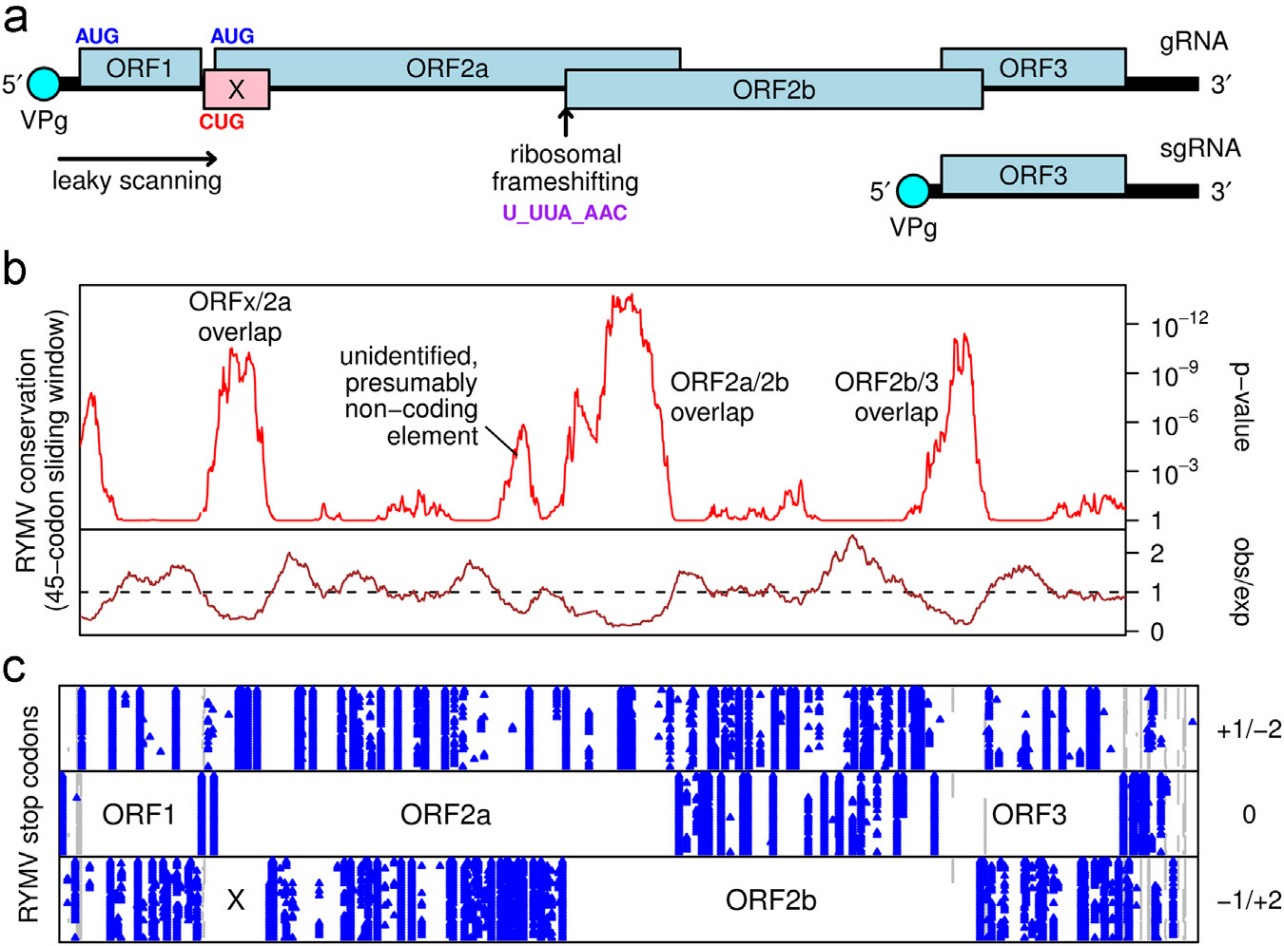
Sobemovirus genome organization. (a) Map of the ∼4.45 kb RYMV genome. ORF1 (P1), ORF2a (Pro-VPg-P10-P8 polyprotein) and ORF2b (RdRp) are translated from the genomic RNA via a combination of leaky scanning and ribosomal frameshifting. ORF3 (coat protein) is translated from a subgenomic RNA. The newly identified ORFx (X, pink) is predicted to be also translated via leaky scanning, and initiation at a near-cognate non-AUG codon, which in RYMV is a highly conserved CUG. (b) Conservation at synonymous sites in an alignment of 34 RYMV genome sequences, using a 45-codon sliding window. The lower panel show the ratio of the observed number of substitutions to the number expected under a null model of neutral evolution at synonymous sites, while the upper panel shows the corresponding *p*-value. Note the extreme reduction in synonymous substitutions in the gene overlap regions. (c) Positions of stop codons (blue) in the three possible reading frames, and alignment gaps (gray) in the 34 RYMV sequences.

Sobemoviruses (reviewed in Tamm and Truve, 2000) comprise a genus of plant viruses that is currently not assigned to any family. Species within the genus *Sobemovirus* include *Rice yellow mottle virus* (RYMV), *Cocksfoot mottle virus*, *Southern bean mosaic virus* (SBMV), *Sesbania mosaic virus*, *Lucerne transient streak virus* (LTSV), and *Turnip rosette virus* (TRoV). The genome is a single-stranded positive-sense RNA molecule that typically comprises around 4 to 4.5 kb. The 5′ end has a covalently linked viral protein (VPg), while the 3′ end is not polyadenylated (Ghosh et al., 1979). The genome contains four protein-coding ORFs (Fig. 1a). ORFs 1, 2a and 2b are translated from the genomic RNA while ORF3, which encodes the coat protein, is translated from a subgenomic RNA. ORF1 encodes P1, a protein of around 12 to 24 kDa (depending on species) that appears to play a role in systemic silencing (Voinnet et al., 1999; Sarmiento et al., 2007; Siré et al., 2008; Lacombe et al., 2010) and virus movement (Bonneau et al., 1998; Sivakumaran et al., 1998; Meier et al., 2006; Chowdhury and Savithri, 2011). The P1 amino acid sequences from different sobemovirus species are generally highly divergent (Tamm and Truve, 2000). ORFs 2a and 2b encode the replicational polyproteins, where the ORF2b product is produced as a transframe fusion with much of the ORF2a product via programmed ribosomal frameshifting around 140 to 170 codons upstream of the 3′ end of ORF2a (Mäkinen et al., 1995). The replicational polyproteins are cleaved to produce the RdRp, VPg, a serine protease, and other products called P10 and P8 (Mäkinen et al., 2000; Nair and Savithri, 2010).

Despite the presence of a 5′-linked VPg (which, in RYMV, interacts with eIF(iso)4G and may be involved in ribosome recruitment) translation is thought to be essentially 5′-end-dependent (Hacker and Sivakumaran, 1997; Sivakumaran and Hacker, 1998; Hébrard et al., 2010). In particular, translation of ORF2a depends on a leaky scanning mechanism whereby a proportion of scanning ribosomes fail to initiate on the ORF1 AUG codon (which consistently has a weak initiation context) and instead continue scanning and initiate on the ORF2a AUG codon, which (with some exceptions, see below) is normally the second AUG codon on the genomic RNA message (Sivakumaran and Hacker, 1998; Kozak, 2002).

For many years, different species of sobemoviruses were thought to have either of two startlingly different genome organizations. Some sobemoviruses were thought to have the genome organization described above (i.e. Fig. 1a) while, in other sobemoviruses, ORFs 2a and 2b were thought to be fused into a single long ORF, with the overlap region between ORFs 2a and 2b comprising a third ORF that might be accessed via ribosomal frameshifting. However, Meier and Truve (2007) demonstrated that in fact there was no such variation and all sobemoviruses conform to the genome organization displayed in Fig. 1a. Previous inconsistencies had been due to sequencing errors. Similarly, a second inconsistency – the apparent presence of two short 5′ ORFs (1a and 1b) in LTSV and TRoV – was recently shown to be a consequence of sequencing errors; in fact, LTSV and TRoV also conform to the genome organization of Fig. 1a (Sõmera and Truve, 2013).

We have been using comparative genomic methods to identify ‘hidden’ genes in RNA viruses (Firth et al., 2010, 2011a; Jagger et al., 2012; Fang et al., 2012). Here, using the same methods, we identify an additional functional ORF (ORFx) in the sobemoviruses. ORFx overlaps the 5′ end of ORF2a and extends some distance upstream. Translation of ORFx is predicted to depend on leaky scanning and non-AUG initiation. Mutations that prevent expression of ORFx prevent the establishment of infection.

## Results and discussion

### Computational analysis reveals a conserved ORF overlapping sobemovirus ORF2a

All sobemovirus sequences with coverage of ORF2a currently available in GenBank (1 Feb 2013) were identified by using TBLASTN. In total, 66 sequences were retrieved of which 34 are RYMV sequences. To begin with, the RYMV sequences were analysed separately. These 34 sequences all have full or near-full coverage of the RYMV genome. For each sequence, the coding regions were extracted and fused in frame through the artificial insertion of a C 3′-adjacent to the ribosomal frameshift site U_UUA_AAC (thus mimicking the actual sequence of the ORF2a/2b fusion when frameshifting occurs), insertion of NN after the ORF2b stop codon, and deletion of the G 5′-adjacent to the ORF2a initiation codon. This was done to facilitate a codon-based analysis. The fused coding sequences were then translated, aligned as amino acid sequences, and back-translated to produce a nucleotide sequence alignment. Next, the alignment was analyzed for conservation at synonymous sites (Fig. 1b), as described previously (Firth et al., 2011b). The analysis revealed a striking and highly statistically significant increase in synonymous site conservation in three regions: (i) the region where the 3′ end of ORF2a, shifted into the +1 reading frame by the artificial insertion of a *C* at the frameshift site, overlaps the 5′ end of ORF2b, (ii) the region where the 5′ end of ORF3 overlaps the 3′ end of ORF2b, and (iii) a region comprising ∼70 codons at the 5′ end of ORF2a. Similar regions of conservation in RYMV have been noted previously (Fargette et al., 2004). Within these three regions the mean synonymous substitution rate was reduced to (respectively) 32%, 24% and 29% of the genomic average (Fig. 1b). An inspection of the +1 and +2 reading frames relative to ORF2a revealed a conserved absence of stop codons in the +2 reading frame, from all 34 sequences, in the first 70 codons of ORF2a (Fig. 1b). This suggested that, like in regions (i) and (ii), the enhanced conservation in region (iii) might also be due to an overlapping coding sequence in, in this case, the +2 reading frame. To avoid confusion with the previously mis-annotated ORF1b of LTSV and TRoV (see above), we chose to refer to the new ORF as ORFx (Fig. 1) and to the corresponding product as Px.

**Fig. 2:**
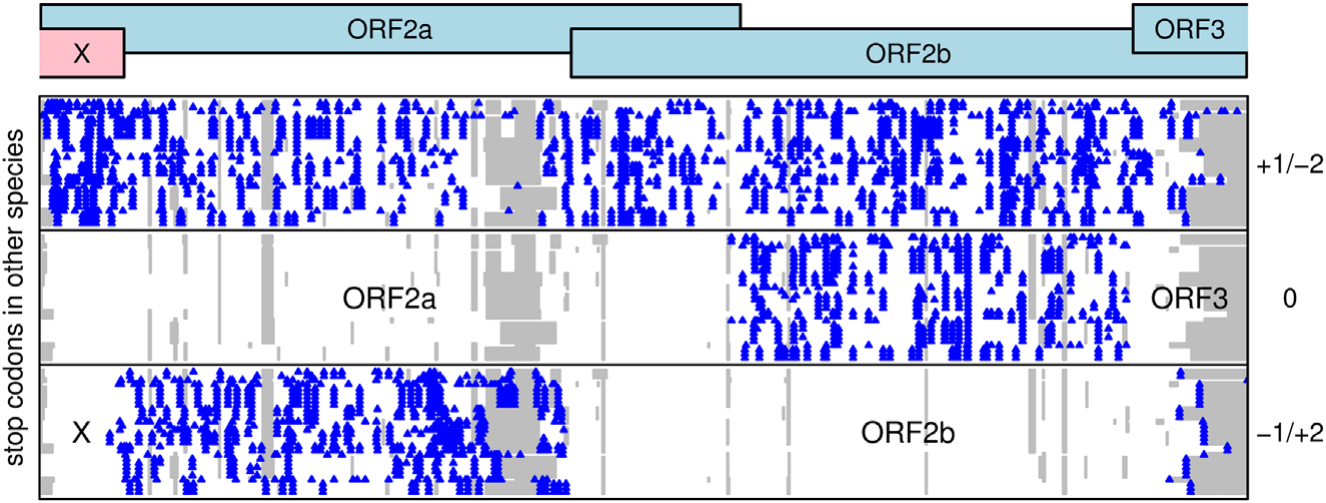
Positions of stop codons (blue) in the three possible reading frames, and alignment gaps (gray) in the ORF2ab region of 30 non-RYMV sobemovirus sequences.

In general, different species of sobemoviruses are too divergent to apply the synonymous site conservation analysis to a genus-wide alignment. However, the stop codon analysis was repeated using all available sobemovirus sequences with coverage of ORF2a (Fig. 2). Again there was a conserved absence of stop codons in the +2 reading frame for 61–88 codons (depending on species) at the 5′ end of ORF2a. Including RYMV and two new TRoV sequences reported here, a total of 64 sequences were used for this analysis (see the Methods for accession numbers). Between them, the 64 sequences represent a considerable degree of diversity (mean of 4.7 substitutions per nucleotide column over the phylogenetic tree in the ORF2a region), so even the maintenance of the ORFx open reading frame is significant evidence for its coding status (*p*<10^−5^; see the Methods).

### Translation of ORFx is predicted to depend on non-AUG initiation

As discussed previously, expression of ORF2a depends on a proportion of ribosomes scanning past the ORF1 initiation codon. In most sobemovirus sequences, the first AUG on the genomic RNA is the initiation codon for ORF1 while the second AUG is the initiation codon for ORF2a. The efficiency of initiation is determined, in part, by the context of the initiation codon. In mammals, contexts with an A at −3, or a G at −3 and a G at +4, may be regarded as ‘strong’ (Kozak, 1986). While dicotyledon plants appear to follow similar context rules, in monocotyledon plants there appears to be more preference for a G at −3 so it is possible that in monocots a G at −3 may, like an A at −3, provide a strong context even without a G at +4 (Joshi et al., 1997; Nakagawa et al., 2008). The nucleotides at −1 and −2 are also relevant but have not been fully explored in plants (Lukaszewicz et al., 2000). In sobemoviruses, the ORF1 AUG initiation codon consistently has a weak context, and a significant proportion of scanning 40S ribosomal subunits are expected to bypass it (Sivakumaran and Hacker, 1998; Kozak 2002). Initiation at non-AUG codons depends on a number of factors. Scanning ribosomes may initiate translation on any of the codons CUG, GUG, ACG, AUU, AUA, UUG and AUC, with CUG being the most efficient non-AUG initiation codon in many systems (Gordon et al., 1992; reviewed in Firth and Brierley, 2012). Initiation at such non-AUG codons normally requires a strong initiation context, but may also be enhanced in less predictable ways by RNA structure within the message (Kozak, 1990), and potentially also by the scanning 40S subunit stacking up behind another ribosome initiating at a downstream AUG codon (e.g. ORF2a) or terminating at a downstream stop codon (e.g. ORF1) if and where the spacing is appropriate (Dinesh-Kumar and Miller, 1993). Since ORFx lacks an AUG codon in all sequenced sobemoviruses, we predicted that translation of ORFx might initiate at a non-AUG codon, utilizing a proportion of the ribosomes that are scanning towards the ORF2a initiation codon.

**Fig. 3:**
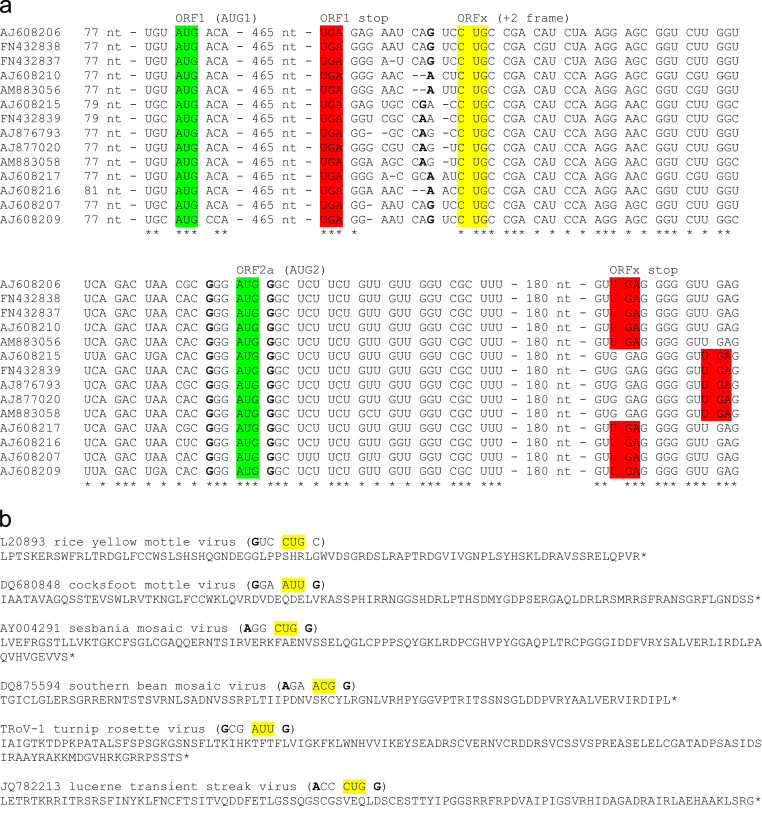
Sequence analysis of ORFx. (a) Alignment of representative RYMV sequences showing extracts of the ORF1/ORFx region. Spaces separate ORF1- and ORF2a-frame codons; ORFx is in the +2 frame relative to the indicated codons. The ORF1 and ORF2a AUG initiation codons are highlighted in green and the ORF1 and ORFx stop codons are highlighted in red. In RYMV, translation of ORFx is predicted to initiate at a highly conserved CUG codon (yellow) that coincides with the 5′ end of ORFx-frame conservation. See Supplementary data file for complete ORF1/ORFx sequences for other RYMV isolates, and for other sobemovirus species. (b) Predicted Px peptide sequences for representative sobemoviruses (see Supplementary data file for other species). Note that the ORFx initiation site is not always clear from sequence analysis, and multiple initiation sites may be utilized in some species; where there is ambiguity, one possible initiation site (context and codon indicated with bold and yellow highlight, respectively) is presented for each species (see also Supplementary data file). Further, due to this uncertainty, peptide sequences are shown with the genetic-code decoding of the predicted initiator codon; however, non-AUG initiation codons are actually expected to be decoded by initiator Met-tRNA resulting in an N-terminal methionine, rather than the indicated amino acid, for each sequence.

The large number of sequenced isolates available for RYMV makes it ideal for investigating the likely initiation site of ORFx. In the region where ORFx overlaps ORF2a, there is high conservation at the third positions of ORF2a-frame codons. Moreover, variations that do occur are often at the third positions of ORFx-frame codons (Fig. 3a and Supplementary data file). In contrast, downstream of the ORFx stop codon, the third positions of ORF2a-frame codons are rarely conserved (Supplementary data file). In RYMV, ORF1 terminates some distance upstream of ORF2a, with an intervening region (previously thought to be intergenic) of 51 to 54 nt. (Some sobemovirus species have an even longer ORF1-ORF2a ‘intergenic’ region, e.g. 93 nt in LTSV, and 141 nt in ryegrass mottle virus; while in some other species ORF1 and ORF2a overlap.) Such a lengthy region would be unusual if it were indeed intergenic as there would be no obvious reason for the virus to retain such a sequence, in particular since no subgenomic promoter, or internal ribosome entry site (IRES), or other such element is thought to be required for ORF2a translation. However the presence of a coding sequence, ORFx, explains the retention of this region. Within this region between ORF1 and ORF2a, nucleotide variability is almost exclusively confined to synonymous changes with respect to the ORFx reading frame (Fig. 3a). Nucleotide conservation starts precisely at an ORFx-frame CUG codon 10–12 nt downstream of the ORF1 stop codon. This CUG has an A or G at −3 for a strong context (in monocots) though lacks a G at +4. There are no other suitable non-AUG initiation codons in the immediate vicinity, and ORFx initiation at an upstream location (e.g. overlapping ORF1) is precluded by the presence of 0, 1 or 2 alignment gaps in different RYMV isolates just upstream of the CUG codon which would disrupt the ORFx reading frame (Fig. 3a). Thus, from sequence analysis alone, it can be predicted with some certainity that ORFx translation in RYMV initiates at this CUG codon.

All other sequenced sobemoviruses possess one or more of the standard non-AUG initiation codons, in a strong context, in the appropriate location and frame for ORFx initiation (Supplementary data file). In some species there are multiple potential initiation sites, and it is possible that several initiation sites are functionally utilized to produce different versions of ORFx with slightly different N-termini. Perhaps more likely, the combination of initiation codon identity (some are known to be significantly more efficient than others), context, and other less predictable features (e.g. RNA structure) may make one particular initiation site the favored one *in planta*. Px sequences, based on predicted initiation sites, are shown for a selection of species in Fig. 3b (see Supplementary data file for other species). Px is predicted to be typically 9.5 to 20 kDa, depending on species, with an isoelectric point usually in the range 8 to 11. Although little genus-wide conservation of amino acid motifs is apparent in Px, between species Px tends to show higher amino acid conservation than P1 (as determined by BLAST analysis of P1 and Px).

In some species (e.g. TRoV, sowbane mosaic virus, *Rubus* chlorotic mottle virus, and *Artemisia* A virus) there is an additional AUG codon upstream of the ORF1 AUG codon, initiation at which would result in the translation of a short ORF (4 to 23 codons depending on species) that terminates downstream of the ORF1 initiation codon. Reinitiation of scanning after translation of a short ORF (<30 codons) is efficient (reviewed in Jackson et al., 2012). Thus the short upstream ORF, in the species where it is present, may play a role in moving a larger proportion of ribosomes past the ORF1 initiation codon. In a few species there is an additional AUG codon between the ORF1 and ORF2a initiation codons. Again, the corresponding ORF is very short (3 to 13 codons depending on species) and many ribosomes that translate it would be expected to reinitiate scanning. These short ORFs, where present, may play a role in fine-tuning the balance of ORF1, ORFx and ORF2a expression. However, as ribosomes are known to successfully scan though the region in order to translate ORF2a and ORF2ab, the presence of these short ORFs is in no way contradictory to the model for ORFx translation.

### Clarification of the TRoV genome structure

Until recently, TRoV and LTSV alone among the sobemoviruses were annotated as having two AUG-initiated 5′ ORFs (1a and 1b). We wondered whether this unusual ORF configuration might be the result of early sequencing errors. To test this, we resequenced the strain of TRoV whose sequence had been reported previously (herein TRoV-1), and also sequenced a second strain of TRoV (herein TRoV-2).

**Fig. 4:**
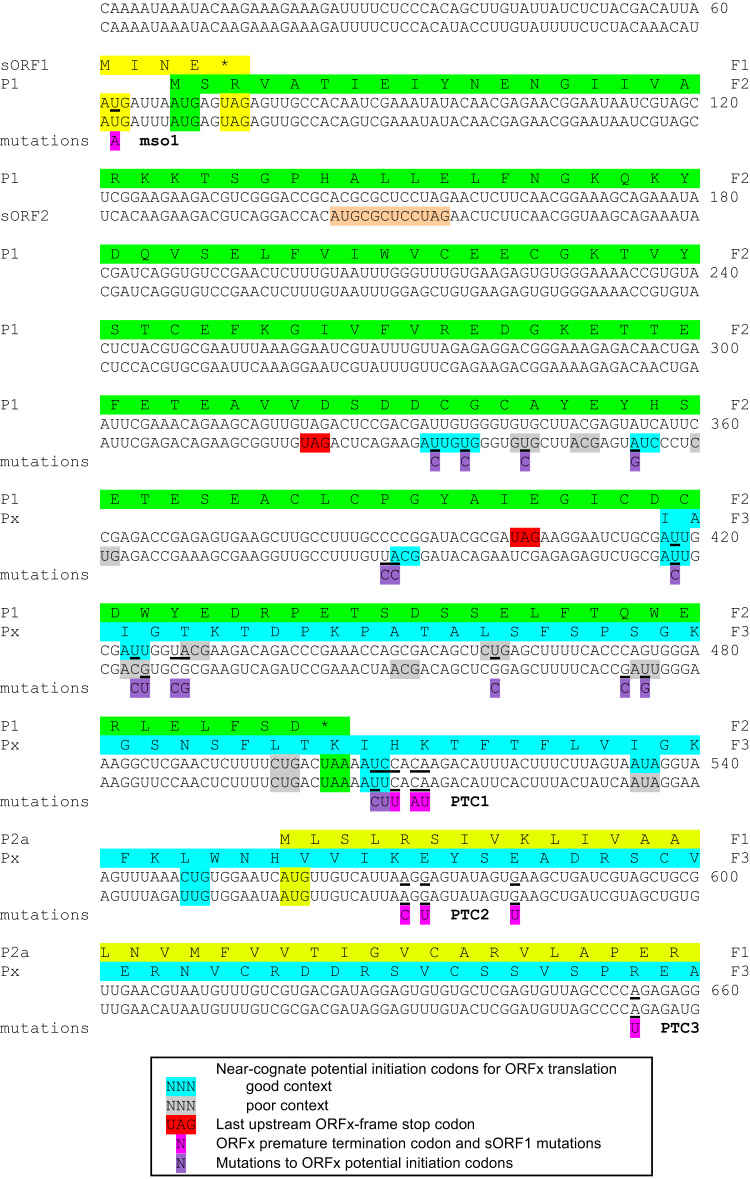
Locations of mutations made in TRoV-1 and TRoV-2. The 5′-terminal 660 nucleotides of the TRoV-1 (upper sequence) and TRoV-2 (lower sequence) genomic RNAs are shown. Numbers at right indicate genomic coordinates of the final nucleotide in each line. The sequence covers the 5′UTR, ORF1, and 5′ fragments of ORFx and ORF2a. Amino acids of predicted translation products are shown for TRoV-1 (green=P1; cyan=Px; lime green=P2a; yellow=4-codon short ORF, sORF1). F1, F2 and F3 indicate the relative reading frames. Initiation and termination codons are highlighted with the same color as the corresponding peptide sequences. The 3-codon short ORF, sORF2, present only in TRoV-2 is highlighted in orange. Near-cognate non-AUG potential initiation codons for ORFx translation are highlighted in cyan (good context: either A at -3, or G at both -3 and at +4, for dicots) or gray (poor context). The last upstream ORFx-frame stop codon (that delimits the furthest possible 5′-extent of ORFx) is highlighted in red. Mutations made in TRoV-1 and TRoV-2 to introduce premature termination codons (PTCs) into ORFx (mutants PTC1, PTC2 and PTC3) are shown in pink and the mutated nucleotides are underlined in each sequence. The mutation made in TRoV-1 to prevent translation of the 4-codon ORF that overlaps the ORF1 initiation codon (mutant mso1) is similarly indicated. Mutations made to ORFx potential initiation codons are shown in purple and the mutated nucleotides are underlined in each sequence (see also Fig. 7).

Viral cDNAs were obtained as three overlapping RT-PCR products using primers based on the published sequence AY177608, besides the data obtained by sequencing some of our PCR products. These products were sequenced to obtain the sequences of TRoV-1 and TRoV-2. In addition to various nucleotide differences, two single nucleotide deletions (G539 and U693 in AY177608) were observed relative to the published sequence. The net effect of these deletions was to fuse ORF1b in-frame with ORF2a so that it becomes the 5′ end of ORF2a. At the same time, a new ORF, without an in-frame AUG codon, appears in the correct position and frame for ORFx (Fig. 4; Supplementary data file).The nucleotide differences also introduce a small number of amino acid changes into P1 (1 change), P2a (1 change), P2b (1 change) and P3 (3 changes). TRoV-2 had the same overall arrangement of features but with greater sequence divergence (90% mean nucleotide identity to TRoV-1). 5′ and 3′ RACE were used to identify the sequences at the ends of the viral RNAs. The 5′ sequence obtained was the same as that previously reported for TRoV (5′-CAAAAU; Callaway et al., 2004), and similar to 5′ sequences reported for many other sobemoviruses (e.g. CAAAAU in *Artemisia* virus A; ACAAAAU in subterranean clover mottle and velvet tobacco mottle viruses; and CACAAAAU in SBMV, though here the 5′ C is not always present and may be erroneous; Hacker and Sivakumaran, 1997; Olspert et al., 2011). On the other hand, an extension of 51 nucleotides was observed at the 3′ end relative to the previously published TRoV sequence. The TRoV-1 and TRoV-2 sequences have been submitted to GenBank with accession numbers KC778720 and KC778721, respectively.

Recently, corrected sequences for both TRoV and LTSV were independently published by Sõmera and Truve (2013) (accession numbers JQ782212 and JQ782213). In both cases, the previously annotated AUG-initiated ORF1b was found to be an artifact of sequencing errors, and the predicted non-AUG initiated ORFx becomes apparent in the corrected sequences. Thus, the genome arrangement ORF1, ORFx, ORF2a, ORF2b, ORF3, with non-AUG initiation for ORFx, appears to be a universal feature of the genus *Sobemovirus*.

### Generation of TRoV infectious clones

In order to test the functional importance of ORFx, we generated TRoV-1 and TRoV-2 infectious clones. PCR fragments were assembled into a modified pUC vector with a T7 promoter added to the primer at the 5′ end and an *Msc*I site at the 3′ end. The 5′ end would be expected to have an additional two G nucleotides added when transcribed by T7 RNA polymerase, whereas the correct 3′ end could be generated by linearization with *Msc*I prior to transcription. 5′ RACE on RNA extracted from plants infected using the capped transcripts revealed that the additional G nucleotides were lost during replication *in planta*. For agroinfection, additional constructs were made by subcloning into the binary vector pBI121. The correct 5′ end would be generated from the 35S promoter in the vector, but additional vector sequences would be added at the 3′ end. 3′ RACE of RNA extracted from agroinfected plants revealed that this additional 3′ sequence was lost during viral replication, while the additional 51 nucleotides of sequence we identified relative to the previously reported TRoV sequence was retained. Inoculation of *Arabidopsis thaliana* with in vitro transcribed RNA resulted in symptomatic infection for both strains. To our knowledge, these are the first infectious clones of an *Arabidopsis*-infecting sobemovirus.

### ORFx knockout mutants fail to establish systemic infection in planta

Although the precise initiation site of ORFx can be bioinformatically predicted for RYMV (see above), this was not possible for TRoV due to the limited number of sequenced isolates (i.e. two) and the presence of multiple near-cognate non-AUG codons in the region where initiation of ORFx translation is expected to occur (see below). Thus, we chose to knock out expression of the wild type (WT) ORFx product by inserting premature termination codons (PTCs) into the ORFx reading frame. Several PTC mutants were tested to guard against inadvertently mutating functional non-coding RNA elements (although none are known in this region). The mutations used to introduce three sets of PTCs (PTC1, PTC2 and PTC3) in each strain are shown in Fig. 4. All of the mutations are synonymous with respect to other viral proteins (specifically P2a). PTC1 and PTC2 each involve three nucleotide mutations and result in the introduction of two stop codons into ORFx, while PTC3 involves a single nucleotide mutation and introduces a single stop codon. (The former guard against reversion while the latter allows one to test for reversion pressure, although in our experiments PTC3 was not observed to revert as evidenced by failure to establish infection.)

In TRoV, as in some other sobemoviruses, additional AUG-initiated short ORFs are present upstream of ORFx. Both TRoV-1 and TRoV-2 have a 4-codon AUG-initiated ORF (weak context) that overlaps the ORF1 initiation codon (Fig. 4). Since ribosomes may efficiently reinitiate scanning after translation of a very short ORF (Jackson et al., 2012), the presence of this ORF may serve to move a greater proportion of ribosomes past the ORF1 AUG codon. TRoV-2, but not TRoV-1, also contains a 3-codon AUG-initiated ORF (weak context) in an internal region of ORF1 (Fig. 4). To investigate the role of the 4-codon ORF, we generated an additional TRoV-1 mutant (mso1; mutated short-ORF-1) in which the AUG was mutated to AAG (Fig. 4).

For each strain, a negative control mutant (pol-mut) was made in which the GDD polymerase motif in P2b was altered to ADA. This was expected to knock out RNA-dependent RNA polymerase activity and hence viral replication.

**Fig. 5:**
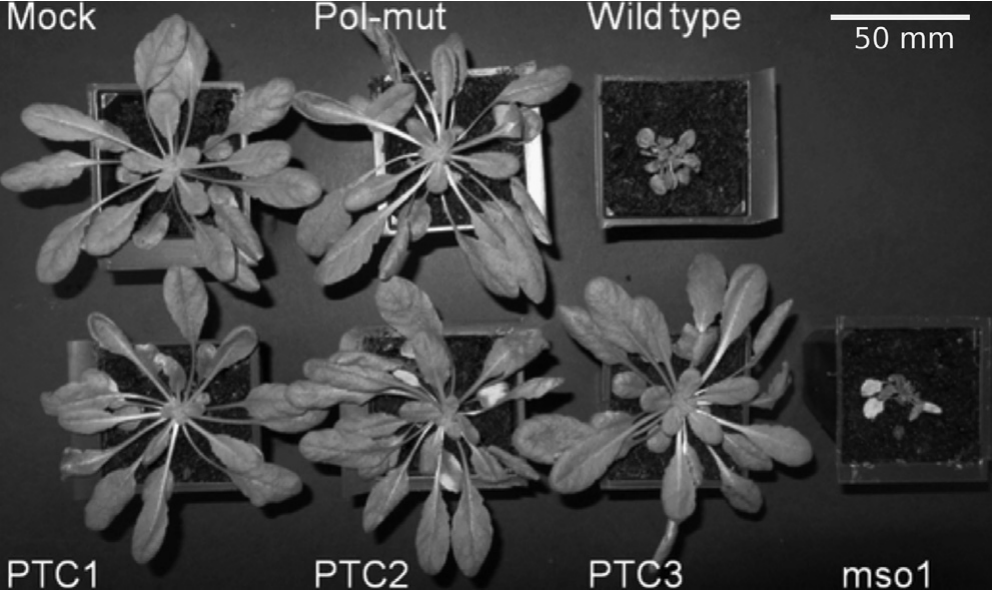
Typical symptoms of *A. thaliana* inoculated with TRoV or TRoV mutants. Plants were agroinoculated with *Agrobacterium tumefaciens* GV3101 containing a binary vector expressing WT or mutant TRoV-1, or mock infected, as indicated. Each plant shown was chosen at random from three replicates, but all replicates appeared similar. Similar results were obtained using TRoV-2 mutants, or by inoculation with capped in vitro transcripts by rubbing Carborundum-dusted leaves (see Table 1).

**Fig. 6:**
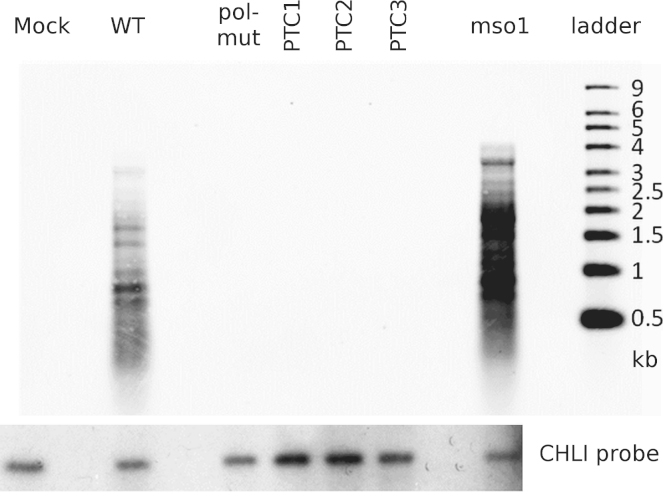
Northern blot analysis of RNA extracted from *A. thaliana* inoculated with wild type (WT) TRoV or TRoV mutants. RNA extracted from agroinoculated plants was denatured with glyoxal and separated on a 1% agarose gel followed by blotting and probing with biotinylated probes specific for a region of the coat protein gene of TRoV (TRoV-1 in this case), and digoxigenin-labelled probe specific for the host magnesium chelatase (CHLI) gene. A small amount of both types of probe specific for the Ambion millennium markers was included as well to aid aligning the images. Detection of biotin-labelled probes was via streptavidin labelled with an infrared dye detected in the 800 nm channel of a LiCor imager. Chemiluminescent detection of digoxigenin was via alkaline phosphatase-labelled anti-DIG FAb fragments and CDP-star substrate using X-ray film.

*A. thaliana* plants were inoculated either mechanically with in vitro synthesized capped transcripts, or by agroinoculation. A summary of the results obtained by inoculation with WT and mutants of the two strains of TRoV is given in Table 1. Symptoms were only observed consistently with WT constructs and the mso1 mutant. Symptomatic plants showed stunted growth, frequently with twisted petioles (Fig. 5). Inoculation with the negative control pol-mut, or with any of the three ORFx PTC mutants, did not result in the plants showing phenotypes distinguishable from mock infected plants. In addition, RNA extracted from pooled plants inoculated with WT or mso1 TRoV consistently tested positive for viral RNA by RT-PCR (20 cycles, data not shown) or northern analysis (Fig. 6), whereas RNA extracted at the same time from pooled plants inoculated with the other mutants tested negative for viral RNA by these methods. The reason for the large number of bands observed in the northern blots for infected samples is not clear, but was not obviously related to RNA quality, with all samples showing intact ribosomal RNAs on stained gels (data not shown). Similar bands were observed when plants inoculated with RNA transcripts were analyzed, so the extra RNA species do not appear to be an artifact of agroinoculation. Full-length genome of about 4 kb appeared to represent a very small proportion of the total virus-specific RNA in TRoV-infected *Arabidopsis* and no single subgenomic RNA was identified by northern blot or 5′ RACE. Nonetheless, the bands observed appear to serve as a marker for RNA replication.

### Analysis of ORFx translation in vitro

Given that there are a number of near-cognate non-AUG potential initiation sites for ORFx translation in TRoV (Fig. 4), and the paucity of TRoV sequence data precludes bioinformatic prediction of the functionally utilized site(s), we decided to investigate TRoV ORFx translation in wheat germ extract.

**Fig. 7:**
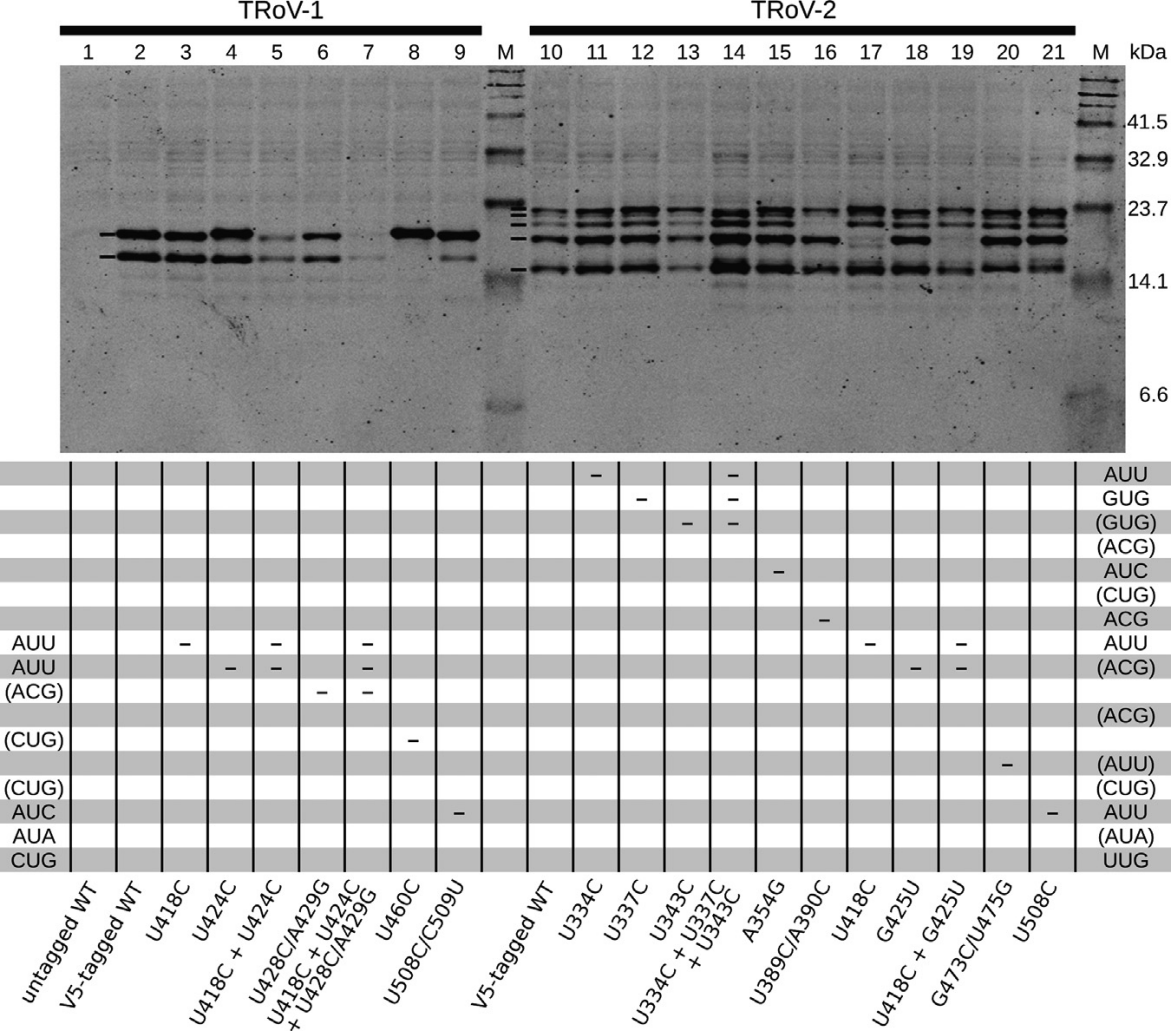
Analysis of potential ORFx initiation sites in TRoV. Transcripts from full-length TRoV clones containing V5-tagged ORFx with the wild type (WT) ORFx initiation codon(s), or with selected potential ORFx initiation codons mutated, were translated in wheat germ extract, and subjected to western analysis using an anti-V5 mAb. The short lines to the left of the bands indicate the major V5-tagged products—two for TRoV-1 and five for TRoV-2. The apparent molecular masses of prestained markers calibrated against unstained markers for the gel system are indicated at right. The mutations made in each construct are indicated at bottom. Near-cognate non-AUG potential initiation codons in TRoV-1 (left) and TRoV-2 (right) are listed in order below the gel. Codons without parentheses correspond to codons highlighted in cyan (good context) in Fig. 4, and codons in parentheses correspond to codons highlighted in gray (poor context) in Fig. 4. Codons mutated in each construct are indicated with ‘-’s.

Translation of full-length transcripts with a sequence encoding a V5 tag appended to the 3′ end of ORFx to produce V5-tagged Px allowed Px-related proteins to be detected with anti-V5 antibody by western analysis. A variety of bands were observed, suggesting that multiple non-AUG codons were utilized in this situation. The largest products were larger for TRoV-2 than for TRoV-1, consistent with the presence of additional upstream potential non-AUG initiation sites in TRoV-2 (Fig. 4). The predicted molecular masses of the largest V5-tagged ORFx products are 15.4 kDa (TRoV-1) and 18.6 kDa (TRoV-2). Low molecular mass proteins frequently show aberrant migration and, in this case, the ORFx products migrate more slowly than expected relative to the marker scale. Potential initiation codons were mutated by site-directed mutagenesis and WT and mutant in vitro transcripts were translated in wheat germ extract. Mutations are shown in Fig. 4 and the corresponding translations are shown in Fig. 7. Only two major products were observed for WT TRoV-1 (lane 2) whereas five were observed for WT TRoV-2 (lane 10), the second largest in TRoV-2 being less well-expressed than the others.

In TRoV-1, a U460C mutation (CUG→CCG) resulted in the loss of the lower band (lane 8). This codon is not conserved in TRoV-2. CUG is normally the strongest of the non-AUG initiation codons, and this may compensate for the relatively poor context (for dicots) of this codon (G at −3 but no G at +4). The effects of a triple mutant knocking out three closely spaced potential initiation codons (AUU, AUU and ACG in the region 417–431; lane 7) compared to single and double mutants targeting these codons (lanes 3–6) suggested that multiple initiation sites contributed to the upper band.

The five major bands observed for TRoV-2 are designated bands 1, 2, 3, 4 and 5 (from top to bottom). A triple mutant U334C+U337C+U343C (AUU→ACU+GUG→GCG+GUG→GCG) resulted in the loss of band 1, and increased usage of the initiation site(s) producing band 2 (lane 14). Individually, none of the three mutations noticeably altered the expression pattern (lanes 11, 12 and 13), suggesting that band 1 corresponds to proteins translated from more than one of these potential initiation sites and/or that mutagenesis of one initiation site results in utilization of another site. The initiation codon or codons corresponding to band 2 are not known, but there is an untested CUG codon at 360–362 that, perhaps together with the tested AUC at 354–356 (lane 15), may account for band 2. Band 3 was essentially eliminated by U389C/A390C mutations (U ACG→C CCG; lane 16). Band 4 was eliminated by a U418C mutation (AUU→ACU; lane 17). Therefore translation of this product initiates solely at the AUU at 417–419 (in contrast to the similarly sized product in TRoV-1). None of the tested mutations eliminated band 5. However potential initiation sites downstream of the site of the PTC1 mutation were not tested since the PTC1 mutation prevented systemic infection so that translation of at least one essential ORFx product was expected to initiate upstream of this site.

In several cases, bands were observed to migrate slightly differently in different lanes (e.g. the TRoV-1 upper band appeared to migrate more slowly in lanes 4 and 8). This could be a result of downstream mutations affecting the amino acid sequence (e.g. the U460C mutation in lane 8 replaces CUG leucine with CCG proline; prolines tend to reduce the migration rate of proteins). In other cases, mutation of one initiation site may increase usage of a near-by initiation site. A similar effect has been observed in turnip yellow mosaic tymovirus, where two closely spaced AUG codons direct translation of two overlapping ORFs: when the second AUG is mutated, translation from the first AUG codon increases, in contrast to the normal rules of leaky scanning. This effect may be observed for inter-AUG spacer lengths of up to around 10 nt (Matsuda and Dreher, 2006).

The large number of products observed was interesting but may simply be a reflection of the overall low efficiency of non-AUG initiation (in contrast, a single AUG codon in a strong context can capture the vast majority of scanning ribsomes, leading to a single major product). Nonetheless, it should be noted that in vitro systems are not ideal for investigating the efficiency of leaky scanning and non-AUG or context-dependent initiation. The stringency of initiation codon selection can be affected by a number of factors, including ionic conditions (Salerno-Rife et al., 1980) and the relative abundances of initiation factors such as eIF1 and eIF5 (Ivanov et al., 2010; Loughran et al., 2012), which may differ between the wheat germ system and virus-infected plants. Moreover the transcripts translated in wheat germ extract lack the 5′-linked VPg protein that is present in *bona fide* viral transcripts. Thus these results are exploratory rather than definitive. Unfortunately the V5-tagged ORFx virus constructs were found to be non-infectious, so we were unable to use them to study ORFx expression *in planta*.

## Conclusions

We have provided initial evidence for a conserved and essential new gene, ORFx, in the sobemoviruses. Translation of ORFx is predicted to depend on non-AUG initiation. A variety of plant viruses are known to utilize non-AUG initiation, generally as part of a leaky scanning mechanism by which multiple proteins are expressed from a single mRNA (reviewed in Firth and Brierley, 2012). In several cases, a combination of non-AUG and poor-context AUG initiation codons allows production of three or even four functional proteins from a single transcript (Fütterer et al., 1996; Turina et al., 2000; Castaño et al., 2009). In *Pelargonium* line pattern virus (family *Tombusviridae*), for example, the two carmovirus-like movement proteins (p7 and p9.7) and the coat protein are all translated from a single subgenomic RNA, with p7 utilizing an AUG in poor context, p9.7 utilizing a GUG initiation codon, and the coat protein utilizing the second AUG on the message (Castaño et al., 2009). This is similar to the ORF1/ORFx/ORF2a configuration proposed here for sobemoviruses.

Research into the in vitro translation products of sobemovirus virion RNA and/or in vitro-transcribed RNA dates back to the early 1980s (e.g. Salerno-Rife et al., 1980; Rutgers et al., 1980; Morris-Krsinich and Hull, 1981; Mang et al., 1982; Morris-Krsinich and Forster, 1983; Mäkinen et al., 1995; Tamm et al., 1999). It may seem surprising therefore that the ORFx product or products have not previously been reported. In some cases, additional faint low molecular mass products have in fact been observed but remained unexplained (e.g. Salerno-Rife et al., 1980). In general, however, the requirement for leaky scanning and the low initiation efficiency of non-AUG codons, the potential multiple initiation sites for ORFx in some species (leading to band smearing), and the small size and therefore poor labelling and potential aberrant migration of the ORFx product, probably all contribute to making Px a very difficult protein to detect in vitro or *in planta*.

Clearly these results would be enhanced by a direct detection of Px in virus-infected cells. However, despite trying a number of approaches, we have not to date been successful in this endeavour. A polyclonal antibody to the peptide CAKKEDGAHRKGRRP (‘C’+amino acids 5–18 from the C-terminal end of TRoV-2 Px) was obtained from GenScript, but proved ineffective. Indeed the antibody was only able to detect relatively large amounts of the 14-aa peptide, or bacterially expressed ORFx protein (∼50–100 ng) on Western blots, and the expression level of ORFx *in planta* may simply be below the detection limit. It is possible that ORFx may be detectable with different antibody preparations, perhaps utilizing a different sobemovirus species with a different Px sequence. In addition, two attempts were made to produce Px-tagged virus. To do this, a sequence encoding either Strep Tag II or a V5 tag was fused to the 3′ end of ORFx and the 5′ end of ORF2a, disrupted by this insertion, was duplicated downstream to generate an intact ORF2a with initiation codon downstream of the tag sequence (see the Methods). Since ORF2a initiation depends on leaky scanning, the potential for which was predicted to remain essentially unaltered, it was thought that these tagged viruses might be infectious. However, both failed to establish infection (potentially due to the C-terminal tag interfering with Px function).

The presence of a novel virus gene may be demonstrated in a number of ways. The evolutionary signature assessed via comparative genomics summarizes the results of nature's countless experiments over the course of evolution. Given the high phylogenetic divergence between different sobemovirus species, the presence of a conserved ORF cannot easily be otherwise explained. Knockout mutant phenotypes also provide direct evidence of function. Although they do not necessarily distinguish between a functional ORF and a functional non-coding element, in this case several different mutants, each with just a few carefully chosen point substitutions, all supported the ORFx hypothesis. Moreover, a non-coding RNA element would not explain the triplet periodicity of nucleotide conservation in the ORFx reading-frame upstream of the ORF2a initiation codon in RYMV (Fig. 3a). While it would be preferable to also obtain a direct detection of Px in infected plants, this is not in itself the strongest evidence for a functional gene. Even non-functional polypeptides may be translated at some level (‘translational noise’; Yewdell and Hickman, 2007), while some functional proteins are only produced at extremely low levels (e.g. the influenza A virus PA-X frameshift product; Jagger et al., 2012). Due to its potential confounding role in mutational analyses of P1 to define P1 function, besides the role of ORFx itself in virus infection, our present findings are of significant relevance to the field. The precise role of Px in virus infection will be investigated in future work.

## Methods

### Computational analysis

Sobemovirus nucleotide sequences in GenBank with full coverage of ORF2a were identified using TBLASTN (Altschul et al., 1990). The following sequences were retrieved: AB040446, AB040447, AF055887, AF055888, AF208001, AJ608206, AJ608207, AJ608208, AJ608209, AJ608210, AJ608211, AJ608212, AJ608213, AJ608214, AJ608215, AJ608216, AJ608217, AJ608218, AJ608219, AJ876793, AJ877020, AM883054, AM883055, AM883056, AM883057, AM883058, AM940437, AM990928, AY004291, AY177608, AY376451, AY376452, AY376453, AY376454, DQ481606, DQ680848, DQ875594, EF091714, FJ669143, FN432837, FN432838, FN432839, FN432840, FN432841, GQ845002, HM163159, HM754263, HQ680399, JF495127, JN620802, JQ782213, JX123318, JX961551, JX966244, JX966245, JX966246, JX966247, JX966248, L20893, L34672, L40905, M23021, U23142, U31286, Z36903 and Z48630. Most have complete or near-complete coverage of the virus genome, but partial sequence Z36903 was removed as it has 100% nucleotide identity to a subsequence of Z48630. Several early sobemovirus sequences have significant sequencing errors, in particular with regards to the frameshift configuration of ORFs 2a and 2b (Meier and Truve, 2007). The following sequences have ORFs 2a and 2b incorrectly fused into a single long ORF: AB040446, AF055887, AF055888, L20893, L34672, U23142, U31286 and M23021. For the computational analyses, AF055887, AF055888 and M23021 were ‘corrected’ by swapping in the resequenced regions DQ481603 and DQ481604 from Meier and Truve (2007). L20893, U23142 and AB040446 were manually ‘corrected’ via careful amino acid and nucleotide alignment to closely related sequences. L34672 (one of several available SBMV sequences) was removed as it could not be reliably corrected. U31286 (LTSV) contains insertion/deletion errors both in the ORF2a/ORF2b overlap region and in the ORF1/ORFx region so was removed in favor of the recent LTSV sequence, JQ782213, which has 97% nucleotide identity to U31286 (Sõmera and Truve, 2013). AY177608 (TRoV) also has insertion/deletion errors in the ORF1/ORFx region so was removed in favor of the two new TRoV sequences reported here. The 806-nt partial TRoV sequence, JQ782212, reported by Sõmera and Truve (2013) is 99% identical to our TRoV-1 sequence and was not used.

Sequences were extracted, translated, aligned and back-translated to produce nucleotide sequence alignments using EMBOSS and ClustalW (Rice et al., 2000; Larkin et al., 2007). Synonymous site conservation was calculated as described previously (Firth et al., 2011b). The statistical significance of the conserved presence of a +2 frame ORF of at least 61 codons overlapping the 5′ end of ORF2a in highly divergent sobemovirus sequences was evaluated by randomly shuffling (*n*=100,000) ORF2a-frame codon columns within the ORFx overlap region of the sobemovirus genus-wide ORF2a alignment, and calculating what fraction of shufflings preserve an open reading frame in the +2 frame. Since the position of the ORFx stop codon varies between species, the region shuffled corresponded to the region of the alignment that is free of stop codons in all sequences (i.e. the shortest ORFx/ORF2a overlap length). This procedure controls for any bias for or against random long +2 frame ORFs due to ORF2a-frame local amino acid usage, codon usage, or nucleotide biases, and importantly also controls for phylogenetic non-independence of the aligned sequences. The inter-species divergences of the P1 and Px amino acid sequences were compared by applying TBLASTN to the sixteen representative Px peptide sequences shown in Fig. 3b and the Supplementary data file, and the P1 peptide sequences from the same isolates.

### Isolation of RNA from plant material

The initial isolation of RNA from dried leaves derived from TRoV-infected *Brassica nigra* (isolated in Kimmeridge, Dorset, UK) (kindly supplied by Dr Hui Wang, Centre for Ecology and Hydrology, Wallingford, Oxfordshire, UK; herein TRoV-2) or from an isolate originally described in Broadbent and Heathcote (1958) (kindly supplied from the Roger Hull collection by Samantha Bennett and Adrian Fox, Food and Environment Research Agency, York, UK; herein TRoV-1) was carried out by phenol:chloroform extraction of frozen leaves followed by precipitation with 2 M lithium chloride. The RNA was ethanol precipitated twice and resuspended in 50 μl water for use in cDNA synthesis. Isolation of RNA from plants inoculated with synthethic viral RNA or subjected to agroinoculation was carried out with Trizol (Life Technologies Ltd) extraction followed by lithium chloride precipitation (Harvey et al., 2011).

### RT-PCR and sequencing of TRoV cDNA

Three overlapping fragments of the TRoV genome were generated for each strain using primers based on the published sequence, AY177608. A T7 promoter sequence and restriction site were added to the primer at the 5′ end of the virus sequence to facilitate cloning and generation of infectious transcripts. Viral cDNA was synthesized using SuperScript III reverse transcriptase (Life Technologies) according to the manufacturer's instructions, using both primers subsequently used for the first or only round of PCR. First round PCRs were carried out with Elongase (Life Technologies) and second round PCRs with Pfu (Promega), with denaturation carried out at 94 °C for 15 s, annealing at 55 °C for 15 s, and extension at 68 °C and 1 min/kb (Elongase) or 72 °C and 2 min/kb (Pfu) for 40 cycles. Initial denaturation was 30 s for Elongase and 2 min for Pfu whilst the final extensions were 7 min at the appropriate temperature. The 5′ fragments were generated with T7TRoV and TRoV1497L for TRoV-1 and the same primers followed by the nested primers T7TRoV2 and TRoVconF1L for TRoV-2. The central fragment was generated using primers TRoVconF2U and TRoVconF2L for TRoV-1 and primers TRoV665U and TRoV3135L2 followed by primers TRoVconF2U and TRoVconF2L2 for TRoV-2. The 3′ fragments were generated with TRoV2475U and TRoVend2 for both strains. The PCR products were gel purified using the Wizard SV gel and PCR clean up system (Promega) and sequenced using the primers indicated in Table S1.

### 5′ and 3′ RACE

The primers used are shown in Table S2. To identify the 5′ end of the viral RNA, primer TRoV374L2 or TRoV374L3 for TRoV-1 and TRoV-2 respectively were used for cDNA synthesis on RNA extracted from dried leaves as described above. The cDNA was diluted to 50 μl and purified by phenol/chloroform extraction, back extraction of the phenolic phase with 30 μl 10 mM Tris HCl pH 8, and passage through a CHROMA spin 200 column (Clontech) as described by the manufacturer to remove nucleotides and primers. The cDNA was divided into two, one part being used for tailing with deoxy-GTP and the other with deoxy-ATP using terminal deoxynucleotidyl transferase (TdT, New England Biolabs) according to the manufacturer's instructions. The tailed cDNA was purified as described above and used in nested PCR reactions with the primer used in the cDNA synthesis plus TRT5 or T3E for (dG or dT tailed cDNA respectively) followed by TRoV241L plus C-outer or T-outer for the corresponding second round PCRs. PCRs were carried out with Elongase for the first round (conditions as above for a 1 kb product) and PHUSION polymerase (Finnzymes) using the HF buffer with an initial denaturation at 98 °C followed by 40 cycles of 10 s at 98 °C and 15 s at 72 °C, and a final extension of 7 min at 72 °C for the second round.

Attempts to identify the 5′ end of the negative strand using a similar method as used for the positive strand were not successful, although cloned products gave potential sequence beyond that present in the GenBank sequence for TRoV-1, so 3′ RACE was performed following polyadenylation of total RNA from the dried leaves. RNA (0.4–2 μg) was polyadenylated using polyA polymerase and freshly made buffer followed by phenol extraction and ethanol precipitation. The cDNA was synthesized using the T3E primer and SuperScript III. Following incubation at 50 °C for 1 h and heat inactivation of the enzyme at 70 °C for 15 min, the cDNA was treated with ribonuclease H for 30 min at 37 °C and one quarter was used in the first round PCR with TRoV3405U and T3E using Elongase. The second round PCR was carried out using PHUSION polymerase and primers TRoV3940U and T-outer. Sequencing of the gel-purified product was carried out using the TRoV3940U primer.

### Assembly of full-length TRoV clones

Attempts to amplify the whole genome in one piece were not successful and the overlaps of the three fragments lacked suitable restriction sites so sequential overlap extension PCR was used to assemble the complete sequence. TRoV-1 5′ and central fragments were assembled with T7TRoV2 and TRoVconF2L whilst for TRoV-2 T7TRoV2 and TRoV3135L2 were used. New fragments had to be amplified for the 3′ end of the genome as the original ones used a primer based on the incomplete GenBank sequence. The TRoV-1 3′ end was amplified with primers TRoVconF3U and TRoVend4 whilst that of TRoV-2 was amplified with primers TRoVconF3Ub and TRoVend4. The gel-purified products were assembled with the previously assembled 5′ and central fragments with primers T7TRoV2 and TRoVend4. The assembled genomes were digested with *Mlu*I and *Msc*I and ligated into a pUC vector modified to contain these sites. Several clones were sequenced in their entirety and one clone of each strain was modified by in vitro mutagenesis to match the consensus sequence derived from the original sequencing of PCR products.

### Mutagenesis of TRoV clones

The primers used to introduce the PTC1, PTC2, PTC3 and pol-mut mutations into the virus genomes are listed in Table S3. The altered nucleotides are underlined. Mutagenesis was carried out using the QuikChange procedure (Agilent). The mutant constructs were checked by sequencing. The entire sequence of the clones in pBI121 was obtained from three PCR fragments similar to those used in the original cloning, amplified from DNA extracted from the *Agrobacterium* culture used to produce the glycerol stocks. In these pBI121 clones, one additional change in P2a was found in the pol-mut construct of TRoV-1 (nt 1702, D to Y amino acid change) but this would not affect the use of this clone as it was the negative control and not expected to produce infectious virus.

To add carboxy-terminal tags to ORFx, we first generated full-length virus clones in which the overlap region between ORFx and ORF2a was duplicated, AUG codons in any reading frame in the first copy of the duplicated region were removed via mutations that were synonymous with respect to ORFx, and the 3′ copy of the duplicated region was mutated (synonymously with respect to ORF2a) to prevent homologous recombination. These multiple changes, along with sequence encoding a carboxy-terminal linker following ORFx (GSGSGT) and Strep Tag II, were introduced into the TRoV-1 and TRoV-2 clones using synthetic DNA fragments. In addition, for construction of the V5-tagged clones, stop codons were introduced upstream of ORF2a – as occurs in the WT sequences – since test translations in wheat germ extract with the previously constructed Strep Tag II tagged clones revealed that additional, larger versions (presumably non-AUG-initiated N-terminal extensions) of the P2a and P2ab proteins were synthesized.

### Insertion of full-length TRoV constructs into the pBI121 vector

Overlap extension PCR was used to position the 5′ end of the TRoV genome adjacent to the 35S promoter of the binary vector pBI121 (Clontech). The primers and enzymes used differed for constructs from the two strains owing to differences in unique restriction sites between them. The primers used are given in Table S4. The underlined sequences are derived from TRoV and the remaining sequence from the vector. Primer TR35SL was used with 35SU (TRoV-1) or pBI1U (TRoV-2) to generate a fragment containing part of the 35S promoter from the vector and the start of TRoV. A second overlapping fragment was generated with primers TR35SU and TRoV3Xend (TRoV-1) or TRoV1Xend (TRoV-2). These fragments were gel purified and joined by overlap extension PCR with 35SU and TRoV3Xend followed by digestion with *Bsa*I (TRoV-1), or with pBI1U and TRoV1Xend followed by digestion with *Sbf*I and *Age*I (TRoV-2). These fragments were gel purified and ligated with gel-purified fragments containing the remainder of the TRoV sequence (*Bsa*I to *Msc*I for TRoV-1 and *Age*I to *Msc*I for TRoV-2) and appropriately digested pBI121 (*Bsa*I and *Eco*53KI for TRoV-1 and *Sbf*I and *Eco*53KI for TRoV-2). Note that these constructs should produce the correct 5′ end upon transcription from the 35S promoter but the 3′ end is blunt-end ligated upstream of the termination/polyadenylation signal of the vector and would initially therefore have additional vector sequences and a poly(A) tail.

### Infection of *A. thaliana* with recombinant TRoV

TRoV was previously shown to infect *A. thaliana* (Callaway et al., 2004). Two methods were employed to inoculate *A. thaliana* ecotype Col-0 plants (germinated for 2 weeks followed by 2 weeks growth of the seedlings with a short photoperiod, 8 h/day). Capped RNA transcribed in vitro with T7 RNA polymerase from plasmids linearized with *Msc*I was rubbed onto Carborundum-dusted leaves with the frosted end of a glass slide (approximately 5 μg at 700–900 ng/μl distributed over 3 or 4 lower leaves). RNA was purified by digestion with RNase free DNase, phenol/chloroform extraction, passage through a 1 ml spin column containing Sephacryl HR500, and ethanol precipitation. Alternatively, leaves were agroinoculated using *Agrobacterium tumefaciens* GV101 containing viral sequences cloned into pBI121. Bacterial cells were suspended in 0.2 mM acetosyringone before infiltration. Mock inoculations were carried out with water or 0.2 mM acetosyringone.

### In vitro translation of TRoV RNA

Full-length transcripts of TRoV prepared as described above were translated in wheat germ extract (Promega) according to the manufacturer's instructions using 90 mM potassium acetate and 1×10^11^ copies of RNA in 5 μl reactions containing both the amino acid mix without methionine and that without leucine. Reactions were terminated by addition of an equal volume of 100 μg/μl ribonuclease A and incubation at 37 °C for 20 min. Three microlitre samples were heated with an excess of SDS PAGE sample buffer (Laemmli, 1970) and run on Tris Tricine gels (Schägger and von Jagow, 1987) using 2.6% and 5% bis-acrylamide in the stock solutions in place of 3% and 6%, and 16.5% resolving gels containing 6 M urea. Western transfer was carried out using 0.3 M Tris, 0.1 M acetic acid pH 8.6, for 1 h at 25 V with a current limit of 500 mA. Blocking was carried out for 1 h with 5% low fat skimmed milk in phosphate buffered saline (PBS) and the membrane was incubated overnight at 4 °C with anti-V5 mAb (Invitrogen) diluted in the same buffer. Following five washes with PBS containing 0.1% Tween-20, the blots were incubated for 1 h in PBS containing 0.1% Tween-20 and 1/10,000 goat anti-mouse conjugated with IRdye800 (LiCOR). The blots were washed as before and rinsed in PBS before scanning with an Odyssey infrared scanner (Licor).

### Northern analysis

Total RNA (1 μg except Millenium [Ambion] markers for which 1 μl of a 1 in 10 dilution was used) were treated with an equal volume of Glyoxal sample load dye (Ambion) for 30 min at 50 °C prior to loading on a 1% agarose gel in 10 mM sodium phosphate buffer and running with buffer recirculation. Gels were blotted onto neutral Nylon membrane (Hybond N, GE Healthcare) overnight by capillary action with 20X SSC. Prehybridization was carried out for 5 h at 68 °C with 50% formamide, 5X SSPE, 0.5% SDS, 100 μg/ml each of denatured salmon sperm DNA, and Torula RNA. Probes were made by in vitro transcription with the Maxiscript T7/T3 kit (Ambion) using linearized plasmid templates to generate minus sense RNA of a fragment of the 3′ end of the appropriate TRoV strain or the *A. thaliana* magnesium chelatase gene. The labelled nucleotide was either 16-biotin-UTP (TRoV probes) or 11-digoxigenin UTP (magnesium chelatase) and, following DNase treatment, probes were diluted to 50 μl and nucleotides removed with NucAway Spin columns (Ambion). One fifth of TRoV and magnesium chelatase probes along with one tenth and one twentieth of probes specific for the Millenium markers labelled with biotin or digoxigenin respectively were added to fresh hybridization buffer and incubated with the blots overnight at 68 °C. Blots were rinsed twice and incubated twice for 15 min with 0.5X SSC 0.1% SDS at 68 °C. Detection was essentially as described for digoxigenin labelled probes (Roche) except 2X blocking solution was used, a 1/10,000 dilution of streptavidin-AlexaFluor680 was added to the antibody incubation step, and the blocking and antibody incubation steps were extended to 1 h. All incubations from the addition of the antibodies were carried out in the dark and the process interrupted prior to addition of the DIG detection buffer to scan the blot with the Odyssey infrared scanner at 700 nm. Detection of DIG labelled probes was carried out with CDP-Star (Roche).

### RT-PCR

Reverse transcription was carried out on RNA extracted from inoculated plants using SuperScript III (Invitrogen) at 50 °C according to the manufacturer's instructions using primers TRoV2475U and TRoVend2 or SulfU (CACACAACTAGTGCGTCTCTTCTTGGAACATC) and SulfL (GCTGAAAGTCGACGCGAACTTCTCGGAAACTAGA). Two microlitres was used in a PCR with the correponding primers using 20 cycles for the TRoV primers and 30 cycles for the magnesium chelatase primers. One tenth of each reaction was run on a 1% agarose gel in TAE buffer with 0.5 μg/ml ethidium bromide.

## Figures and Tables

**Table 1:** Summary of data obtained from inoculation of *A. thaliana* with wild type and mutant TRoV.

Inoculum^a^	RNA inoculation			Agroinoculation		
	Symptoms^b^	PCR^b^	Northern^b^	Symptoms^b^	PCR^b^	Northern^b^

Mock	0/1,0/5	−	n.d.,−	0/3,0/1,0/3	−,−,n.d.	−,−,n.d.
TRoV-2 WT	2/2,1/3	+,+	n.d.,+	4/4,16/18	+,n.d.	+,n.d.
TRoV-2 pol-mut	0/2,0/3	−,−	n.d.,−	0/2	−	−
TRoV-2 PTC1	0/2,0/3	−,−	n.d.	0/3	−	−
TRoV-2 PTC2	0/2,0/3	−,−	n.d.	0/2,1/18^c^	−,n.d.	−,n.d.
TRoV-2 PTC3	n.d.,0/3	n.d.,−	n.d.	0/3	−	−
TRoV-1 WT	2/2,2/3	+,+	n.d.,+	3/3,13/13,3/3	+,n.d.,n.d.	+,n.d.,+
TRoV-1 pol-mut	0/2,0/3	−,−	n.d.,−	0/3,0/10	−,n.d.	−,−
TRoV-1 PTC1	0/2,0/3	−,−	n.d.	0/3,0/3	−,n.d.	−,−
TRoV-1 PTC2	0/2,0/3	−,−	n.d.	0/3,1/12^c^,0/3	−,n.d.,n.d.	−,n.d.,−
TRoV-1 PTC3	0/2,0/3	−,−	n.d.	0/3,0/3	−,n.d.	−,−
TRoV-1 mso1	3/3	+	+	3/3,3/3	+,n.d.	+,−

a WT=wild type, PTC=premature termination codon mutant.

b Commas separate individual experiments; n.d.=not determined.

c ‘Symptoms’ were very small plants that may reflect damage during inoculation rather than actual infection.

## Footnotes

Appendix A Supplementary data associated with this article can be found in the online version at doi:10.1016/j.virol.2013.05.033.
