# Supplementary material for: An essential fifth coding ORF in the sobemoviruses
Source: Virology. 2013 Nov;446(1-2):397–408. doi: 10.1016/j.virol.2013.05.033 (PMC3791421; doi:10.1016/j.virol.2013.05.033)
Supplement: Supplementary file 1 — Supplementary data [file mmc1.pdf]

Alignments of the 5' region of the genome for available sobemovirus sequences

Alignments cover the 5'UTR, ORF1, ORFx and the 5' end of ORF2a. Alignments are separated into triplets, with gaps inserted at the 5' end and around the junction between the end of ORF1 and the start of ORF2a to ensure that ORF1-frame and ORF2a-frame codons are separated by spaces (except where this is impossible due to ORF1 overlapping with ORF2a in a different reading frame).

Note that, where relevant, the “Last upstream ORFx-frame stop” codon is also annotated as this delimits the maximal 5' extent of ORFx. I.e. ORFx translation could potentially initiate at any near-cognate non-AUG codon, in good initiation context, 3' of this point although for some species it is not clear which of several possible non-AUG codons is likely to be used most efficiently *in planta*.

```
AUG         ORF1 and ORF2a start codons.
CUG etc     Likely ORFx start codon.
AUU etc     Selected other potential ORFx start codons, where present.
UAG etc     Stop codons for ORF1 and ORFx.
UAG etc     Other stop codons of note (e.g. the last upstream ORFx-frame stop codon).
AUG...UAG Short ORFs, where present.
```

Note that an A at -3 (regardless of the nucleotide at +4), or a G at -3 together with a G at +4, may normally be regarded as comprising a strong initiation context. In monocots there appears to be similar preference for A or G at -3 in cellular genes which suggests that, for monocots, a G at -3 may provide a similarly strong context as an A at -3. Other aspects of the local sequence composition and propensity for RNA structure may modulate initiation efficiency in less predictable ways.

Rice yellow mottle virus

```
                 5'UTR
AJ608206        -AC AAU UGA AGC UAG GAA AGG AGC AUA UUG CGA AAG CGA UCC CUC CUU CCG ACG AAC AAU
L20893          -AC AAU UGA AGC UAG GAA AGG AGC AUA UUG CGA AAG C-A UCC CUC CUU CCG ACG AAC AAU
FN432838        -UC AAU UGA AGC UAG GAA AGG AGC AUA UUG CGU AAG CGA UCC CUC CUU CCG ACG AAC AAU
FN432837        -AC AAU UGA AGC UAG GAA AGG AGC AUA UUG CGA AAG CGA UCC CUC CUU CCG ACG AAC AAU
U23142          -AC AAU UGA AGC UAG GAA AGG AGC AUA UUG CGA AAG CGA UCC CUC CUU CCG ACG AAC AAU
AJ608212        -AC AAU UGA AGC UAG GAA AGG AGC AUA UUG CGA AAG CGA UCC CUC CUU CCG ACG AAC AAU
FN432841        -AC AAU UGA AGC UAG GAA AGG AGC AUA UUG CGA AAG CGA UCC CUC CUU CCG ACG AAC AAU
AJ608213        -AC AAU UGA AGC UAG GAA AGG AGC AUA UUG CGA AAG CGA UCC CUC CUU CCG ACG AAC AAU
AJ608210        -AC AAU UGA AGC UAG GAA AGG AGC AUA UUG CGA AAG CGA UCC CUC CUU CCG ACG AAC AAU
AM883054        -AC AAU UGA AGC UAG GAA AGG AGC AUA UUG CGA AAG CGA UCC CUC CUU CCG ACG AAC AAU
AJ608211        -AC AAU UGA AGC UAG GAA AGG AGC AUA UUG CGA AAG CGA UCC CUC CUU CCG ACG AAC AAU
AM883055        -AC AAU UGA AGC UAG GAA AGG AGC AUA UUG CGA AAG CGA UCC CUC CUU CCG ACG AAC AAU
AM883056        -AC AAU UGA AGC UAG GAA AGG AGC AUA UUG CGA AAG CGA UCC CUC CUU CCG ACG AAC AAU
AJ608215        -AC AAU UGA AGC UAG GAA AGG AGC AUA UUG CGU AAG CGA UCC CUC CUU CCG CCG AAC AAU
AM883057        -AC AAU UGA AGC UAG GAA AGG AGC AUA UUG CGA AAG CGA UCC CUC CUU CCG CCG AAC AAU
FN432839        -AC AAU UGA AGC UAG GAA AGG AGC AUA UUG CGA AAG CGA UCC CUC CUU CCG CCG AAC AAU
AJ876793        -AC AAU UGA AGC UAG GAA AGG AGC AUA UUG CGA AAG CGA UCC CUC CUU CCG CCG AAC AAU
AJ877020        -AC AAU UGA AGC UAG GAA AGG AGC AUA UUG CGA AAG CGA UCC CUC CUU CCG CCG AAC AAU
AM883058        -AC AAU UGA AGC UAG GAA AGG AGC AUA UUG CGA AAG CGA UCC CUC CUU CCG CCG AAC AAU
AJ608217        -AC AAU UGA AGC UAG GAA AGG AGC AUA UUG CGA AAG CGA UCC CUC CUU CCG ACG AAC AAU
AJ608218        -AC AAU UGA AGC UAG GAA AGG AGC AUA UUG CGA AAG CGA UCC CUC CUU CCG ACG AAC AAU
AJ608216        -AC AAU UGA AGC UAG GAA AGG AGC AUA UUG CGC AAG CGA UCC CUC CUU CCG ACG AAC AAU
AJ608207        -AC AAU UGA AGC UAG GAA AGG AGC AUA UUG CGA AAG CGA UCC CUC CUU CCG CCG AAC AAU
FN432840        -AC AAU UAA AGC UAG GAA AGG AGC AUA UUG CGA GAG CGA UCC CUC CUU CCG CCG AAC AAU
AJ608219        -AC AAU UGA AGC UAG GAA AGG AGC AUA UUG CGA AAG CGA UCC CUC CUU CCG CCG AAG AAU
AJ608214        -AC AAU UGA AGC UAG GAA AGG AGC AUA UUG CGA AAG CGA UCC CUC CUU CCG CCG AAC AAU
AJ608208        -AC AAU UGA AGC UAG GAA AGG AGC AUA UUG CGA AAG CGA UCC CUC CUU CCG ACG AAC AAU
AJ608209        -AC AAU UGA AGC UAG GAA AGG AGC AUA UUG CGA AAG CGA UCC CUC CUU CCG CCG AAC AAU
                  * *** * * *** *** *** *** *** *** *** **   ** * * *** *** *** ***  ** **  ***

                                                    ORF1 (AUG1; poor context)
AJ608206        UGU AGC CGC A-- CUG C-- AUC G-- UGU AUG ACA CGG UUG GAA GUU CUU AUA CGA CCG ACU
L20893          UGU AGC CAC A-- CUG C-- AUC G-- UGU AUG ACA CGG UUG GAA GUU CUU AUA CGA CCG ACU
FN432838        UGU AGC CAC U-- CUG C-- CUC G-- UGU AUG ACA CGG UUG GAA GUU CUU AUA CGA CCG ACU
FN432837        UGU AGC CAC G-- CUG C-- AUC G-- UGU AUG ACA CGG UUG GAA GUU CUU AUA CGA CCG ACU
U23142          UGC AGC CAC G-- CUG C-- AUC G-- UGU AUG ACA CGG UUG GAA GUU CUU AUA CGA CCG ACU
AJ608212        UGU AGC CAC G-- CUG C-- AUC G-- UGU AUG ACA CGG UUG GAA GUU CUU AUA CGA CCG ACU
FN432841        UGU AGC CAC G-- CUG C-- AUC G-- UGU AUG ACA CGG UUG GAA GUU CUU AUA CGA CCG ACU
AJ608213        UGU AGC CAC G-- CUG C-- AUC G-- UGU AUG ACA CGG UUG GAA GUU CUU AUA CGA CCG ACU
AJ608210        UGU AUC CAC G-- CUG C-- UAC G-- UGU AUG ACA CGG UUG GAA GUU CUU AUA CGA CCG ACU
AM883054        UGU AUC CAC G-- CUG C-- UAC G-- UGU AUG ACA CGG UUG GAA GUU CUU AUA CGA CCG ACU
AJ608211        UGU AUC CAC G-- CUG C-- UAC G-- UGU AUG ACA CGG UUG GAA GUU CUU AUA CGA CCG ACU
AM883055        UGU AUC CAC G-- CUG C-- UAC G-- UGU AUG ACA CGG UUG GAA GUU CUU AUA CGA CCG ACU
AM883056        UGU AUC CAC G-- CUG C-- UAC G-- UGU AUG ACA CGG UUG GAA GUU CUU AUA CGA CCG ACU
```

```
AJ608215        UGU AGC CAC U-- CUG CUC AUC G-- UGC AUG ACA CGG UUG GAA GUU CUU AUA CGA CCG ACA
AM883057        UGU AGC CGC G-- CUG CUC GUC G-- UGC AUG ACA CGG UUG GAA GUU CUU AUA CGA CCG ACA
FN432839        UGU AGC CGC G-- CUG CUC GUC G-- UGC AUG ACA CGG UUG GAA GUU CUU AUA CGA CCG ACA
AJ876793        UGU AGC CAC A-- CUG C-- AUC G-- UGU AUG ACA CGG UUG GAA GUU CUU AUA CGA CCG ACU
AJ877020        UGU AGC CAC G-- CUG C-- AUC G-- UGU AUG ACA CGG UUG GAA GUU CUU AUA CGA CCG ACA
AM883058        UGU AGC CAC A-- CUG C-- AUC G-- UGU AUG ACA CGG UUG GAA GUU CUU AUA CGA CCG ACU
AJ608217        UGU AGC CGC U-- CUG C-- CUC G-- UGU AUG ACA CGG UUG GAA GUU CUU AUA CGA CCG ACU
AJ608218        UGU AGC CGC U-- CUG C-- CUC G-- UGC AUG ACA CGG UUG GAA GUU CUU AUA CGA CCG ACU
AJ608216        UGU AGC CGC UGA CUG C-- CUC GUG UGU AUG ACA CGG UUG GAA GUU CUU AUA CGA CCG ACU
AJ608207        UGU AGC CAC G-- CUG C-- AUC G-- UGC AUG ACA CGG UUG GAA GUU CUU AUA CGA CCG ACU
FN432840        UGU AGC CAC G-- CUG C-- AUC G-- UGC AUG ACA CGG UUG GAA GUU CUU AUA CGA CCG ACU
AJ608219        UGU AGC CAC G-- CUG C-- AAC G-- UGU AUG ACA CGG UUG GAA GUU CUU AUA CGA CCG ACU
AJ608214        UGU AGC CAC UUU CUG C-- GUC G-- UGC AUG CCA CGG UUG GAA GUU CUU AUA CGA CCG ACU
AJ608208        UGU AGC CAC G-- CUG C-- AUC G-- UGU AUG ACA CGG UUG GAA GUU CUU AUA CGA CCG ACU
AJ608209        UGU AGC CAC G-- CUG C-- GUC G-- UGC AUG CCA CGG UUG GAA GUU CUU AUA CGA CCG ACU
                **  * * * *     *** *     * *   **  ***  ** *** *** *** *** *** *** *** *** **

AJ608206        CAG CAG ACU GUG GCA AAA GCC AUC GCC GUG GGC UAU ACG CAC GCA CUC ACC UGG GUU UGG
L20893          UCG CAG ACU GUG GCA AAA GCC AUC GCC GUG GGC UAU ACG CAC GCA CUC ACC UGG GUU UGG
FN432838        CAG CAG ACU GUG GCA AAA GCC AUC GCC GCG GGC UAU ACG CAC GCA CUC ACC UGG GUU UGG
FN432837        CAG CAG ACU GUG GCA AAA GCC AUC GCC GCG GGC UAC ACG CAC GCA CUC ACC UGG GUU UGG
U23142          CAG CAG ACU GUG GCA AAA GCC AUC GCC GCG GGC UAU ACG CAC UCG CUC ACC UGG GUU UGG
AJ608212        CAG CAG ACU GUG GCA AAA GCC AUC GCC GCG GGC UAU ACG CAC GCA CUC ACC UGG GUU UGG
FN432841        CAG CAG ACU GUG GCA AAA GCC AUC GCC GCG GGC UAU ACG CAC GCA CUC ACC UGG GUU UGG
AJ608213        CAG CAG ACU GUG GCA AAA GCC AUC GCC GCG GGC UAU ACG CAC GCA CUC ACC UGG GUU UGG
AJ608210        UUG CAG ACC GUG GAA AAG GCC AUU GCC GCG GGC UAU ACG CAC ACG CUC ACC UGG AUU UGG
AM883054        UUG CAG ACC GUG GAA AAG GCC AUU GCC GCG GGC UAU ACG CAC ACG CUC ACC UGG AUU UGG
AJ608211        UUG CAG ACC GUG GAA AAG GCC AUU GCC GCG GGC UAU ACG CAC ACG CUC ACC UGG AUU UGG
AM883055        UUG CAG ACC GUG GAA AAG GCC AUU GCC GCG GGC UAU ACG CAC ACG CUC ACC UGG AUU UGG
AM883056        UUG CAG ACC GUG GAA AAG GCC AUU GCC GCG GGC UAU ACG CAC ACG CUC ACC UGG AUU UGG
AJ608215        GAG CAG ACU GUG GCA AAA GCC AUC GCC GUG GGC UAU ACG CAC ACA CUC ACC UGG GUU UGG
AM883057        GAG CAG ACU GCG GCA AAA GCC AUC GCC GUG GGC UAU ACG CAC GCA CUC ACC UGG GUU UGG
FN432839        GAG CAG ACC GUG GCA AAA GCC AUC GCC GUG GGC UAU ACG CAC GCA CUC ACC UGG GUU UGG
AJ876793        GAG CAG ACU GUG GCA AAA GCC AAC GCC GUG GGC UAU ACG CAC GCA CUC ACC UGG GUU UGG
AJ877020        GAG CAG ACU GUG GCA AAA GCC AAC GCC GUG GGC UAU ACG CAC ACA CUC ACC UGG GUU UGG
AM883058        GAG CAG ACU GUG GCA AAA GCC AAC GCC GUG GGC UAU ACG CAC GCA CUC ACC UGG GUU UGG
AJ608217        GAG CAG ACU GCG GCG AAA GCC AUU GCC GUG GGC UAU ACG CAC ACA CUC ACC UGG GUU UGG
AJ608218        GAG CAG ACU GCG GCG AAA GCC AUU GCC GUG GGC UAU ACA CAC GCA CUC ACC UGG GUU UGG
AJ608216        GAG CAG ACA GCG GCA AAA GCC AAC GCC GUG GGC UAU ACG CAC GCA CUC ACC UGG GUU UGG
AJ608207        CAG CAG ACU GUG GCA AAA GCC AUU ACC GCG GGC UAU ACG CAC GCA CUC ACC UGG GUU UGG
FN432840        CAG CAG ACU GUG GCA AAA GCC AUU ACC GCG GGC UAU ACG CAC GCA CUC ACC UGG GUU UGG
AJ608219        CAG CAG ACU GUG GCA AGA GCC AUU ACC GCG GGC UAU ACG CAC GCA CUC ACC UGG GUU UGG
AJ608214        CAG CAG ACU GUG GCA AAA GCC AUU ACC GCG GGC UAU ACG CAC GCA CUC ACC UGG GUU UGG
AJ608208        CAG CAG ACU GUG GCA AAA GCC AUC GCC GCG GGC UAU ACG CAC GCA CUC ACC UGG GUU UGG
AJ608209        CAG CAG ACU GUG GCA AAA GCC AUC ACC GCG GGC UAC ACG CAC GCA CUC ACC UGG GUU UGG
                  * *** **  * * *   *   *** *    ** * * *** **  **  ***  *  *** *** ***  ** ***

AJ608206        CAU UCU CAG ACC UGG GAC GUU GAC GCA GUG AGC GAU CCA GUU CUC AGC GCC GAC UUC AAC
L20893          CAU UCU CAG ACC UGG GAC GUU GAC GCA GUG AAC GAU CCA GUU CUC AGC GCC GAC UUC AAC
FN432838        CAU UCU CAG ACC UGG GAC GUU GAC GCA GUG AAC GAU CCA GUU CUC AGC GCC GAC UUC AAC
FN432837        CAU UCU CAG ACC UGG GAC GUC GAC GCU GUG AAC GAU CCG GUU CUU AGC GCC GAU UUC AGC
U23142          CAU UCU CAG ACC UGG GAC GUU GAC GCA GUG AAC GAU CCA GUU CUC AGC GCC GAC UUC AAC
AJ608212        CAU UCU CAG ACC UGG GAC GUU GAC GCU GUG AAC GAU CCA GUU CUC AGU GCU GAC UUC AAC
FN432841        CAU UCU CAG ACC UGG GAC GUU GAC GCU GUG AAC GAU CCA GUU CUC AGU GCU GAC UUC AAC
AJ608213        CAU UCU CAG ACC UGG GAC GUU GAC GCU GUG AAC GAU CCA GUU CUC AGU GCU GAC UUC AAC
AJ608210        CAU CCU CAG AUC UGG GAC GUU AAC GGU GUG AGU GAU CCC ACU CUU GCC GCU GAC UUC AAC
AM883054        CAU CCU CAG AUC UGG GAC GUU AAC GGU GUG AGU GAU CCC ACU CUU GCC GCU GAC UUC AAC
AJ608211        CAU UCU CAG AUC UGG GAC GUU AAC GGU GUG AGU GAU CCC ACU CUU GCC GCU GAC UUC AAC
AM883055        CAU UCU CAG AUC UGG GAC GUU AAC GGU GUG AGU GAU CCC ACU CUU GCC GCU GAC UUC AAC
AM883056        CAU UCU CAG AUC UGG GAC GUU AAC GGU GUG AGU GAU CCC ACU CUU GCC GCU GAC UUC AAC
AJ608215        UAU CCG CAG ACC UGG GAC GUU GAU UCU GUG AAC GAU CCA GUU CUC AGA GCC GAC UUC GAC
AM883057        UAU CCG CAG ACC UGG GAC GUU GAU UCU GUG AAC GAU CCA GUU CUC AGA GCC GAC UUC AAC
FN432839        UAU CCG CAG ACC UGG GAC GUU GAU UCU GUG AAC GAU CCA GUU CUC AGA GCC GAC UUC AAC
AJ876793        CAU UCU CAG ACC UGG GAC GUU GAU UCU GUG AAC GAU CCA GUU CUC AGA GCC GAC UUC AAC
AJ877020        CAU UCU CAG ACC UGG GAC GUU GAC UCU GUG AAC GAU CCU GUU CUC AGA GCC GAU UUC GAC
AM883058        CAU UCU CAG ACC UGG GAC GUU GAU UCU GUG AAC GAU CCA GUU CUC AGA GCC GAC UUC GAC
AJ608217        UAU UCU CAG ACC UGG GAC GUU GAC GCU GUG AAC GAU CCC GUU CUC AGA GCC GAC UUC AAC
AJ608218        CAU UCU CAG ACC UGG GAC GUU GAC GCU GUG AAC GAU CCC GUU CUC AGA GCC GAC UUC AAC
AJ608216        CAU UCU CAG ACC UGG GAC GUU GAU UCU GUG AGA GAC CCC UCU CUC AGA GCC GAC UUC AAC
AJ608207        CAU UCU CAG ACC UGG GAC GUU GAC GCA GUG AAC GAU CCG GUU CUC AGC GCC GAC UUC AAC
FN432840        CAU UCU CAG ACC UGG GAC GUU GAC GCA GUG AAC GAU CCG GUU CUC AGC GCC GAC UUC AAC
AJ608219        CAU UCU CAG ACC UGG GAC GUU GAC GCA GUG AAC GAU CCA GUU CUC AGC GCC GAC UUC AAC
AJ608214        CAU UCU CAG ACC UGG GAC GUU GAC GCA GUG AAC GAU CCA GUU CUC AGC GCC GAC UUC AAC
AJ608208        CAU UCU CAG ACC UGG GAC GUU GAC GCA GUG AAC GAC CCC GUU CUC AGC GCU GAC UUC AAC
```

```
AJ608209        CAU UCU CAG ACC UGG GAC GUU GAC GCA GUG AAC GAU CCA GUU CUC AGC GCC GAC UUC AAC
                 **  *  *** * * *** *** **   *      *** *   **  **    * **      **  **  ***   *

AJ608206        CCU GAG AAG GUU GGU UGG GUG UCA GUG UCG UUC GCC UGU ACU CGG UGU ACA GCC CAC UAC
L20893          CCC GAG AAG GUU GGU UGG GUG UCA GUG UCG UUU GCC UGU ACU CGG UGU ACA GCC CAC UAC
FN432838        CCC GAG AGG GUU GGU UGG GUG UCC GUG UCG UUU GCC UGU ACU CAG UGU ACG GCU CAC UAC
FN432837        CCA GAG AGG GUU GGU UGG GUG UCA GUG UCG UUU GCC UGU ACU CAG UGU ACG GCG CAU UAC
U23142          CCG GAG AGG GUU GGU UGG GUG UCA GUG UCG UUU GCC UGU ACU CGG UGU ACG GCU CAC UAC
AJ608212        CCU GAG AGG GUU GGU UGG GUG UCU GUG UCG UUU GCC UGU ACU CAG UGC ACA GCC CAC UAC
FN432841        CCU GAG AAG GUU GGU UGG GUG UCU GUG UCG UUC GCC UGU ACU CAG UGC ACA GCC CAC UAC
AJ608213        CCU GAG AAG GUU GGU UGG GUG UCU GUG UCG UUU GCC UGU ACU CAG UGC ACA GCC CAC UAC
AJ608210        CCC GAG AGG GUU GGU UGG GUG UCU GUG UCA UUU GCC UGU ACU CGG UGC ACG GCU CAC UAC
AM883054        CCC GAG AGG GUU GGU UGG GUG UCU GUG UCA UUU GCC UGU ACU CGG UGC ACG GCU CAC UAC
AJ608211        CCC GAG AGG GUU GGU UGG GUG UCU GUG UCA UUU GCC UGU ACU CGG UGC ACG GCU CAC UAC
AM883055        CCC GAG AGG GUU GGU UGG GUG UCU GUG UCA UUU GCC UGU ACU CGG UGC ACG GCU CAC UAC
AM883056        CCC GAG AGG GCU GGU UGG GUG UCU GUG UCA UUU GCC UGU ACU CGG UGC ACG GCU CAC UAC
AJ608215        CCC GAC AGG GCC GGU UGG GUU GCU GUG UCG UUU GCC UGU ACC CAG UGC ACA GCU CAU UAC
AM883057        CCC GAU AAG GUU GGU UGG GUG ACC GUG UCG UUU GCC UGU ACC CAG UGC ACA GCU CAU CAC
FN432839        CCU GAU AGG GUU GGU UGG GUG ACC GUG UCG UUU GCC UGU ACC CAG UGC ACA GCU CAU CAC
AJ876793        CCC AAC GGG GUG GGC UGG GUG GCA GUG UCG UUU GCC UGU ACU CAG UGC ACG GCU CAC UAC
AJ877020        CCU AAC AGG UCG GGU UGG GUG GCU GUG UCG UUU GCC UGU ACU CAG UGC ACG GCU CAC UAC
AM883058        CCC AAC GGG GUU GGU UGG GUA GCU GUG UCG UUU GCC UGU ACU CAG UGC ACG GCU CAU UAC
AJ608217        CCU GAU AAG GUU GGU UGG GUG UCA GUG UCG UUU GCC UGU ACU CAG UGC ACG GCU CAC UAC
AJ608218        CCU GAA AAG GUU GGU UGG GUG UCA GUG UCG UUU GCC UGU ACU CAG UGC ACG GCU CAC UAC
AJ608216        CCC GAG AAG GUU GGU UGG GUG UCU GUG UCG UUC GCC UGU ACU CAG UGC ACG GCU CAC UAC
AJ608207        CCC ACA AAG GUU GGU UGG GUG UCA GUG UCG UUU GCC UGU ACU CGG UGC ACG GCU CAC UAC
FN432840        CCC ACA AAG GUU GGU UGG GUG UCA GUG UCG UUU GCC UGU ACU CGG UGC ACA GCU CAC UAC
AJ608219        CCC ACA AAG GUU GGU UGG GUG UCA GUG UCA UUU GCC UGU ACU CGG UGU ACA GCU CAC UAC
AJ608214        CCC GCG AAG GUU GGU UGG GUG UCA GUG UCG UUU GCC UGU ACU CGG UGU ACA GCU CAU UAC
AJ608208        CCC GAG AAG GUU GGU UGG GUG UCU GUG UCG UUU GCC UGU ACU CAG UGU ACG GCU CAC UAC
AJ608209        CCC GCA AAG GUU GGU UGG GUG UCA GUG UCG UUU GCC UGU ACU CGG UGU ACA GCU CAU UAC
                **        *     **  *** **   *  *** **  **  *** *** **  * * **  **  **  **   **

AJ608206        UAC ACG AGU GAG CAG GUG AAG UAU UUU GUU AAU AUU CCG CCU GUC CAU UAC GAC GUG GUG
L20893          UAC ACG UGU GAG CAG GUG AAA UAU UUC ACU AAU AUU CCG CCU GUU CAU UAC GAC GUG GUG
FN432838        UAC ACU UCU GAG CAG GUG AAA UAU UUC AUU AAU AUU CCG CCU GUC CAC UAC GAC GUG GUG
FN432837        UAC ACG UGU GAG CAG GUG AAA UAU UUC GUC AAU AUC CCA CCU GUU CAC UAC GAC GUG GUG
U23142          UAC ACG UGU GAG CAG GUG AAG UAU UUC GUG AAU AUA CCA CCU GUG CAC UAC GAC GUG GUG
AJ608212        UAC ACG UGU GAG CAG GUG AAG UGU UUC AUC AAU AUA CCG CCU GUU CAU UAC GAC GUG GUU
FN432841        UAC ACG UGU GAG CAG GUG AAG UGU UUC ACC AAU AUA CCG CCU GUU CAU UAC GAC GUG GUU
AJ608213        UAC ACG UGU GAG CAG GUG AAG UAU UUU ACC AAU AUA CCG CCU GUU CAU UAC GAC GUG GUU
AJ608210        UAU ACG UGU GAG CAG GUG AAA UUC UUC ACC AAC AUC CCG UCG GUU CAU UAC GAC GUG GUG
AM883054        UAU ACG UGU GAG CAG GUG AAA UUC UUC ACC AAC AUC CCG UCG GUU CAU UAC GAC GUG GUG
AJ608211        UAU ACG UGU GAG CAG GUG AAA UUC UUC ACC AAC AUC CCG UCG GUU CAU UAC GAC GUG GUG
AM883055        UAU ACG UGU GAG CAG GUG AAA UUC UUC ACC AAC AUC CCG UCG GUU CAU UAC GAC GUG GUG
AM883056        UAC ACG UGU GAG CAG GUG AAA UUC UUC ACC AAC AUC CCG UCG GUU CAU UAC GAC GUG GUG
AJ608215        UAC ACG UGU GAG CAG GUG AAA UUC UUC ACC AAC AUC CCG UCG GUU CAC UAC GAC GUG GUG
AM883057        UAU ACG UGU GAG CAG GUG AAG UUC UUC ACC AAC AUC CCG UCA GUU CAC UAC GAC GUG GUG
FN432839        UAU ACG UGU GAG CAG GUG AAG UUC UUC ACC GAC AUC CCG UCA GUU CAU UAC GAC GUG GUG
AJ876793        UAC ACG UGU GAG CAG GUG AAG UUC UUC ACC AAC AUC CCG UCG GUU CAU UAC GAC GUG GUG
AJ877020        UAU ACG UGU GAG CAG GUG AAG UUC UUC ACC AAC AUC CCG CCG AUU CAC UAC GAC GUG GUG
AM883058        UAU ACG UGU GAG CAG GUG AAG UUC UUC ACC AAC AUC CCG UCG GUU CAU UAC GAC GUG GUG
AJ608217        UAC ACG UGU GAG CAG GUG AAA CAC UUC ACC AAC AUC CCG UCG GUU CAU UAC GAC GUG GUG
AJ608218        UAU ACG UGU GAG CAG GUG AAA UUC UUC ACC AAC AUC CCG CCG GUU CAU UAC GAC GUG GUG
AJ608216        UAU ACG AGU GAG CAG GUG AAG UAU UUC ACC AAC AUC CCG CCG GUU CAU UUC GAC GUG GUG
AJ608207        UAC ACG AGU GAG CAG GUG AAG UGU UUC GUA AAC AUC CCA CCU GUC CAC UAC GAC GUG GUG
FN432840        UAC ACG AGU GAG CAG GUG AAG UGU UUC GUA AAC AUC CCA CCU GUC CAC UAC GAC GUG GUG
AJ608219        UAU ACG AGU GAG CAG GUG AAG UGU UUC GUU AAC AUC CCA CCU GUC CAU UAC GAC GUA GUG
AJ608214        UAU ACG AGU GAG CAG GUG AAG UGU UUU GUC AAC AUC CCA UCU GUC CAC UAC GAC GUG GUG
AJ608208        UAC ACA AGU GAG CAG GUG AAA UAC UUU ACC AAU AUU CCG CCU GUC CAC UAC GAC GUG CUG
AJ608209        UAC ACU AGU GAG CAG GUG AAG UGU UUU GUC AAC AUC CCA UCU GUC CAC UAC GAC GUG GUG
                **  **    * *** *** *** **      **       *  **  **   *   *  **  * * *** **   *

AJ608206        UGC GCU GAU UGC GAG CGU AGU GUU CAG CAG GAC GAC GAG AUC GAC CGC GAG CAC GAC GAG
L20893          UGC GCU GAU UGC GAG CGU AGU GUU CAG CAG GAC GAC GAG AUC GAC CGC GAG CAC AAC GAG
FN432838        UGU GCC GAU UGC GAG CGU AGU GUU CAG CUG GAC GAC GAG AUC GAC CGC GAA CAC AAC GAG
FN432837        UGU GCU GAU UGC GAG CGC AGU GUU CAG CAG GAC GAC GAA AUC GAC CGC GAA CAC AAC GAG
U23142          UGU GCC GAU UGC GAG CGC AGU GUU CAA GAG GAC GAC GAG AUC GAC CGU GAG CAC AAC GAG
AJ608212        UGU GCU GAU UGC GAG CGC AGU GUU CAA CAG GAC GAC GAG GUC GAC CGU GAG CAC GAC GAG
FN432841        UGU GCU GAU UGC GAG CGC AGU GUU CAA CAG GAC GAC GAG AUC GAC CGU GAG CAC GAC GAG
AJ608213        UGU GCU GAU UGC GAG CGC AGU GUU CAA CAG GAC GAC GAG AUC GAC CGU GAG CAC GAC GAG
AJ608210        UGU GCC GAU UGU GAG CGC AGU GUU CAG CAG GAC GAC GAG AUC GAC CGC GAA CAC GAC GAG
AM883054        UGU GCC GAU UGU GAG CGC AGU GUU CAG CAG GAC GAC GAG AUC GAC CGC GAA CAC GAC GAG
AJ608211        UGU GCU GAU UGU GAG CGC AGU GUU CAG CAG GAC GAC GAG AUC GAC CGC GAA CAC GAC GAG
```

```
AM883055        UGU GCU GAU UGU GAG CGC AGU GUU CAG CAG GAC GAC GAG AUC GAC CGC GAA CAC GAC GAG
AM883056        UGU GCU GAU UGU GAG CGC AGU GUU CAG CUG GAC GAC GAG AUC GAC CGC GAA CAC GAC GAG
AJ608215        UGU GCU GAU UGC GAG CGC CGU GUU CAG CAG GAC GAC GAG AUC GAC CGU GAG CAC CAA GAG
AM883057        UGU GCU GAU UGC GAG CGC CGU GUU CAG CAG GAC GAC GAG AUC GAC CGU GAG CAC CAA GAG
FN432839        UGU GCU GAU UGC GAG CGC CGU GUU CAG CGG GAC GAC GAG AUC GAC CGU GAG CAC CAA GAG
AJ876793        UGU GCU GAU UGC GAG CGC CGU GUU CAG CAG GAC GAC GAG AUC GAC CGU GAG CAC CAA GAG
AJ877020        UGU GCU GAU UGC GAG CGA CGU GUU CAG CAG GAC GAC GAG AUC GAC CGU GAA CAC CAA GAG
AM883058        UGU GCU GAU UGC GAG CGC CGU GUU CAG CAG GAC GAC GAG GUC GAC CGU GAG CAC CAA GAG
AJ608217        UGU GCU GAU UGC GAG CGU GGU GUU CAG CUG GAC GAC GAA AUC GAC CGC GAA CAC CAA GAG
AJ608218        UGU GCC GAU UGC GAG CGU AGU GUU CAG CUG GAC GAC GAG AUC GAC CGC GAA CAC CAA GAG
AJ608216        UGU GCC GAU UGC GAG CGU AGU GUU CAA UUG GAC GAC GAG AUC GAC CGC GAA CAC CAA GAG
AJ608207        UGU GCU GAU UGC GAG CGC AGU GUU CAG CAG GAC GAC CAG AUC GAC CGC GAG CAC AAC GAG
FN432840        UGU GCU GAU UGC GAG CGC AGU GUU CAG CAG GAC GAC CAG AUC GAC CGU GAG CAC AAC GAG
AJ608219        UGU GCU GAU UGC GAG CGC AGU GCU CAG CAG GAC GAG GAG AUC GAC CGU GAG CAC AAC GAG
AJ608214        UGU GCU GAU UGC GAG CGC AGU GUU CAG CAG GAC GAC CAG AUC GAC CGC GAG CAC GAC GAG
AJ608208        UGU GCC GAU UGU GAG CGC AGU GUU CAG CAG GAC GAC GAG AUC GAC CGU GAA CAC GAC GAG
AJ608209        UGU GCU GAU UGC GAG CGC AGU GUU CAG CAG GGC GAC GAG AUC GAC CGC GAG CAC GAC GAG
                **  **  *** **  *** **   ** * * **     * * * **   *   ** *** **  **  ***  *  ***

AJ608206        CGU AAC GCA GAG AUU UCU GCC UGC AAC GCU CGA GCC CUA AGU GAG GGG AGA CCG GCA AGU
L20893          CGU AAC GCA GAG AUU UCU GCC UGC AAC GCU CGA GCC CUA AGU GAG GGA AGG CCA GCG AGU
FN432838        CGU AAC GCA GAG AUU UCU GCC UGC AAC GCC CGA GCU CUG AGC GAG GGG AGA CCA GCA AGU
FN432837        CGU AAC GCA GAG AUU UCC GCC UGC AAC GCU CGG GCU CUG AGU GAA GGG CGG CCA GCA ACU
U23142          CGU AAC GCA GAG AUU UCU GCC UGC AAC GCU CGG GCU CUG AGU GAG GGC CGG CCA GCA ACC
AJ608212        CGU AAC GCA GAG AUC UCU GCC UGC AAC GCU CGG GCU CUG AGC GAG GGG AGG CCA GCA ACC
FN432841        CGU AAC GCA GAG AUC UCC GCC UGC AAC GCU CGG GCU CUG AGC GAG GGG AGG CCA GCA ACU
AJ608213        CGU AAC GCA GAG AUC UCU GCC UGC AAC GCU CGG GCU CUG AGC GAG GGG AGG CCA GCA ACC
AJ608210        CGU AAC GCA GAG AUU UCU GCC UGC AAC GCU CGG GCU UUG AGU GAG GGU CGA CCA GCC AGU
AM883054        CGU AAC GCA GAG AUU UCU GCC UGC AAC GCU CGG GCU UUG AGU GAG GGU CGA CCA GCC AGU
AJ608211        CGU AAC GCA GAG AUU UCU GCC UGC AAC GCU CGG GCU UUG AGU GAG GGU CGA CCA GCC AGU
AM883055        CGU AAC GCA GAG AUU UCU GCC UGC AAC GCU CGG GCU UUG AGU GAG GGU CGA CCA GCC AGU
AM883056        CGU AAC GCA GAG AUU UCU GCC UGC AAC GCU CGG GCU UUG AGU GAG GGU CGA CCA GCC AGU
AJ608215        CGU AAC GCA GAG CUA UCU GCU UGU AAC GCU AGG GCC UUG AGU GAG GGA AGA CCA GCC AGU
AM883057        CGC AAC GCA GAA AUU UCU GCU UGC AAC GCU CGA GCU UUG AGC GAG GGA AGA CCA GCA AGU
FN432839        CGU AAC GCA GAA AUC UCU GCU UGC AAC GCU CGA GCU UUG AGU GAG GGA AGA CCA GCA AGU
AJ876793        CGC AAC GCA GAG AUU UCU GCC UGC AAC GCU CGA GCU UUG AGC GAG GGA AGA CCA GCA AGC
AJ877020        CGG AAC GCA GAG AUU UCU GCC UGC AAC GCU CGA GCU UUG AGU GAG GGG AAG CCA GCA AGU
AM883058        CGC AAC GCA GAG AUU UCC GCC UGC AAC GCU CGA GCC UUG AGU GAG GGG AGG CCA GCA AGU
AJ608217        CGU AAC GCA GAG AUU UCU GCC UGC AAC GCU CGA GCU UUG AGU GAA GGG CGA CCA GCU AGU
AJ608218        CGU AAC GCA GAG AUU UCU GCC UGC AAC GCU CGG GCC UUA AGU GAG GGA CGG CCA GCU AGC
AJ608216        CGU AAC GCA GAG AUU UCU GCC UGC AAC GCU CGG GCC UUG AGU GAA GGA AGA CCA GCA AGU
AJ608207        CGU AAC GCA GAG AUC UCU GCC UGC AAC GCU CGG GCC CUG AGC GAG GGU AGA CCA GCA AGU
FN432840        CGU AAC GCA GAG AUU UCC GCC UGC AAC GCU CGG GCC CUG AGC GAG GGU AGA CCA GCA AGU
AJ608219        CGU AAC GCA GAG AUU UCU GCC UGC AAC GCU CGG GCC CUG AGU GAA GGU AGA CCA GCA AGU
AJ608214        CGU AAC GCA GAG AUU UCU GCC UGC AAC GCU CGG GCC CUG AGU GAG GGU AGA CCA GCA AGU
AJ608208        CGU AAC GCA GAG AUC UCU GCC UGC AAC GCU CGG GCU CUG AGU GAA GGG AGA CCA GCA AGU
AJ608209        CGU AAC GCA GAG AUU UCU GCC UGC AAC GCU CGG GCC CUG AGU GAG GGU AGA CCA GCA AGU
                **  *** *** **   *  **  **  **  *** **   *  **   *  **  **  **      **  **  *

AJ608206        CUG GUU UAC CUC UCG CGG GAC GCU UGC GAU AUA CCC GAG CAC UCC GGA ACG UGC CGG UUU
L20893          CUG GUU UAC CUU UCG CGG GAC GCU UGU GAU AUA CCC GAG CAC UCC GGA ACG UGU CGG UUC
FN432838        UUG GUC UAC CUU UCU CGA GAC GCU UGC GAC AUA CCU GAG CAC UCC GGA ACG UGC CGG UUU
FN432837        CUG GUC UAC CUC UCU CGG GAC GCU UGC GAU AUA CCU GAG CAC UCC GGA ACG UGC CGG UUC
U23142          CUG GUU UAC CUC UCU CGA GAC GCU UGU GAU AUC CCC GAG CAC UCC GGU ACG UGC CGG UUC
AJ608212        CUG GUA UAC CUC UCU CGA GAC GCU UGC GAC AUU CCU GAG CAC UCC GGA ACG UGC CGG UUU
FN432841        CUG GUU UAC CUC UCU CGA GAC GCU UGC GAU AUC CCU GAG CAC UCC GGA ACG UGC CGG UUU
AJ608213        CUG GUU UAC CUU UCU CGA GAC GCU UGC GAU AUU CCU GAG CAC UCU GGA ACG UGC CGG UUC
AJ608210        CUG GUU UAC CUC UCU CGG GAC GCU UGC GAU AUC CCC GAG CAC GCC GGA AGC UGC CGG UUU
AM883054        CUG GUU UAC CUC UCU CGG GAC GCU UGC GAU AUC CCC GAG CAC GCC GGA AGC UGC CGG UUU
AJ608211        CUG GUU UAC CUC UCU CGG GAC GCU UGC GAU AUC CCC GAG CAC GCC GGA AGC UGC CGG UUU
AM883055        CUG GUU UAC CUC UCU CGG GAC GCU UGC GAU AUC CCC GAG CAC GCC GGA AGC UGC CGG UUU
AM883056        CUG GUU UAC CUC UCU CGG GAC GCU UGC GAC AUU CCC GAG CAC GCC GGA AGC UGC CGG UUU
AJ608215        CUG GUG UAU CUC UCU CGG GAC GCU UGU GAU AUC CCU GGG CAC UCC GGA ACG UGC CGG UAU
AM883057        CUG GUG UAC CUU UCU CGG GAC GCU UGU GAU AUC CCU GGG CAC UCC GGA ACG UGC CGG UAU
FN432839        CUG GUG UAC CUU UCU CGG GAC GCU UGU GAU AUC CCU GGG CAC UCC GGA ACG UGC CGG UAC
AJ876793        CUG GUG UAC CUC UCU CGG GAC GCU UGU GAU AUU CCU GAG CAC UCC GGA ACG UGC CGG UAC
AJ877020        CUG GUA UAC CUC UCU CGA GAC GCU UGC GAU AUA CCU GAG CAC UCC GGA ACG UGC CGG UAC
AM883058        CUG GUA UAC CUC UCU CGG GAC GCU UGU GAU AUC CCU GAG CAC UCC GAA ACG UGU CGG UUC
AJ608217        CUG GUU UAC CUC ACU CGG GAC GCU UGC GAU AUC CCU GAG CAC UCC GGA AGG UGC CGG UUU
AJ608218        CUG GUA UAC CUC UCU CGG GAC GCU UGU GAC AUU CCU GAG CAC UCC GGA AGG UGC CGG UUU
AJ608216        UUG GUU UAC CUC UCU CGG GAC GCU UGU GAU AUU CCU GAG CAC UCC GGA AGG UGC CGG UUU
AJ608207        UUG GUU UAU CUU UCA CGG GAC GCU UGU GAC AUA CCC GAG CAC UCC GGA ACG UGC CGG UUU
FN432840        UUG GUU UAU CUU UCA CGG GAC GCU UGU GAC AUA CCC GAG CAC UCC GGA ACG UGC CGG UUU
AJ608219        UUG GUU UAU CUU UCG CGG GAC GCU UGU GAU AUA CCC GAG CAC UCC GGA ACG UGC CGG UUU
```

```
AJ608214        UUG GUU UAU CUU UCA CGG GAC GCU UGU GAC AUA CCC GAG CAC UCC GGA ACG UGC CGG UUU
AJ608208        UUG GUU UAC CUC UCU CGG GAC GCU UGC GAC AUA CCU GAG CAC UCC GGA ACG UGC CGG UUU
AJ608209        UUG GUU UAU CUU UCA CGG GAC GCU UGU GAU AUA CCC GAG CAC UCC GGC ACG UGC CGG UUC
                 ** **  **  **   *  **  *** *** **  **  **  **  * * ***  *  *   *   **  *** *

                                        ORF1 stop         Likely ORFx start (CUG with a purine at
                                                          '-3' for a strong context in monocots)
AJ608206        GAC AAA UAU CUC AAC UUC UGA GAG AAU CAG UCC UGC CGA CAU CUA AGG AGC GGU CUU GGU
L20893          GAC AAA UAU CUC AAC UUU UGA GAG AAU CAG UCC UGC CGA CAU CUA AGG AGC GGU CUU GGU
FN432838        GAC AAG UAC CUC AAC UUU UGA GGG AAU CAG UCC UGC CGA CGU CUA AGG AGC GGU CUU GGU
FN432837        GAC AAG UAC CUC AAC UUU UGA GGG A-U CAG UCC UGC CGA CAU CUA AGG AGC GGU CUU GGU
U23142          GAC AAG UAC CUC AAU UUC UGA GGG AAU CAG UCC UGC CGA CAU CUA AGG AGC GGU CUU GGU
AJ608212        GAU AAG UAC CUC AAC UUU UGA GGG A-C CAG UCC UGC CGA CAU CCA AGG AGC GGU CGU GGU
FN432841        GAU AAG UAC CUC AAC UUC UGA GGG A-C CAG UCC UGC CGA CAU CCA AGG AGC GGU CGU GGU
AJ608213        GAU AGG UAC CUC AAC UUU UGA GGA A-C CAG UCC UGC CGA CAU CCA AGG AGC GGU CGU GGU
AJ608210        GAU AAG UAC CUC AAC UUU UGA GGG AAC --A CUC UGC CGA CAU CCA AGG AGC GGU CGU GGU
AM883054        GAU AAG UAC CUC AAC UUU UGA GGG AAC --A CUC UGC CGA CAU CCA AGG AGC GGU CGU GGU
AJ608211        GAU AAG UAC CUC AAC UUU UGA GGG AAC --A CUC UGC CGA CAU CCA AGG AGC GGU CGU GGU
AM883055        GAU AAG UAC CUC AAC UUU UGA GGG AAC --A CUC UGC CGA CAU CCA AGG AGC GGU CGU GGU
AM883056        GAU AAG UAC CUC AAC UUU UGA GGG AAC --A UUC UGC CGA CAU CCA AGG AGC GGU CGU GGU
AJ608215        AAC AGG UAC CUC AAC UUU UGA GAG UGC C-G ACC UGC CGA CAU CCA AGG AAC GGU CUU GGC
AM883057        AAC AGG UAC CUC AAC UUU UGA GGG UGC C-A ACC UGC CGA CAU CCA AGG AAC GGU CUU GGC
FN432839        AAC AGG UAC CUC AAC UUU UGA GGU CGC C-A ACC UGC CGA CAU CUA AGG AAC GGU CUU GGC
AJ876793        GAC AGG UAC CUC AAC UUU UGA GG- -GC C-A GCC UGC CGA CAU CCA AGG AAC GGU CUU GGU
AJ877020        GAC AAG UAC CUC AAC UUU UGA GGG CGU C-A GUC UGC CGA CAU CCA AGG AAC GGU CUU GGU
AM883058        GAC AAG UAC CUC AAC UUU UGA GGA AGC C-A GUC UGC CGA CAU CCA AGG AAC GGU CUU GGC
AJ608217        GUU AAG UAC CUC AAC UUU UGA GGG A-C GCA AUC UGC CGA CAU CCA AGG AAC GGU CGU GGU
AJ608218        GUU AAG UAC CUC AAC UUC UGA GGG AAC ACA ACC UGC CGA CAU CCA AGG AAC GGU CGU GGU
AJ608216        GUU AAG UAC CUC AAU UUC UGA GGA AAC --A ACC UGC CGA CAU CCA AGG AAC GGU CUU GGU
AJ608207        GAC AAA UAC CUC AAC UUU UGA GG- AAU CAG UCC UGC CGA CAU CCA AGG AGC GGU CUU GGU
FN432840        GAC AAA UAC CUC AAC UUU UGA GG- AAU CAG UCC UGC CGA CAU CUA AGG AGC GGU CUU GGU
AJ608219        GAC AAA UAC CUC AAC UUU UGA GGG AAU CAG UCC UGC CGA CAU CUA AGG AGC GGU CUU GGU
AJ608214        GAC AAA UAC CUC AAC UUU UGA GGG AAU CAG UCC UGC CGA CAU CCA AGG AGC GGU CUU GGC
AJ608208        GAC AAG UAC CUC AAU UUC UGA G-G GAU CAG UCC UGC CGA CAU CUA AGG AGC GGU CUU GGU
AJ608209        GAC AAA UAC CUC AAC UUC UGA GG- AAU CAG UCC UGC CGA CAU CCA AGG AGC GGU CUU GGC
                    *   **  *** **  **  *** *             *  *** *** * * * * *** * * *** * * **
                                                                     ^   ^       ^       ^
                                                Variability at 3rd positions of ORFx-frame codons

                                    ORF2a (AUG2; strong context)
AJ608206        UCA GAC UAA CGC GGG AUG GGC UCU UCU GUU GUU GGU CGC UUU CUC ACU CGC ACC AAG GAA
L20893          UCA GAC UAA CGC GGG AUG GGC UCU UCU GUU GUU GGU CGC UUU CUC ACU CGC ACC AAG GAA
FN432838        UCA GAC UAA CAC GGG AUG GGC UCU UCU GUU GUU GGU CGC UUU CUC ACU CGC ACC AAG GAA
FN432837        UCA GAC UAA CAC GGG AUG GGC UCU UCU GUU GUU GGU CGC UUU CUC ACU CGC ACC AAG GAA
U23142          UCA GAC UGA CAC GGG AUG GGC UCU UCU GUU GUU GGU CGC UUU CUC ACU CGC ACC AAG GAG
AJ608212        UUA GAC UAA CAC GGG AUG GGC UCU UCU GUU GUU GGU CGC UUU CUC ACU CGC ACC AAG GAA
FN432841        UCA GAC UAA CAC GGG AUG GGC UCU UCU GUU GUU GGU CGC UUU CUC ACU CGC ACC AAG GAA
AJ608213        UCA GAC UAA CAC GGG AUG GGC UCU UCU GUU GUU GGU CGC UUU CUC ACU CGC AUC AAG GAA
AJ608210        UCA GAC UAA CAC GGG AUG GGC UCU UCU GUU GUU GGU CGC UUU CUC ACU CGC ACC AAG GAA
AM883054        UCA GAC UAA CAC GGG AUG GGC UCU UCU GUU GUU GGU CGC UUU CUC ACU CGC ACC AAG GAA
AJ608211        UCA GAC UAA CAC GGG AUG GGC UCU UCU GUU GUU GGU CGC UUU CUC ACU CGC ACC AAG GAA
AM883055        UCA GAC UAA CAC GGG AUG GGC UCU UCU GUU GUU GGU CGC UUU CUC ACU CGC ACC AAG GAA
AM883056        UCA GAC UAA CAC GGG AUG GGC UCU UCU GUU GUU GGU CGC UUU CUC ACU CGC ACC AAG GAA
AJ608215        UUA GAC UGA CAC GGG AUG GGC UCU UCU GUU GUU GGU CGC UUU CUC ACU CGA ACC AAG GAA
AM883057        UCA GAC UAA CAC GGG AUG GGC UCU UCU GCU GUU GGU CGC UUU CUC ACU CGA ACC AAG GAA
FN432839        UCA GAC UAA CAC GGG AUG GGC UCU UCU GUU GUU GGU CGC UUU CUC ACU CGA ACC AAG GAA
AJ876793        UCA GAC UAA CGC GGG AUG GGC UCU UCU GUU GUU GGU CGC UUU CUC ACA CGC ACC AAG GAA
AJ877020        UCA GAC UAA CAC GGG AUG GGC UCU UCU GUU GUU GGU CGC UUU CUC ACU CGC ACC AAG GAA
AM883058        UCA GAC UAA CAC GGG AUG GGC UCU UCU GCU GUU GGU CGC UUU CUC ACU CGA ACC AAG GAA
AJ608217        UCA GAC UAA CGC GGG AUG GGC UCU UCU GUU GUU GGU CGC UUU CUC ACU CGC ACC AAG GAA
AJ608218        UCA GAC UGA CAC GGG AUG GGC UCU UCU GUU GUU GGU CGC UUU CUC ACU CGC ACC AAG GAA
AJ608216        UCA GAC UAA CUC GGG AUG GGC UCU UCU GUU GGU GGU CGC UUU CUC ACU CGC ACC AAG GAA
AJ608207        UCA GAC UAA CAC GGG AUG GGC UUU UCU GUU GUU GGU CGC UUU CUC ACU CGC ACC AAG GAA
FN432840        UCA GAC UAA CAC GGG AUG GGC UUU UCU GUU GUU GGU CGC UUU CUC ACU CGC ACC AAG GAA
AJ608219        UCA GAC UAA CAC GGG AUG GGC UUU UCU GUU GUU GGU CGC UUU CUC ACU CGC ACC AAG GAA
AJ608214        UCA GAC UGA CAC GGG AUG GGC UCU UCU GUU GUU GGU CGC UUU CUC ACU CGC ACC AAG GAA
AJ608208        UCA GAC UAA CGC GGG AUG GGC UCU UCU GUU GUU GGU CGC UUU CUC ACU CGC ACC AAG GAA
AJ608209        UUA GAC UGA CAC GGG AUG GGC UCU UCU GUU GUU GGU CGC UUU CUC ACU CGC ACC AAG GAA
                * * *** * * * * *** *** *** * * *** * * * * *** *** *** *** **  **  * * *** **
                 ^       ^   ^               ^       ^   ^                    ^   ^  ^        ^
                                                Mixed coding signature in the overlap region

AJ608206        ACG ACG AGG GUG GAC UCC CAC CUC CUC AUC GUC UUG GCU GGG UUG AUA GCG GCC GCG AUA
L20893          ACG ACG AGG GUG GAC UCC CAC CUU CUC AUC GUC UUG GCU GGG UUG AUA GCG GCC GCG AUA
```

```
FN432838       ACG ACG AGG GUG GAC UCC AAC CUC CUC AUC GUC UUG GCU GGG UUG AUA GCG GCC GCG AUA
FN432837       ACG ACG AGG GUG GAC UCC AAC CUC CUC AUC GUC UUG GCU GGG UUG AUA GCG GCC GCG AUA
U23142         ACA ACG AGG GUG GAC UCC AAC CUC CUC AUC GUC UUG GCU GGG UUG AUA GCG GCC GCG AUA
AJ608212       ACG ACG AGG GUG GAC UCC AGC CUC CUC AUC GUC UUG GCU GGG UUG AUA GCG GCC GCG AUA
FN432841       ACG ACG AGG GUG GAC UCC AGC CUC CUC AUC GUC UUG GCU GGG UUG AUA GCG GCC GCG AUA
AJ608213       ACG ACG AGG GUG GAC UCC AGC CUC CUC AUC GUC UUG GCU GGG UUG AUA GCG GCC GCG AUA
AJ608210       ACG ACG AGG GUG GAC UCC AAC CUC CUC AUC GUC UUG GCU GGG UUG AUA GCG GCC GCG AUA
AM883054       ACG ACG AGG GUG GAC UCC AAC CUC CUC AUC GUC UUG GCU GGG UUG AUA GCG GCC GCG AUA
AJ608211       ACG ACG AGG GUG GAC UCC AAC CUC CUC AUC GUC UUG GCU GGG UUG AUA GCG GCC GCG AUA
AM883055       ACG ACG AGG GUG GAC UCC AAC CUC CUC AUC GUC UUG GCU GGG UUG AUA GCG GCC GCG AUA
AM883056       ACG ACG AGG GUG GAC UCC AAC CUC CUC AUC GUC UUG GCU GGG UUG AUA GCG GCC GCG AUA
AJ608215       ACG ACG AGG GUG GAU UCC AAC CUC CUC AUC GUC UUG GCU GGG UUG AUA GCG GCC GCG AUA
AM883057       ACG ACG AGG GUG GAC UCC AAC CUC CUC AUC GUC UUG GCU GGG UUG AUA GCG GCC GCG AUA
FN432839       ACG ACG AGG GUG GAU UCC AAC CUC CUC AUC GUC UUG GCU GGG UUG AUA GCG GCC GCG AUA
AJ876793       ACG ACG AGG GUG GAU UCC AAC CUC CUC AUC GCC UUG GCU GGG UUG AUA GCG GCC GCG AUA
AJ877020       ACG ACG AGG GUG GAU UCC AAC CUC CUC AUC GUC UUG GCU GGG UUG AUA GCG GCC GCG AUA
AM883058       ACG ACG AGG GUG GAC UCC AAC CUC CUC AUC GUC UUG GCU GGG UUG AUA GCG GCC GCG AUA
AJ608217       ACG ACG AGG GUG GAC UCC AAC CUC CUC AUC GUC UUG GCU GGG UUG AUA GCG GCC GCG AUA
AJ608218       ACG AUG AGA GUG GAC UCC AAC CUC CUC AUC GUC UUG GCU GGG UUG AUA GCG GCC GCG AUA
AJ608216       ACG ACG AGG GUG GAC UCC AAC CUC CUC AUC GUC UUG GCU GGG UUG AUA GCG GCC GCG AUA
AJ608207       ACG ACG AGG GUG GAC UCC AAC CUC CUC AUC GUC UUG GCU GGG UUG AUA GCG GCC GCG AUA
FN432840       ACG ACG AGG GUG GAC UCC AAC CUC CUC AUC GUC UUG GCU GGG UUG AUA GCG GCC GCG AUA
AJ608219       ACG ACG AGG GUG GAC UCC AAC CUC CUC AUC GUC UUG GCU GGG UUG AUA GCG GCC GCG AUA
AJ608214       GCG ACG AGG GUG GAC UCC AAC CUC CUC AUC GUC UUG GCU GGG UUG AUA GCG GCC GCG AUA
AJ608208       ACG ACG AGG GUG GAC UCC AAC CUC CUC AUC GUC UUG GCU GGG UUG AUA GCG GCC GCG AUA
AJ608209       ACG ACG AGG GUG GAC UCC AAC CUC CUC AUC GUC UUG GCU GGG UUG AUA GCG GCC GCG AUA
                *  * * **  *** **  ***   * **  *** *** * * *** *** *** *** *** *** *** *** ***

AJ608206       GUC UCA GAG CGC CUG CCC GUG ACG GCG UCA UUG UGG GCA AUC CCC UCA GCU AUC AUA GCA
L20893         GUC UCA GAG CGC CUA CCC GUG ACG GCG UCA UUG UGG GCA AUC CCC UCA GCU AUC AUA GCA
FN432838       GUC UCA GAG CGC CUG CCC GUG ACG GCG UCA UUG UGG GCA AUC CCC UCA GCU AUC AUA GCA
FN432837       GUC UCA GAG CGC CUG CCC GUG ACG GCG UCA UUG UGG GCA AUC CCC UCA GCU AUC AUA GCA
U23142         GUC UCA GAG CGC CUA CCC GUG ACG GCG UCA UUG UGG GCA AUC CCC UCA GCU AUC AUA GCA
AJ608212       GUC UCA GAG CGC CUG CCC GUG ACG GCG UCA UUG UGG GCA AUC CCC UCA GCU AUC AUA GCA
FN432841       GUC UCA GAG CGC CUG CCC GUG ACG GCG UCA UUG UGG GCA AUC CCC UCA GCU AUC AUA GCA
AJ608213       GUC UCA GAG CGC CUG CCC GUG ACG GCG UCA UUG UGG GCA AUC CCC UCA GCU AUC AUA GCA
AJ608210       GUC UCA GAG CGC CUG CCC GUG ACG GCG UCA UUG UGG GCA AUC CCC UCA GCU AUC AUA GCA
AM883054       GUC UCA GAG CGC CUG CCC GUG ACG GCG UCA UUG UGG GCA AUC CCC UCA GCU AUC AUA GCA
AJ608211       GUC UCA GAG CGC CUG CCC GUG ACG GCG UCA UUG UGG GCA AUC CCC UCA GCU AUC AUA GCA
AM883055       GUC UCA GAG CGC CUG CCC GUG ACG GCG UCA UUG UGG GCA AUC CCC UCA GCU AUC AUA GCA
AM883056       GUC UCA GAG CGC CUG CCC GUG ACG GCG UCA UUG UGG GCA AUC CCC UCA GCU AUC AUA GCA
AJ608215       GUC UCA GAG CGC CUA CCC GUG ACG GCG UCA UUG UGG GCA AUC CCC UCA GCU AUC AUA GCA
AM883057       GUU UCA GAG CGC CUA CCC GUG ACG GCG UCA UUG UGG GCA AUC CCC UCA GCU AUC AUA GCA
FN432839       GUU UCA GAG CGC CUA CCC GUG ACG GCG UCA UUG UGG GCA AUC CCC UCA GCU AUC AUA GCA
AJ876793       GUC UCA GAG CGC CUG CCC GUG ACG GCG UCA UUG UGG GCA AUC CCC UCA GCU AUC AUA GCA
AJ877020       GUC UCA GAG CGU CUA CCC GUG ACG GCG UCA UUG UGG GCA AUC CCC UCA GCU AUC AUA GCA
AM883058       GUU UCA GAG CGC CUA CCC GUG ACG GCG UCA UUG UGG GCA AUC CCC UCA GCU AUC AUA GCA
AJ608217       GUA UCA GAG CGC CUA CCC GUG ACG GCG UCA UUG UGG GCA AUC CCC UCA GCU AUC AUA GCA
AJ608218       GUC UCA GAG CGU CUA CCC GUG ACG GCG UCA UUG UGG GCA AUC CCC UCA GCU AUC AUA GCA
AJ608216       GUC UCA GAG CGC CUA CCC GUG ACG GCG UCA UUG UGG GCA AUC CCC UCA GCU AUC AUA GCA
AJ608207       GUC UCA GAG CGC CUG CCC GUG ACG GCG UCA UUG UGG GCA AUC CCC UCA GCU AUC AUA GCA
FN432840       GUC UCA GAG CGC CUG CCC GUG ACG GCG UCA UUA UGG GCA AUC CCC UCA GCU AUC AUA GCA
AJ608219       GUC UCA GAG CGC CUG CCC GUG ACG GCG UCA UUG UGG GCA AUC CCC UCA GCU AUC AUA GCA
AJ608214       GUC UCA GAG CGU CUG CCC GUG ACG GCG UUA UUG UGG GCA AUC CCC UCA GCU AUC AUA GCA
AJ608208       GUG UCA GAG CAC CUG CCC GUG ACG GCG UCA UUG UGG GCA AUC CCC UCA GCU AUC AUA GCA
AJ608209       GUC UCA GAG CGC CUG CCC GUG ACG GCG UUA UUG UGG GCA AUC CCC UCA GCU AUC AUA GCA
               **  *** *** *    **  *** *** *** *** * * **  *** *** *** *** *** *** *** *** ***

                                                                      ORFx stop
AJ608206       AAC UGG AUC GUG CUG UCA GCU CAC GAG AGC UUC AGC CGG UUC GUU GAG GGG GUU GAG AUU
L20893         AAC UGG AUC GUG CUG UCA GCU CAC GAG AGC UUC AGC CGG UUC GUU GAG GGG GUU GAG AUU
FN432838       AAC UGG AUC GUG UUG UCA GCU CAC GAG AGC UUC AGC CGG UUC GUU GAG GGG GUU GAG AUU
FN432837       AAC UGG AUC GUG CUG UCA GCU CAC GAG AGC UUC AGC CGG UUC GUU GAG GGG GUU GAG AUU
U23142         AAC UGG AUC GUG CUG UCA GCU CAC GAG AGC UUC AGC CGG UUC GUU GAG GGG GUU GAG AUU
AJ608212       AAU UGG AUC GUG CUG UCA GCU CAC GAG AGC UUC AGC CGG UUC GUU GAG GGG GUU GAG AUU
FN432841       AAU UGG AUC GUG CUG UCA GCU CAC GAG AGC UUC AGC CGG UUC GUU GAG GGG GUU GAG AUU
AJ608213       AAU UGG AUC GUG CUG UCA GCU CAC GAG AGC UUC AGC CGG UUC GUU GAG GGG GUU GAG AUU
AJ608210       AAC UGG AUC GUG CUG UCA GCU CAC GAG AGC UUC AGC CGG UUC GUU GAG GGG GUU GAG AUU
AM883054       AAC UGG AUC GUG CUG UCA GCU CAC GAG AGC UUC AGC CGG UUC GUU GAG GGG GUU GAG AUU
AJ608211       AAC UGG AUC GCG CUG UCA GCU CAC GAG AGC UUC AGC CGG UUC GUU GAG GGG GUU GAG AUU
AM883055       AAC UGG AUC GCG CUG UCA GCU CAC GAG AGC UUC AGC CGG UUC GUU GAG GGG GUU GAG AUU
AM883056       AAC UGG AUC GCG CUG UCA GCU CAC GAG AGC UUC AGC CGG UUC GUU GAG GGG GUU GAG AUU
AJ608215       AAC UGG AUC GUG CUG UCA GCU CAC GAG AGC UUC AGC CGG UUC GUG GAG GGG GUU GAG GUU
AM883057       AAU UGG AUC GUG CUG UCA GCU CAC GAG AGC UUC AGC CGG UUC GUG GAG GGG GUU GAG GUU
```

```
FN432839        AAU UGG AUC GUG CUG UCA GCU CAC GAG AGC UUC AGC CGG UUC GUG GAG GGG GUU GAG GUU
AJ876793        AAC UGG AUC GUG CUG UCA GCU CAC GAG AGC UUC AGC CGG UUC GUG GAG GGG GUU GAG GUU
AJ877020        AAC UGG AUC GUG CUG UCA GCU CAC GAG AGC UUC AGC CGG UUC GUG GAG GGG GUU GAG AUU
AM883058        AAU UGG AUC GUG CUG UCA GCU CAC GAG AGC UUC AGC CGG UUC GUG GAG GGG GUU GAG GUU
AJ608217        AAC UGG AUC GUG CUA UCA GCU CAC GAG AGC UUC AGC CGG UUC GUU GAG GGG GUU GAG AUU
AJ608218        AAC UGG AUC GUG CUA UCA GCU CAC GAG AGC UUC AGC CGG UUC GUU GAG GGG GUU GAG AUU
AJ608216        AAC UGG AUC GUG CUG UCA GCU CAC GAG AGC UUC AGC CGG UUC GUU GAG GGG GUU GAG AUU
AJ608207        AAC UGG AUC GUG CUG UCA GCU CAC GAG AGC UUC AGC CGG UUC GUU GAG GGG GUU GAG AUU
FN432840        AAC UGG AUC GUG CUG UCA GCU CAC GAG AGC UUC AGC CGG UUC GUU GAG GGG GUU GAG AUU
AJ608219        AAC UGG AUC GUG CUG UCA GCU CAC GAG AGC UUC AGC CGG UUC GUU GAG GGG GUU GAG AUU
AJ608214        AAC UGG ACC GUG CUG UCA GCU CAC GAG AGC UUC AGC CGG UUC GUU GAG GGG GUU GAG AUU
AJ608208        AAC UGG AUC GUG CUG UCA GCU CAC GAG AGC UUC AGC CGG UUC GUU GAG GGG GUU GAG AUU
AJ608209        AAC UGG ACC GUG CUG UCA GCU CAC GAG AGC UUC AGC CGG UUC GUU GAG GGG GUU GAG AUU
                **  *** * * * *  *  *** *** *** *** *** *** *** *** *** **  *** *** *** ***  **

                Frequent ORFx-frame stop codons downstream of ORFx; and nucleotide variability
                            reverts to primarily 3rd positions of ORF2a-frame codons
AJ608206        GAG CCC AUG UCC ACA CUA CGG UAU GGC AAG GUU CAG UCU GCC CCU AGG UUC GAU CCC UCU
L20893          GAG CCU AUG UCC AUG CUU CGG UAU GGC AAG GUU CAG UCU GCC CCU AGG UUC GAU CCC UCC
FN432838        GAG CCU AUG UCC ACA CUU CGG UAU GGC AAG GUU CAG UCU GCU CCC AGG UUU GAU CCC UCC
FN432837        GAG CCU AUG UCC ACG CUC CGA UAU GGU AAG GUU CAG UCU GCC CCU AGG UUC GAC CCC UCC
U23142          GAG CCC AUG UCC ACA CUC CGG UAU GGC AAG GUA CAA UCU GCC CCU AGG UUU GAU CCC UCC
AJ608212        GAG CCA AUG UCC ACA CUU CGG UAU GGC AAG GUG CAG UCU GCC CCC AGG UUC GAU CCC UCC
FN432841        GAG CCA AUG UCC ACA CUU CGG UAU GGC AAG GUG CAG UCU GCC CCC AGG UUC GAU CCC UCU
AJ608213        GAG CCA AUG UCC ACA CUU CGG UAU GGC AAG GUG CAG UCU GCC CCU AGG UUC GAU CCC UCC
AJ608210        GAG CCU AUG UCC ACG CUU CGG UAU GGG AAG GUU CAG UCU GCU CCC CGG UUU GAU CCC UCC
AM883054        GAG CCU AUG UCC ACG CUU CGG UAU GGG AAG GUU CAG UCU GCU CCC CGG UUU GAU CCC UCC
AJ608211        GAG CCU AUG UCC ACG CUU CGG UAU GGG AAG GUU CAG UCU GCU CCC CGG UUU GAU CCC UCC
AM883055        GAG CCU AUG UCC ACG CUU CGG UAU GGG AAG GUU CAG UCU GCU CCC CGG UUU GAU CCC UCC
AM883056        GAG CCU AUG UCC ACG CUU CGG UAU GGG AAG GUU CAG UCU GCU CCC CGG UUU GAU CCC UCC
AJ608215        GAG CCU UUG UCA ACA CUU CGG UAU GGU AAG GUU CAA UCU GCC CCG AGG UUC GAU CCC UCC
AM883057        GAA CCC UUG UCA ACA CUU CGG UAC GGC AAG GUU CAG UCC GCU CCA CGG UUC GAU CCC UCC
FN432839        GAA CCU AUG UCA ACA CUU CGG UAU GGC AAG GUU CAA UCC GCU CCA CGG UUC GAU CCC UCC
AJ876793        GAG CCU UUG UCC ACA CUU CGG UAU GGU AAG GUU CAA UCC GCU CCA AGG UUC GAU CCC UCC
AJ877020        GAG CCC UUG UCA ACA CUU CGG UAU GGC AAG GUU CAA UCU GCC CCA AGG UUU GAU CCC UCC
AM883058        GAA CCC UUG UCA ACA CUU CGG UAC GGC AAG GUU CAG UCC GCC CCA CGG UUU GAU CCC UCC
AJ608217        GAG CCC AUG UCU ACA CUU CGG UAU GGC AAG GUU CAG UCC GCC CCU CGG UUU GAU CCC UCC
AJ608218        GAG CCC AUG UCC ACA CUU CGG UAU GGC AAG GUU CAG UCC GCC CCC AGG UUC GAU CCC UCC
AJ608216        GAG CCC UUG UCC ACA CUC CGG UAU GGC AAG GUU CAG UCU GCC CCU AGG UUC GAU CCC UCU
AJ608207        GAG CCC AUG UCC ACA CUU CGG UAU GGC AAG GUU CAG UCU GCC CCU AGA UUC GAU CCC UCC
FN432840        GAG CCC AUG UCC ACA CUU CGG UAU GGC AAG GUU CAG UCU GCC CCU AGA UUC GAU CCC UCC
AJ608219        GAG CCC AUG UCC ACA CUU CGG UAU GGC AAG GUU CAG UCU GCC CCC AGG UUC GAU CCC UCC
AJ608214        GAG CCC AUG UCC ACA CUU CGG UAU GGC AAG GUU CAA UCU GCU CCC AGG UUC GAU CCC UCC
AJ608208        GAG CCC AUG UCC ACA CUA CGG UAU GGC AAG GUU CAG UCU GCC CCU AGG UUU GAU CCC UCC
AJ608209        GAG CCC AUG UCC ACC CUU CGG UAU GGC AAG GUU CAA UCU GCC CCC AGG UUC GAU CCC UCC
                **  **   ** **  *   **  **  **  **  *** **  **  **  **  **   *  **  **  *** **
                  ^   ^ ^     ^  ^^   ^   ^   ^   ^       ^   ^   ^   ^   ^ ^ ^   ^   ^       ^
```

Subterranean clover mottle virus

```
                 5'UTR
AF208001        -AC AAA AUC GCU CGA AAA AGA AAG CUU GAA UCC GUU UCG CUU UAU UCA AUU GAU AGA CGA
AY376454        -AC AAA AUC GCU CGA AGA AGA AAG CUU GAA UCC GUU UCG CUU UAU UCA AUU GAU AGA CGA
AY376452        -AC AAA AUC GCU CGA AGA AGA GAG CUU GAA UCC GUU UCG CUU UAU UCA AUU GAU AGA CGA
AY376451        -AC AAA AUC GCU CGA AGA AGA AAG CUU GAA UCC GUU UCG CUU UAU UCA AUU GAU AGA CGA
AY376453        -AC AAA AUC GCU CGA AGA AGA AAG CUU GAA UCC GUU UCG CUU UAU UCA AUU GAU AGA CGA
                 ** *** *** *** *** * * ***  ** *** *** *** *** *** *** *** *** *** *** ***

                            ORF1 (AUG1; poor context)
AF208001        UCA UUU UAU AUG CCA UCA GUC UCA AUU GAA GUU UAC AGU AGG GAG AGA CCA ACU UUA UUG
AY376454        UCA UUU UAU AUG CCA UCA GUC UCA AUU GAA GUU UAC AGU AGG GAG AGA CCA ACU UUA UUG
AY376452        UCA UUU UAU AUG CCA UCA GUC UCA AUU GAA GUU UAC AGU AGG GAG AGA CCA ACU UUA UUG
AY376451        UCA UUU UAU AUG CCA UCA GUC UCA AUU GAA GUU UAC AGU AAG GAG AGA CCA ACU UUA UUG
AY376453        UCA UUU CAU AUG CCA UCA GUC UCA AUU GAA GUU UAC AGU AAG GAG AGA CCA ACU UUA UUG
                *** ***  ** *** *** *** *** *** *** *** *** *** *** * * *** *** *** *** *** ***

AF208001        UUG UUG ACU CAC CGC UUC UGG CCU UCG AGC GAA ACA AUU CCG AGA GAU UAC GAA UAC GAC
AY376454        UUG UUG ACU CAC CGC UUC UGG CCU UCG AGC GAA ACA AUU CCG AGA GAU UAC GAA UAC GAC
AY376452        UUG UUG ACU CAC CGU UUC UGG CCU UCG AGU GAA ACA AUU CCG AGA GAU UAC GAA UAC GAU
AY376451        UUG UUG ACU CAC CGU UUC UGG CCU UCG AGU GAA ACA AUU CCG AGA GAU UAC GAA UAC GAC
AY376453        UUG UUG ACU CAC CGU UUC UGG CCU UCG AGU GAA ACA AUU CCG AGA GAU UAC GAA UAC GAC
                *** *** *** *** **  *** *** *** *** **  *** *** *** *** *** *** *** *** *** **
```

```
AF208001        CAA ACA GUC ACA CUG UAC GGU CGU UUC AAC AAC GAG GAG GAG ACU CUC AUC CUU UGC AUA
AY376454        CAA ACA GUC ACA CUG UAC GGU CGU UUC AAC AAC GAG GAG GAG ACU AUC AUC CUU UGC AUA
AY376452        CAA ACA GUC ACA CUG UGC GGU CGU UUC AAC AAC GAG GAG GAG ACU CAA AUU CUU UGC AUA
AY376451        CAA ACA GUC AUA CUA CAC GGU CGU UUC AAC AAC GAG GAG GAG ACU CAU AUC CUU UGC AUA
AY376453        CAA ACA GUC AUA CUA CAC GGU CGU UUC AAC AAC GAG GAG GAG ACU CAU AUC CUU UGC AUA
                *** *** *** * * **    * *** *** *** *** *** *** *** *** ***     **  *** *** ***

AF208001        UCA CGU UGC AAU ACC UGU GAC UUC UGG UGU GUC UUA GGA GAU UCA AAC CUU CCA GGC AUC
AY376454        UCA CGU UGC AAU ACC UGU GAC UUC UGG UGU GUC UUA GGA GAU UCA AAC CUU CCA GGC AUC
AY376452        UCA CGU UGC AAU ACU UGC GAC UUC UGG UGU GUC UUA GGA GAU CCA AAC CUU CCA GGC AUC
AY376451        UCA CGU UGC AGU AUC UGU GAC UUC UGG UGU GUC UUA GGA GAU UCA AAC CUU CCA GGC AUC
AY376453        UCA CGU UGC AGU AUC UGU GAC UUC UGG UGU GUC UUA GGA GAU UCA AAC CUU CCA GGC AUC
                *** *** *** * * *   **  *** *** *** *** *** *** *** ***  ** *** *** *** *** ***

AF208001        GAG AUU UGG GAU AGC GCA CUU AGU UGU UUC GCU AAC GCC GAA GAC UGU ACU CUU AGU GGA
AY376454        GAG AUU UGG GAU AGC GCA CUU AGU UGU UUC GCU AAC GCC GAA GAC UGU ACU CUU AGU GGA
AY376452        GAG AUU UGG GAC AGC GCA CUU AGU UGU UUC GCU AAC GCU GAA GAC UGC ACC CUU AGU GGA
AY376451        GAG AUU UGG GAC AGC GCA CUU AGU UGU UUC GCU AAC GCC GAA GAC UGU ACC CUU AGC GGA
AY376453        GAG AUU UGG GAC AGC GCA CUU AGU UGU UUC GCU AAC GCC GAA GAC UGU ACC CUU AGC GGA
                *** *** *** **  *** *** *** *** *** *** *** *** **  *** *** **  **  *** **  ***

AF208001        CAG UGC UCU GCC UGU AGA AAC CCU CCA GUU GAA GAA GAC GCG GAU UCC GAC UCA AGU GAG
AY376454        CAG UGC UCU GCC UGU AGA AAC CCU CCA GUU GAA GAA GAC GCG GAU UCC GAC UCA AGU GAG
AY376452        CAG UGC UCU GCC UGU AGA AAC CCU CCA GUU GAA GAA GAC GCG GAU UCC GAC UCA AGC GAG
AY376451        CAG UGC UCU GCC UGU AGA AAC CCU CCA GUU GAA GAA GAC GCG GAU UCC GAC UCA AGU GAG
AY376453        CAG UGC UCU GCC UGU AGA AAC CCU CCA GUU GAA GAA GAC GCG GAU UCC GAC UCA AGU GAG
                *** *** *** *** *** *** *** *** *** *** *** *** *** *** *** *** *** *** **  ***

                                                 Last upstream       Likely ORFx start
                                                 ORFx-frame stop     (GUG; strong context)
AF208001        GAA UAC CAA AGC UUA GGA AAC CGC ACC GUG AUC AGA GAC AGU GGC CCA GAA GGC UAC UGG
AY376454        GAA UAC CAA AGC UUA GGA AAC CGC ACC GUG AUC AGA GAC AGU GGC CCA GAA GGC UAC UGG
AY376452        GAA UAC CAA AGC UUA GGA AAC CGC ACC GUA AUC AGA GAC AGU GGC CCA GAA GGC UAC UGG
AY376451        GAA UAC CAA AGC UUA GGA AAC CGC ACC GUA AUC AGA GAC AGU GGC CCA GAA GGU UAC UGG
AY376453        GAA UAC CAA AGC UUA GGA AAC CGC ACC GUA AUC AGA GAC AGU GGC CCA GAA GGU UAC UGG
                *** *** *** *** *** *** *** *** *** **  *** *** *** *** *** *** *** **  *** ***

                                     Additional potential ORFx start sites
AF208001        UCG GAA UCU UCA GAC GAC GAA GAC CAC ACA CGA GCU GCA UAU AGU CCC UGC GAC UAC GAA
AY376454        UCG GAA UCU UCA GAC GAC GAA GAC CAC ACA CGA GCU GCA UAU AGU CCC UGC GAC UAC GAA
AY376452        UCG GAA UCU UCA GAC GAC GAA GAC UAC ACA CGA GCU GCA UAU AGU CCC UGC GAC UAC GAA
AY376451        UCG GAA UCU UCA GAC GAC GAA GAC UAC ACA CGA GCU GCA UAU AGU CCC UGC GAC UAC GAA
AY376453        UCG GAA UCU UCA GAC GAC GAA GAC UAC ACA CGA GCU GCA UAU AGU CCC UGC GAC UAC GAA
                *** *** *** *** *** *** *** ***  ** *** *** *** *** *** *** *** *** *** *** ***

AF208001        GGU AAC CUC GUA CGG UUG UUU CAA CUA CAA AGC AUU CAG CCU CUU CAA GAA GGA GAC GAU
AY376454        GGU AAC CUC GUA CGG UUG UUU CAA CUA CAA AGC AUU CAG CCU CUU CAA GAA GGA GAC GAU
AY376452        GGU AAC CUC GUA CGG UUG UUU CAA CUA CAA AGC AUU CAG CCU CUU CGA GAA AGA GAC GAU
AY376451        GGU AAC CUC GUA CGG UUG UUU CAA CUA CAA AGC AUU CAG CCU CUU CAA GAA GGA GAC GAU
AY376453        GGU AAC CUC GUA CGG UUG UUU CAA CUA CAA AGC AUU CAG CCU CUU CAA GAA GGA GAC GAU
                *** *** *** *** *** *** *** *** *** *** *** *** *** *** *** * * ***  ** *** ***

                         ORF1 stop
                        ORF2a (AUG2; strong context)
AF208001        CAA GA- AUG AAU ACC GAC ACU AUU CGA GCA AUC UUC CUA UAC UGG AUG AGC CUA GCU UUG
AY376454        CAA GA- AUG AAU ACC GAC ACU AUU CGA GCA AUC UUC CUA UAC UGG AUG AGC CUA GCU UUG
AY376452        CAA GA- AUG AAU ACC GAC ACU AUU CGA GCA AUC UUC CUA UAC UGG AUA AGC CUA GCU UUG
AY376451        CAA GA- AUG AAU ACC GAC ACU AUU CGA GCA AUC UUC CUA UAC UGG AUA AGC CUA GCC UUG
AY376453        CAA GA- AUG AAU ACC GAC ACU AUU CGA GCA AUC UUC CUA UAC UGG AUA AGC CUA GCC UUG
                *** **  *** *** *** *** *** *** *** *** *** *** *** *** *** **  *** *** **  ***

AF208001        AGC AUG GCG GCG GUG GUC CAA CUG GAA ACA AAG GUG GAC UUA UCA AAC CAC CAG UCG UUA
AY376454        AGC AUG GCG GCG GUG GUC CAA CUG GAA ACA AAG GUG GAC UUA UCA AAC CAC CAG UCG UUA
AY376452        AGC AUG GCG GUG GUG GUC CAA UUG GAA ACA AAG GUG GAC UUA UCG AAC CAC CAG UCG UUA
AY376451        AGC AUG GCG GCG GUG GUC CAA UUG GAA ACA AAG GUG GAU UUA UCG AAC CAC CAG UCA UUA
AY376453        AGC AUG GCG GCG GUG GUC CAA UUG GAA ACA AAG GUG GAU UUA UCG AAC CAC CAG UCA UUA
                *** *** *** * * *** *** ***  ** *** *** *** *** **  *** **  *** *** *** **  ***

AF208001        GUU AUG ACG ACA GCC AUC UUG CUG CCC GCA CUG AGC GUG CUG UUC GGC UUG CGC GUC UGG
AY376454        GUU AUG ACG ACA GCC AUC UUG CUG CCC GCA CUG AGC GUG CUG UUC GGC UUG CGC GUC UGG
AY376452        GUU AUG ACG ACA GCC AUC UUG CUG CCC GCA CUG AGC GUG CUG UUC GGC UUG CGC GUC UGG
AY376451        GUU AUG AUG AUA GCC AUC UUG CUG CCC GCA CUG AGC GUG CUG UUC GGC UUG CGC GUC UGG
AY376453        GUU AUG AUG AUA GCC AUC UUG CUG CCC GCA CUG AGC GUG CUG UUC GGC UUG CGC GUC UGG
```

```
                *** *** * * * * *** *** *** *** *** *** *** *** *** *** *** *** *** *** *** ***

AF208001        CGA AGA UGG AAG GUU GUG AUA GUG AAG GAG GUG AAC CCG CGA GAG GUG AUA AAC UUA GUG
AY376454        CGA AGA UGG AAG GUU GUG AUA GUG AAG GAG GUG AAC CCG CGA GAG GUG AUA AAC UUA GUG
AY376452        CGA AGA UGG AAG GUU GUG AUA GUG AAG GAG GCA AAC CCG CGA GAG GUG AUA AAC UUA GUG
AY376451        CGA AGA UGG AAG GUU GUG AUA GUG AAG GAG GUA AAC CCG CGA GAG GUG AUA AAC UUA GUG
AY376453        CGA AGA UGG AAG GUU GUG AUA GUG AAG GAG GUA AAC CCG CGA GAG GUG AUA AAC UUA GUG
                *** *** *** *** *** *** *** *** *** *** *   *** *** *** *** *** *** *** *** ***

                                  ORFx stop
AF208001        GGG GAG CCU UUC CUU GAU CCU GUG CGA GGU GUG UUG AUG AAU GGG AUC UCC ACU UCA GGA
AY376454        GGG GAG CCU UUC CUU GAU CCU GUG CGA GGU GUG UUG AUG AAU GGG AUC UCC ACU UCA GGA
AY376452        GGG GAG CCU UUC CUU GAU CCU GUG CGA GGU GUG UUG AUG AAU GGG AUC UCC ACU UCA GGA
AY376451        GGG GAG CCU UUC CUU GAU CCU GUG CGA GGU GUG UUG AUG AAU GGG AUC UCC ACU UCA GGA
AY376453        GGG GAG CCU UUC CUU GAU CCU GUG CGA GGU GUG UUG AUG AAU GGG AUC UCC ACU UCA GGA
                *** *** *** *** *** *** *** *** *** *** *** *** *** *** *** *** *** *** *** ***
```

Cocksfoot mottle virus

```
                  5'UTR
AB040447        --G AUA AUA GUG CGA AGA AAG ACA CAC UGU UAU CGU UUC CCC UCC CGA AUC AGA GGU UGA
L40905          --N AUA AUA GUG CGA AGA AAG ACA CAC UGU UAU CGU U-C CCC UCC CGA AUC AGA GGU UGA
DQ680848        --G AUA AUA GUG CGA AGA AAG ACA CAC UGU UAU CGU U-C CCC UCC CGA AUC AGA GGU UGA
Z48630          --G AUA AUA GUG CGA AGA AAG ACA CAC UGU UAU CGU U-C CCC UCC CGA AUC AGA GGU UGA
FJ669143        --G AUA AUA GUG CGA AGA AAG ACA CAC UGU UAU CAA U-C CCC UCC CAA AUC AGA GGU UGA
                    *** *** *** *** *** *** *** *** *** *** *   * * *** *** * * *** *** *** ***

                                ORF1 (AUG1; poor context)
AB040447        GAA GUA GCU UAG AUG UGC GAA CCU CCC CCU GGU UUU AUA ACC GUU CAG UGC UAC ACU AGC
L40905          GAA GUA GCU UAG AUG UGC GAA CCU CCC CCU GGU UUU AUA ACC GUU CAG UGC UAC ACC AGC
DQ680848        GAA GUA GCU UAG AUG UGC GAA CCU CCC CCU GGU UUU AUA ACC GUU CAG UGC UAC ACG AGC
Z48630          GAA GUA GCU UAG AUG UGC GAA CCU CCC CCU GGU UUU AUA ACC GUU CAG UGC UAC ACG AGC
FJ669143        GUA GUA GCU UAG AUG UGC GAA CCU CCC CCU GGU UUC AUA ACC AUA CAG UGU UAC ACG AGC
                * * *** *** *** *** *** *** *** *** *** *** **  *** ***  *  *** **  *** **  ***

AB040447        GAC GAU UUG UUG ACA GGC GAU UCU ACG AUC GUC AAG UCC AUU CCC GUC CGC UCG UGC UUC
L40905          GAC GAU UUG UUG ACA GGC GAU UCU ACA AUC GUC AAG UCC AUU CCC GUC CGC UCG UGC UUC
DQ680848        GAC GAU UUG UUG ACA GGC GAU UCC ACA AUU GUC AAG UCC AUU CCC GUC CGC UCG UGC UUC
Z48630          GAC GAU UUG UUG ACA GGC GAU UCC ACA AUU GUC AAG UCC AUU CCC GUC CGC UCG UGC UUC
FJ669143        GAC GAU UUG UCG ACA GGC GAU UCA ACA AUC GUC AAG UCC AUC CCC GUG CGC UCG UGC UUC
                *** *** *** * * *** *** *** **  **  **  *** *** *** **  *** **  *** *** *** ***

AB040447        UUC CGC CAA GGC GUC GAA GUU GUU CUG UUC CGG UGU GAG UCU AAC AAA CAU CGC UGG UCG
L40905          UUC CGC CAA GGC GUU GAA GUU GUU CUG UUC CGG UGU GAG UCU AAC AGA CAU CGC UGG UCG
DQ680848        UUC CGC CAA GGC GUU GAA GUU GUU CUG UUC CGG UGU GAG UCC AAC AAA CAU CGC UGG UUG
Z48630          UUC CGC CAA GGC GUU GAA GUU GUU CUG UUC CGG UGU GAG UCC AAC AAA CAU CGC UGG UCG
FJ669143        UUC CGC CAA GGC GUU GAA GUU GUU CUG UUU CGG UGU GAG UCC AAC AAG CAU CGC UGG UCG
                *** *** *** *** **  *** *** *** *** **  *** *** *** **  *** *   *** *** *** * *

AB040447        AAG AUC AGA GGU CCU GUG AAC UUA ACA GUU CAU UGU GAU AUC UGU GAA UUC CGC GAG ACU
L40905          AAG AUC AGA GGU CCU GUG AAC UUG ACA GUU CAU UGU GAU AUC UGU GAA UUC CGC GAA ACU
DQ680848        AAG AUC AGA GGU CCU GUG AGC UUG ACA GUU CAC UGU GAU AUC UGU GAA UUC CGC GAG ACC
Z48630          AAG AUC AGA GGU CCU GUG AGC UUG ACA GUU CAC UGU GAU AUC UGU GAA UUC CGC GAA ACU
FJ669143        AAG AUC AGA GGU CCG GUG AGC CUU ACA GUU CAU UGU GAU AUC UGU GAA UUC CGC GAG ACA
                *** *** *** *** **  *** * *  *  *** *** **  *** *** *** *** *** *** *** **  **

                                                                                   Last upstream
                                                                                 ORFx-frame stop
AB040447        GUU GAG AUU CCA UCU CUG CCC AAA GGC UUU AAA GUA UCG AGC GAU UUC UCU UAC AGU GUA
L40905          GUU GAG AUU CCA UCC CUG CCA AAA GGC UUU AAA GUA UCA AGC GAU UUC UCU UAC AGU GUA
DQ680848        GUU GAG AUU CCA UCC CUG CCC AAA GGC UUC AAA GUA UCA AGC GAU UUC UCU UAC AGU GUA
Z48630          GUU GUG AUU CCA UCC CUG CCC AAA GGC UUC AAA GUA UCU AGC GAU UUC UCU UAC AGU GUA
FJ669143        GUU GAG AUU CCA UCU CUG CCC AAA GGC UUC AAA GUA GCU AGC GAU UUC UCU UAC AGU GUA
                *** * * *** *** **  *** **  *** *** **  *** ***  *  *** *** *** *** *** *** ***

                         Likely ORFx starts (AUU and GUG
                         with G at -3 and G at +4; monocot)     ORF1 stop
AB040447        ACG UGG AAU UGC UGC UAC AGC CGU GGC AGG ACA GAG UAG -CA CAG AGG UUA GUU GGU UGA
L40905          ACG UGG AAU UGC UGC UAC AGC CGU GGC AGG ACA GAG UAG -CA CCG AGG UUA GUU GGU UGA
DQ680848        ACG UGG AAU UGC UGC UAC AGC CGU GGC AGG ACA GAG UAG -CA CCG AGG UUA GUU GGU UGA
Z48630          ACG UGG AAU UGC UGU UAC AGC CGU GGC AGG ACA GAG UAG -CA CAG AGG UUA GUU GGU UGA
FJ669143        ACG UGG AAU UGC UGU UAC AGC CGU GGC AGG ACA GAG UAG -CA CUG AGG UUA GUU GGU UGA
```

```
                *** *** *** *** **  *** *** *** *** *** *** *** ***  ** * * *** *** *** *** ***

                                ORF2a (AUG2; strong context)
AB040447        GGG UGA CCA AGA AUG GGU UGU UCU GUU GUU GGA AAC UGC AAG UCC GUG AUG UUG AUG AGC
L40905          GGG UGA CCA AGA AUG GGU UGU UCU GUU GUU GGA AAC UGC AAG UCC GUG AUG UUG AUG AGC
DQ680848        GGG UGA CCA AGA AUG GGU UGU UCU GUU GUU GGA AAC UGC AAG UCC GUG AUG UUG AUG AGC
Z48630          GGG UGA CCA AGA AUG GGU UGU UCU GUU GUU GGA AAC UGC AAG UCC GUG AUG UUG AUG AGC
FJ669143        GGG UGA CCA AGA AUG GGU UGU UCU GUU GUU GGA AAC UGC AAG UCC GUG AUG UCG AUG AGC
                *** *** *** *** *** *** *** *** *** *** *** *** *** *** *** *** *** * * *** ***

AB040447        AGG AUG AGC UGG UCA AAG CUA GCU CUC CUC AUA UCC GUC GCA AUG GCG GCA GCC AUG ACA
L40905          AGG AUG AGU UGG UCA AAG CUA GCU CUC CUC GUA UCC GUC GCA AUG GCG GCA GCC AUG ACA
DQ680848        AGG AUG AGC UGG UCA AAG CUA GCU CUC CUC AUA UCC GUC GCA AUG GCG GCA GCC AUG ACA
Z48630          AGG AUG AGC UGG UCA AAG CUA GCU CUC CUC GUA UCC GUC GCA AUG GCG GCA GCC AUG ACA
FJ669143        AGG AUG AGC GGG CCA AAG CUA GCU CUC CUC AUA UCC GUC GCA AUG GCG GCA GCC AUG ACA
                *** *** **   **  ** *** *** *** *** ***  ** *** *** *** *** *** *** *** *** ***

AB040447        GAC UCC CCA CCC ACU CUG AUA UGU AUG GGG AUC CUA GUG AGC GUG GUG CUC AAC UGG AUC
L40905          GAC UCC CCA CCC ACU CUG AUA UGU AUG GGG AUC CUA GUG AGC GUG GUG CUC AAC UGG AUC
DQ680848        GAC UCC CCA CCC ACU CUG AUA UGU AUG GGG AUC CUA GUG AGC GUG GUG CUC AAC UGG AUC
Z48630          GAC UCC CCA CCC ACU CUG AUA UGU AUG GGG AUC CUA GUG AGC GUG GUG CUC AAC UGG AUC
FJ669143        GAC UCC CCA CCC ACU CUG AUA UGU AUG GGG AUC CUA GUG AGC GUG GUG CUC AAC UGG AUC
                *** *** *** *** *** *** *** *** *** *** *** *** *** *** *** *** *** *** *** ***

AB040447        GUC UGC GCA GUA UGC GAA GAA GCU UCA GAG CUA AUU CUG GGC GUU UCC UUG GAA GCG ACU
L40905          GUU UGC GCA GUA UGC GAA GAA GCU UCC GAG CUA AUU CUG GGG GUU UCC UUG GAA GCG ACU
DQ680848        GUU UGC GCA GUA UGC GAA GAA GCU UCA GAG CUA AUU CUG GGC GUU UCC UUG GAA ACG ACU
Z48630          GUC UGC GCA GUA UGG CAA GAA GCU UCA GAG CUA AUU CUG GGC GUU UCC UUG GAA GCG ACU
FJ669143        GUC UGC GCA GUA UGC GAA GAA GCU UCA GAG CUA AUU CUG GGC GUU ACG UUA GAA GCG ACU
                **  *** *** *** **   ** *** *** **  *** *** *** *** **  ***  *  **  ***  ** ***

                      ORFx stop
AB040447        CGU CCU AGU CCC GCA CGG GUU AUA GGA GAG CCG GUC UUU GAC CCC CGG UAC GGC UAU GUC
L40905          CGU CCU AGU CCC GGA CGG GUU AUA GGA GAG CCG GUC UUU GAC CCC CGA UAU GGC UAU GUC
DQ680848        CGU CCU AGU CCC GCA CGG GUA AUA GGA GAG CCG GUC UUU GAU CCC CGG UAU GGC UAU GUC
Z48630          CGU CCU AGU CCC GCA CGG GUC AUA GGA GAG CCG GUC UUU GAC CCC CGG UAU GGC UAU GUU
FJ669143        CGU CCU AGC CCC GCA AGG GUU AUA GGA GAG CCA GUU UUC GAC CCC CGG UAU GGU UAU GUC
                *** *** **  *** * *  ** **  *** *** *** **  **  **  **  *** **  **  **  *** **
```

Southern bean mosaic, sesbania mosaic, soybean yellow common mosaic and southern cowpea mosaic viruses

```
                  5'UTR                                                             ORF1     sORF
AF055887        --C ACA AAA UAU AAG AAG GAA A-- -GC UGG AUU UCC -UA CCU UUG UGU UUC C-- AUU GUC
AF055888        --C ACA AAA UAU AAG AAG GAA A-- -GC UGG AUU UCC -UA CCU UUG UGU UUC C-- AUU GUC
DQ875594        --C ACA AAA UAU AAG AAG GAA A-- -GC UGG AUU UCC -UA CCU UUG UGU UUC C-- AUU GUC
AY004291        --C ACA AAA UAU AAG AAG GAA A-- -GC UGG AUU UCC -UA CCU UUG UGU UUC C-- AUU GUC
JF495127        --- ACA AAA UAU AAG AAC AAA GAG -UC UGG AUC UUU GUA CCU UUG UGC CAC C-- --U AAG
M23021          --C ACA AAA UAU AAG AAG GAA AAG UGC UGA UUU UCC -UA CCU UUG UGU UUC AUG AUU UAU
                    *** *** *** *** **   **       * **   *  *    ** *** *** **    *       *

                                                                                            ORF1
AF055887        GAA GCA UUG GUC --- --- --- --- --- --- --- --- --- --A ACG AUU ACA AAA CGG UGC
AF055888        GAA GCA UUG GUC --- --- --- --- --- --- --- --- --- --A ACG AUU ACA AAA CGG UGC
DQ875594        GAA GCA UUG GUC --- --- --- --- --- --- --- --- --- --A AUA CUU AUC AAU UGG UGC
AY004291        GAA GCA UUG GUC --- --- --- --- --- --- --- --- --- --- --- AAA CCC UAU UUG AUG
JF495127        CUG GUA UUG GUU --- --- --- --- --- --- --- --- --- --- --U AUC ACA CAA CGU AUG
M23021          GAG ACA UUG GUU UUA AGC AAA ACU GAG UUA GAG CAA CUC AAC GUC GAU UUA CCU CGA UAU
                      * *** **

                            ORF1 (SBMV)
AF055887        AUU UUC UGC AUG AGC UAU CGU UUC CUA ACA GUU AGA GCA UUC GGC UUU ACC GGU UUU CAU
AF055888        AUU UUC UGC AUG AGC UAU CGU UUC CUA ACA GUU AGA GCA UUC GGC UUU ACC GGU UUC CAU
DQ875594        AUU GUU CGC AUG AGC UAC CGA UUC UUA GUA GUC AAA GCC GUU GGU UUU CUU GGU UUC CAU
AY004291        CAA GCU CAG CAU ACU UUC ACC AUC AAA UUU CUG AGA CAC GCG UGC UUC AUU GGU UUU GAG
JF495127        ACC GAC ACA GUC GUC GUG AAC UUU UCA CUG AUC CGA ACC CGG UGC GUG GAA CGC UUU GGC
M23021          UCC UAC AGG UUU CGU UAC AGU CGU UCG AUU GGC GAU ACU GUA GUA GAG UUU CCU GGU ACU

AF055887        UGC GAC GCC ACG CGC UUG CUA AGU GAA ACA GAA GUC AUA GAC --- GUU CCA UCG UCU CUU
AF055888        UGC GAC GCC ACG CGC UUG CUA AGU GAA ACA GAA GUC AUA GAC --- GUU CCA UCG UCU CUU
DQ875594        UCA GAC GCU ACU CGC AUU CUG UCA GAG ACU GAG AUC GUA GAC --- GUU CCU UCG UCC AUU
AY004291        GAC CCU AGA GUU GUU CUU GAC CAC GAA GAG AUC GAU AUU CCC --- UGU GAA GUG GAA UUU
```

```
JF495127        GUG AAU UCC CUC CCA GUU GGA AGA CAU UUC AUU GGU UCU GAG GAA AUU CCU ACC GGC AUC
M23021          CUC UCG GAC CCC UGU AUC CCU GUC GUU GAC GUA CUC CUU GGA GCU UGU UGG GCU UGG CCU
                                     *                                        *
                                                         sORF
AF055887        GAU UUU GUU GGU GAA ACC GAA CUC AGA CUU GAA ACU GCU UGG CCC CAG UGU GAA GAG AAU
AF055888        GAU UUU GUU GGU GAA ACC GAA CUC AGA CUU GAA ACU GCU UGG CCC CAG UGU GAA GAG AAU
DQ875594        GAU UUC GUC GGU GAA ACC GAG UUA CGC CUA GAA AAC GCU UGG CCC CAA GGU GGU GAG ---
AY004291        GAU UGC AAC GCU GAG UCC GUU UGU UUG GUG AGA GCC CAC AAC CAA CCG UAU AGC GAA GGA
JF495127        GAU UUC GUU GAG CGG UCU UUU CAG GUU ACG GCC UUU GGG AAU UAU UCG GUU CCU GAG AGA
M23021          CAA CCU UCA CGA CAC GGU GGU UUG GGG UUA GAU GAU AUC GAC CCU UUU GAC GCU AGC UUC
                 *

AF055887        UGU UAC ACG AUU CUC CCU CGA UUC AAC GUU CAA GUU GAU UUC GAG UAU UAC --- --- ---
AF055888        UGU UAC ACG AUU CUC CCU CGA UUC AAC GUU CAA GUU GAU UUC GAG UAU UAC --- --- ---
DQ875594        AGA UAC ACU AUC CUA CCU AGG UUC AAC GUU CAG AUU GAC UUC ACG UAC CAU --- --- ---
AY004291        GAA GUU AAG AAC UAU UCU GUU UAC UUU AAC AGU GGU CUU AAC GAC UAC UAC GGC CCA CUA
JF495127        AGC AGA GUU AGA UUG AUA GAC UGC CCG GUG UCG UGC GAG GGG ACU UUU AAG UUC GAC CCU
M23021          UCG UGC UGC GUG ACC UCU CCG GAA AGG UAC UGC CUA UCA AGG UCU GUA UUA AGC GGA GUU

AF055887        --- CCU GUG CGU GUC GAG AUU GUG UGC CGA GUC UGC GCU GCA UCC CUA UCU GUC AUC UUC
AF055888        --- CCU GUG CGU GUC GAG AUU GUG UGC CGA GUC UGC GCU GCA UCC CUA UCU GUC AUC UUC
DQ875594        --- CCA GUG CGU GUC GAG AUC AUC UGU AGG GUU UGU GCU ACU UCC CUU ACU GUU GUC UUU
AY004291        CUC CCU GUU UGG UUA GAG AUU GUG UGC CGG GUG UGU GCU ACC UCU UAU UCC UUU GUC UUG
JF495127        GUA CCC CUG UGG CUA UCC AUC AAC UGC CGA GUU UGU CAA ACU GAG GCU AAC CUU AUC AUC
M23021          GAC GCG UUU GUU GUC CGC GGA UCC UGC AAG UUG UGU GGA UUA GGA UUU CUC GAC AAU UUC
                     *   *       *              **       *  **                               *

                                                                      Last upstream ORFx-
                                                                        frame stop (SBMV)
AF055887        AGC AAG UGG GAC UUC UAC UGU AGU --- --- AGG AGA GGC CAU UUU GUU CCU GUA GAU CAA
AF055888        AGC AAG UGG GAC UUC UAC UGU AGU --- --- AGG AGA GGC CAU UUU GUU CCU GUA GAU CAA
DQ875594        AGC AAG UGG AAC UUC CAU UGC GAA --- --- AGG AAG GGC CAU UUU GUG CCA GUA GAC CAG
AY004291        GCA CCA GAG GAU CUG GAA GAC GAG GCU GGU CGA GUU UCG AGG AAG UAC GCU CCU UGU GAA
JF495127        AAC CAG GAG UUG GUG CCU GGG UAU AGU --- GAA AAC AGC AAG UUC GUG UUU UGU GAC ACC
M23021          AAU CCU UUU GAA AUU CGU GCG UUG CUU GGU CAA ACC ACU CCU GGU UUG UGG UGG CAA CCC
                                 *

                 Likely ORFx
                 start (SBMV)
AF055887        AAC GGG GAU CUG UUU AGG AUU GGA ACG CUC CAG GAG ACG GGA --- --- --- GAG AAA UAC
AF055888        AAC GGG GAU CUG UUU AGG AUU GGA ACG CUC CAG GAG ACG GGA --- --- --- GAG AAA UAC
DQ875594        AAC GGG AAU CUG UUU AGG GUU GGA ACG CUC CGG GAG ACG GGA --- --- --- GAG AAA UAC
AY004291        AAC GGG AAA GUG UUU CAG CGG GCU GUG CGG CGC GCA ACA GGA --- --- --- GAG GAA UAC
JF495127        GAA GGG AGA CCU UUG CGA AUC AGA ACA CGA AGA GGC AUU CAG CCU --- --- AGC CGA UUU
M23021          GUC AAG CCA GUC UAC GAC GAC AGG AAC AUU CAC CUC CCG UAC GAC UCC GAG CUU AAC GCG
                      *         *

                                                            ORF2a
AF055887        UUC UAC UUC UGU GAU AAA UCU AUC UGU AGA CA- AUG UAU CAU CCA GGC CGC UCA CCA UCA
AF055888        UUC UAC UUC UGU GAU AAA UCU AUC UGU AGA CA- AUG UAU CAU CCA GGC CGC UCA CCA UCA
DQ875594        UUC UAC UUC UGU GAG AAA UCU AUC UGC AGA CA- AUG UAU CAU CCA GGC CGC UCA CCA UCA
AY004291        UUC UAU UCG UGU CGA AAG AAA GUU UGC CGA GA- AUG UAU CAU CCG AGC UGC AAG GGC UAU
JF495127        UAC CAC CAG GAA GAA AAG ACC AUU UGU CAG UC- AUG CGU CAU CCA GGG CUG UUU GCG UAC
M23021          AGA AUA CAA CGC UUC CAG UAC ACC UGC CGA GA- AUG UAU CGU CCG AGU UGC CUU UCA UAU
                                     *          **          ***   * * * **    *              *

                     ORF1 stop
AF055887        UUC CUG AUA ACG UUG GCA AAC GUU AUC UGC GCG GCG AUU CUG UUC GAC AUC CAU ACG GGG
AF055888        UUC CUG AUA ACG UUG GCA AAC GUU AUC UGC GCG GCG AUU CUG UUC GAC AUC CAU ACG GGG
DQ875594        UUC CUG AUA ACG UUA GCA AAU GUU AUC UGC GCG GCA AUC UUG UAC GAC AUC CGU AUG GGG
AY004291        GUC CUC CUC CUA GCC AAU AUG GCA AGC UUC GCG AUC CUU GUG GAC ACG UUC CUU AUG GGG
JF495127        ACG CUC UUG GUC GUG AAC AUG GCA AGC UUU GCU AUA CUC CUA GAU UCG UUC AUG CUG GGA
M23021          GUC CUC CUA GUU GCG AAC AUG UGG AGC UUC GCC GUG UGC GCA AAU GCG UUC AUC UAC GGG
                    **   *              *       * * *   **                       **         **

AF055887        GGU UAC CAA CCA GGC UCA CUA AUU CCA AUA GUG GCU UGG AUG ACC CCG UUC GUU ACU CUG
AF055888        GGU UAC CAA CCC GGC UCA CUA AUU CCA AUA GUG GCU UGG AUG ACC CCG UUC GUU ACU CUG
DQ875594        GGG UAC CAA CCA GGA UCA CUA GUU CCA AUA GUG GCC UGG AUG ACC CCG UUC GUU ACG CUG
AY004291        GUG CAC AAC CCC UCA CAC GUU GUC CCG GUG GUG GCA UUG AUG ACU UUG UGC GCU ACU CCG
JF495127        GCA UAC AGG UCA GAU CAC AUG GUU CCA GUU GUG GCC UUG AUG ACC CUG UGC GCU ACC GUG
M23021          UCA UAC GAC CCC AGC CAC AAU AUU CCA AUU GUG GCC UUG AUG ACC CUG UGC GCU ACA GGG
                     **      *               *  **   *  ***  **  * * *** **    * * * * * **    *
```

```
                                                              ORFx stop (SBMV)
AF055887        CUC UGG UUG AGC GCG UCA UUC GCG ACA UAC CUU UAU AAG UAU GUG AGA ACU CGG CUG CUC
AF055888        CUC UGG UUG AGC GCG UCA UUC GCG ACA UAC CUU UAU AAG UAU GUG AGA ACU CGG CUG CUC
DQ875594        CUC UGG UUG AGC GCG UCA UUC GUG ACA UAC CUU UAU AGG UAU GCU CGA ACU CGA CUG CUU
AY004291        CUU UGG UUG AGC GCC UCA UAC GUG AUU UAC CUG CUC AGG UAC AUG UUG GUG AGG UUG UCU
JF495127        CUU UGG UUG AGU CUG UCA CUC GUG AGC UUC CUU UAC GGA UAU GUC CGA GUU CGG CUA GUU
M23021          CUC UGG UUG AGC ACG UCC GUG GUG UCC UUC GGG AUU CGC UAC GUG AGG GUG CGA GUU UCG
                **  *** *** **      **      * *     * *             **               *   *

                  ORFx stop
AF055887        CCA GAG GAG AAG GUU GCC AGG GUC UAU UAU ACG GCG CAG UCU GCA CCG UAU UUU GAC CCU
AF055888        CCA GAG GAG AAG GUU GCC AGG GUC UAU UAU ACG GCG CAG UCU GCA CCG UAU UUU GAC CCU
DQ875594        CCA GAG GAG AAA GUG GCC AGA GUU UAU UAU ACG GCG CAA UCU GCG CCU UAC UUU GAC CCG
AY004291        CCU GAG GCA AAG CAG GAC AGA GUC UAC UAU ACC GCU ACU ACA GCA CCG UAU UUC GAU CCG
JF495127        CCC GAA GCU AAG CAG GAA CGG AAG UAU UAU GUA GCC CAU UCG GCU CCA UAU UUC GAU CCC
M23021          CCU GAA AAG ACG CAG AAC CGC ACC AUA UAC GUC UCC UCU GGG CUA CCU CAU UUU GAC CCC
                **  **      *            *          **       *              **   *  **  **  **
```

Sowbane mosaic and *Rubus* chlorotic mottle viruses

```
                                                  sORF (AUG1; following moderately efficient re-
                                                  initiation after translation of a short ORF, this
                 5'UTR                            is expected to shunt ribosomes past the ORF1 AUG)†
GQ845002        --C CAA AAU AUA AGA AC- GAA GUG CAA UGU UCG UGC CAU UUC CAG ACG UAU UGC AAA AUA
HM163159        -AC CAA AAU AUA AGA ACC GAA GUG UAA UGU UCG UGC CAU UUC CAG ACG UAU UGC AAA AUA
AM940437        --A CAA AAU AUA AGA AC- GAA GUA UUA UGU UCG UAC CAU UUC CAG ACG UAU UGC AAA AUA
                    *** *** *** *** **  *** **    * *** *** * * *** *** *** *** *** *** *** ***

                                            ORF1 (AUG2)       sORF stop
GQ845002        CAG CUG UUG GCU CUA AAG UAA AUG AAA UUC ACG CUU AAU CUC AGA AGC AUC GGA CCA AAC
HM163159        CAG CUG UUG GCU UUA AAG UAA AUG AAA UUC ACG CUU AAU CUC AGA AGC AUC GGA CCA AAC
AM940437        CAG CUG UUG GUU CUA AAG UAA AUG AAA UUC ACG CUU AAU CUC AGA AGC AUC GGA CCC AAC
                *** *** *** * *  ** *** *** *** *** *** *** *** *** *** *** *** *** *** **  ***

GQ845002        CAG AUC GAG ACA AGU AAG ACU UAC AUU CCC AGU UGC GAA UUC UUU CCU GAC UGU GAU UUC
HM163159        CAG AUU GAG ACA AGC AAG ACU UAC AUU UCC AGU UGU GAA UUC UUU CCU GAC UGU GAU UUC
AM940437        CAA AUU GAG GCU AAG AAG ACC UAC GUU UCU AGU UGU GAA GUC UUU CCC AAC UGU GAU UAC
                **  **  ***  *  *   *** **  ***  **  *  *** **  ***  ** *** **   ** *** *** * *

GQ845002        GAA GUC AAC UCU CUG GCU GCU UUA GUG CGU AAC GUC AAG UCC UUC GGA GUU AAG CAU UGG
HM163159        GAA GUC AAC UCU CUG GUU GCU CUA GUG CGU AAC GUU AAG UCC UUC GGA GUU AAG CAU UGG
AM940437        GAG GUC AAC UCU UUG GUU GCG CUU GUG CGG AAA GUU AAG UCG UUU GGA GUU AGA CAU UGG
                **  *** *** ***  ** * * **   *  *** **  **  **  *** **  **  *** *** *   *** ***

GQ845002        ACG GCA GGA CCC AGU GUG CGG UUG UGG AAU UUG UAC GAA AGU GCA UUC CAA GAC CAC GCC
HM163159        ACG GCA GGA CCC AGU GUG CGG UUG UGG AAU CUG UAC GAA AGU GCA UUC CAA GAC CAC GCU
AM940437        ACG GCA GGA CCC AGC GUG CGU UUG UGG AAU UUG UAC GAA AGC GAA UUC CCG GAC UGC GCG
                *** *** *** *** **  *** **  *** *** ***  ** *** *** **  * * *** *   ***   * **

GQ845002        AAG CAC CAC UAC AUU GCG UUU GAA GUG GUU UGC GCG UUC UGU GAC CAG GUG UUU CAG ACG
HM163159        AAG CAC CAC UAC AUC GCG UUU GAA GUG GUU UGC GCG UUC UGU GAC CAG GUG UUU CAG ACG
AM940437        AAG CAC CAC UAC AUC GCU UUU GAA GUG UGU UGC UCG UUC UGU GAC CAG GUG UUU CAG ACU
                *** *** *** *** **  **  *** *** ***   * ***  ** *** *** *** *** *** *** *** **

GQ845002        ACC CCA ACA CCG UCG AGU CAA AUC UCC GGU UGU UGG CCA GUG UUC GUU GAC GGA GUU AAC
HM163159        ACU CCA ACA CCG UCG AGU CAA AUC UCC GGU UGU UGG CCA GUG UUC GUU GAC GGA GUU AAC
AM940437        ACC CCC ACA CCG UCG AGC CAG AUC UCC GGU UGU UGG CCA GUA UUC GUU GAC GGA GUC AAC
                **  **  *** *** *** **  **  *** *** *** *** *** *** **  *** *** *** *** **  ***

GQ845002        AGA GGA AUU GUC ACG ACU GCG CGC CGA AAG GAA GCC GAA GUC GUA CAA GGC AAA CCC GAC
HM163159        AGA GGA AUU GUC ACG ACU GCG CGC CGA AAG GAA GCC GAA GUC GUA CAA GGU AAA CCC GAC
AM940437        AGA GGU AUU GUC ACG ACU GCG CGC CGA AAG GAA GCU AGC GUC GUA CAA GGU AAA CCC GAC
                *** **  *** *** *** *** *** *** *** *** *** **      *** *** *** **  *** *** ***

                                                                ORF2a (AUG3; strong context)
GQ845002        UGU UAC AUC GUC CGA UUU UCG UCC ACC UGU AAA GA- AUG CAU CGU UCA CCG UUU GGC UAC
HM163159        UGU UAC AUC GUC CGA UUU UCG UCC ACC UGU AAA GA- AUG CAU CGU UCA CCG UUU GGC UAC
AM940437        UGU UAC AUC GUC CGU UUC AAG UCU GCC UGC ACA GA- AUG CAU CGU UCA CCG UUU GGC UAC
                *** *** *** *** **  **    * **   ** **  * * **  *** *** *** *** *** *** *** ***

                                                             ORF1 stop
GQ845002        CUC CUA CUG CUC AUC AGC CUU CCA GCU GGA GCA GUG ACU UAC GAU UGC GUG CGA GGG GAC
```

```
HM163159        CUC CUA CUG CUC AUC AGC CUC CCA GCU GGA GCA GUG ACU UAC GAU UGC GUG CGA GGG GAC
AM940437        CUC CUG CUG CUC AUC AGC CUC CCA GCU GGA GCA GUG ACU UAC GAC UGC AUA AGA GGG GAC
                *** **  *** *** *** *** **  *** *** *** *** *** *** *** **  ***  *   ** *** ***

GQ845002        UGC CCA GCA GGA CCC AAC UGU CUG AUG AUG GCU CUG AUG ACU CUG UUC ACU ACA GGA AUG
HM163159        UGC CCA GCA GGA CCC AAC UGU CUG AUG AUG GCU CUG AUG ACU CUG CUC ACU ACA GGA AUG
AM940437        UGC CAA GCA GGA CCC AAC AGU UUG AUG AUG GCU CUG AUG ACU CUA CUC ACU ACA GGA AUG
                *** * * *** *** *** ***  **  ** *** *** *** *** *** *** **   ** *** *** *** ***

GQ845002        UGG UGG AUA GUC UCU CUA AGC CUA UAC UGG AUA GAG CGA CAG CGU UAU CGC UCA GCC GAA
HM163159        UGG GGG AUA GUC UCU CUA AGC CUA UAC UGG AUA GAG CGA CAG CGU UAU CGC UCA GCC GAA
AM940437        UGG UGG GUA GUC UCG CUA AGC CUA UUC UGG AUA GAG CGG CAG CGU UAU CGC UCA GCC GAA
                ***  **  ** *** **  *** *** *** * * *** *** *** **  *** *** *** *** *** *** ***

GQ845002        GAG AAG AUC UCA CGG CCG AAG CGA UUG CGG CUC AUU GGC GAU CCU UAC UUG GAU CCC UGU
HM163159        GAG AAG AUC UCA CGG CCG AAG CGA UUG CGG CUC AUU GGC GAU CCU UAC UUG GAU CCC UGU
AM940437        GAG AAG AUC UCA CGG CCG AAG CGA UUG CGG CUC AUU GGC GAU CCU UAU UUG GAU CCC UGU
                *** *** *** *** *** *** *** *** *** *** *** *** *** *** *** **  *** *** *** ***

                ORFx stop
GQ845002        GAU GGG AUC GUU GGG AAA AUU CUC GAU GAU UGC ACC
HM163159        GAU GGA AUC GUC GGG AAG AUC CUC GAU GAU UGC ACC
AM940437        GAU GGA AUU GUC GGG AAG AUU CUC GAC GAU UGC ACC
                *** **  **  **  *** **  **  *** **  *** *** ***
```

† The sORF in Sowbane mosaic and *Rubus* chlorotic mottle viruses may in fact encode a functional peptide (2 synonymous substitutions but only 1 non-synonymous substitution between the two species).

Turnip rosette virus

```
                 5'UTR
TRoV-1          -CA AAA UAA AUA CAA GAA AGA AAG AUU UUC UCC -CA CAG CUU GUA UUA UCU CUA CGA CAU
TRoV-2          -CA AAA UAA AUA CAA GAA AGA AAG AUU UUC UCC ACA UAC CUU GUA UUU UCU CUA CAA -AC
                 ** *** *** *** *** *** *** *** *** *** ***  **  *  *** *** **  *** *** * *  *

                  sORF1    ORF1   sORF1
                  (AUG1)   (AUG2) stop
TRoV-1          UAA UGA UUA AUG AGU AGA GUU GCC ACA AUC GAA AUA UAC AAC GAG AAC GGA AUA AUC GUA
TRoV-2          AUA UGA UUU AUG AGU AGA GUU GCC ACA GUC GAA AUA UAC AAC GAG AAC GGA AUA AUC GUA
                 *  *** **  *** *** *** *** *** ***  ** *** *** *** *** *** *** *** *** *** ***

                                                 sORF2
TRoV-1          GCU CGG AAG AAG ACG UCG GGA CCG CAC GCG CUC CUA GAA CUC UUC AAC GGA AAG CAG AAA
TRoV-2          GCU CAC AAG AAG ACG UCA GGA CCA CAU GCG CUC CUA GAA CUC UUC AAC GGU AAG CAG AAA
                *** *   *** *** *** **  *** **  **  *** *** *** *** *** *** *** **  *** *** ***

TRoV-1          UAC GAU CAG GUG UCC GAA CUC UUU GUA AUU UGG GUU UGU GAA GAG UGU GGG AAA ACC GUG
TRoV-2          UAC GAU CAG GUG UCC GAA CUC UUU GUA AUU UGG AGC UGU GAA GAG UGU GGG AAA ACC GUG
                *** *** *** *** *** *** *** *** *** *** ***     *** *** *** *** *** *** *** ***

TRoV-1          UAC UCU ACG UGC GAA UUU AAA GGA AUC GUA UUU GUU AGA GAG GAC GGG AAA GAG ACA ACU
TRoV-2          UAC UCC ACG UGC GAA UUC AAA GGA AUC GUA UUU GUU CGA GAA GAC GGA AAA GAG ACA ACU
                *** **  *** *** *** **  *** *** *** *** *** ***  ** **  *** **  *** *** *** ***

                                             Last upstream ORFx-
                                             frame stop (TRoV-2)
TRoV-1          GAA UUC GAA ACA GAA GCA GUU GUA GAC UCC GAC GAU UGU GGG UGU GCU UAC GAG UAU CAU
TRoV-2          GAA UUC GAG ACA GAA GCG GUU GUA GAC UCA GAA GAU UGU GGG UGU GCU UAC GAG UAU CCC
                *** *** **  *** *** **  *** *** *** **  **  *** *** *** *** *** *** *** *** *

                                                                   Last upstream ORFx-
                                                                   frame stop (TRoV-1)
TRoV-1          UCC GAG ACC GAG AGU GAA GCU UGC CUU UGC CCC GGA UAC GCG AUA GAA GGA AUC UGC GAU
TRoV-2          UCU GAG ACC GAA AGC GAA GGU UGC CUU UGU UAC GGA UAC AGA AUC GAG AGA GUC UGC GAU
                **  *** *** **  **  *** * * *** *** **    * *** ***     **  **   **  ** *** ***

TRoV-1          UGC GAU UGG UAC GAA GAC AGA CCC GAA ACC AGC GAC AGC UCU GAG CUU UUC ACC CAG UGG
TRoV-2          UGC GAC GUG CGC GAA GUC AGA UCC GAA ACU AAC GAC AGC UCG GAG CUU UUC ACC GAU UGG
                *** **    *   * *** * * ***  ** *** **  * * *** *** **  *** *** *** ***  *  ***

                                                ORF1 stop
```

```
TRoV-1          GAA AGG CUC GAA CUC UUU UCU GAC UAA -AA UCC ACA AGA CAU UUA CUU UCU UAG UAA UAG
TRoV-2          GAA AGG UUC CAA CUC UUU UCU GAC UAA -AA UUC ACA AGA CAU UCA CUU UAC UAU CAA UAG
                *** ***  **  ** *** *** *** *** ***  ** * * *** *** *** * * *** *   **   ** ***

                                            ORF2a (AUG2 or 3; strong context)
TRoV-1          GUA AGU UUA AAC UGU GGA AUC AUG UUG UCA UUA AGG AGU AUA GUG AAG CUG AUC GUA GCU
TRoV-2          GAA AGU UUA GAU UGU GGA AUA AUG UUG UCA UUA AGG AGU AUA GUG AAG CUG AUC GUA GCU
                * * *** ***  *  *** *** **  *** *** *** *** *** *** *** *** *** *** *** *** ***

TRoV-1          GCG UUG AAC GUA AUG UUU GUC GUG ACG AUA GGA GUG UGU GCU CGA GUG UUA GCC CCA GAG
TRoV-2          GUG UUG AAC AUA AUG UUU GUC GCG ACG AUA GGA GUU UGU ACU CGG AUG UUA GCC CCA GAG
                * * *** ***  ** *** *** *** * * *** *** *** **  ***  ** **   ** *** *** *** ***

TRoV-1          AGG CCA GUG AAC UGG AAC UUU GUG GCG CUA CUG CUG ACC CCA GUG CUA GCA UUG AUA GCA
TRoV-2          AUG CCA AUC AAU UGG AAC UUA GUG GCU UUG UUG CUA GCC CCA GUG CUA GUA UUG ACA GCA
                * * *** *   **  *** *** **  *** **   *   ** **   ** *** *** *** * * *** * * ***

TRoV-1          UUC GAG CUG CUU ACC GAG CUA AGA AAA UGG AUG GUG UAC ACC GUA AAG GAA GAA GAC CUU
TRoV-2          UCC GAG CUG CUU ACC GAG CUA AGA AAG AGG AUG GUG CAC ACC GUA AAG GAA GAA GAC CUA
                * * *** *** *** *** *** *** *** **   ** *** ***  ** *** *** *** *** *** *** **

                              ORFx stop
TRoV-1          CCA GCA CCU CUU AGC UUG GAU UCA ACU CCG AGG UUC GAC CCC AUU CAC GGA AUA ACG UCA
TRoV-2          CCC GUG CCU CUU AGC UUG GAU UCA ACU CCG AGG UUC GAC CCC AUC UAU GGA AUA ACA UCA
                **  *   *** *** *** *** *** *** *** *** *** *** *** *** **   *  *** *** **  ***
```

Ryegrass mottle virus

```
                5'UTR
DQ481606        --- --- --- --- --- --- --- --- --- --- --- --- --- --- --- --- --- --- --- ---
EF091714        ACA AAU AGA GUU AUU AAA UUA ACU CUA UUG AAC CCG UUA UCC GGU GAU AUA GGA CAU CUG
AB040446        ACA AAU AGA GUU AUU AAA UUA ACU CUA UUG AAC CCG UUA UCC GGU GAU AUA GGA CAU CUG


                                                    ORF1 (AUG1; weak context)
DQ481606        --- --- --- --- --- --- --- --- --- --- --- --- --- --- --- --- --- --- --- ---
EF091714        UGA ACU AAC AUC ACA AUA AUC AGU UAG CCC CCU CGA UUU AUG CCU UCA GUG GUU AUC GAG
AB040446        UGA ACU AAC AUC ACA AUA AUC AGU UAG CCC CCU CGA UUU AUG CCU UCA GUG GUU AUC GAG


DQ481606        --- --- --- --- --- --- --- --- --- --- --- --- --- --- --- --- --- --- --- ---
EF091714        GUU UGC UCA UAC GAC GAG GAG ACA GGA GAU UGU GAA CUU GAG AGU ACG UCG AAG AUA UUC
AB040446        GUU UGC UCA UAC GAC GAG GAG ACA GGA GAU UGU GAA CUU GAG AGU ACG UCG AAG AUA UUC


DQ481606        --- --- --- --- --- --- --- --- --- --- --- --- --- --- --- --- --- --- --- ---
EF091714        ACC AGU AAC UUC GAC GGG ACU UAC GUC CUA UAU ACU CAC AGU UCC GGG CCG AAA UAC GCC
AB040446        ACC AGU AAC UUC GAC GGG ACU UAC GUC CUA UAU ACU CAC AGU UCC GGG CCG AAA UAC GCC


DQ481606        --- --- --- --- --- --- --- --- --- --- --- --- --- --- --- --- --- --- --- ---
EF091714        GGA UCA ACU GUC ACA CUC GUC UGC CCU CAC UGC GGG GUG AGU GAA CAA GCG ACG UUC CCG
AB040446        GGA UCA ACU GUC ACA CUC GUC UGC CCU CAC UGC GGG GUG AGU GAA CAA GCG ACG UUC CCG


DQ481606        --- --- --- --- --- --- --- --- --- --- --- --- --- --- --- --- --- --- --- ---
EF091714        ACC AGA GGC CUU UCA AGC AAC UGG GGU CAC CAC GGG CCG CAC GAC UUA CGA CUU GAU UGU
AB040446        ACC AGA GGC CUU UCA AGC AAC UGG GGU CAC CAC GGG CCG CAC GAC UUA CGA CUU GAU UGU


DQ481606        --- --- --- --- --- --- --- --- --- --- --- --- --- --- --- --- --- --- --- ---
EF091714        AAG GCG CGG CAC UGG CAC GGA ACG UGC GAG GUC AUU CCC UCC UCU GAG CAA GAG UCG CGU
AB040446        AAG GCG CGG CAC UGG CAC GGA ACG UGC GAG GUC AUU CCC UCC UCU GAG CAA GAG UCG CGU


DQ481606        --- --- --- --- --- --- --- --- --- --- --- --- --- --- --- --- --- --- --- ---
EF091714        GUA GCG UCU GAC GGG GUU UUC GAA CCA GGU CUA ACG CAC AUC ACC ACG ACU CGA GGG CAG
AB040446        GUA GCG UCU GAC GGG GUU UUC GAA CCA GGU CUA ACG CAC AUC ACC ACG ACU CGA GGG CAG


                 Last upstream                                        Likely ORFx
               ORFx-frame stop          ORF1 stop                     start sites
```

```
DQ481606        --- --- --- --- --- --- --- --- --- --- --- --- --- --- --- --- --- --- --- ---
EF091714        UCU UGG ACU AGA CAU CAU UAG UCC CUC CCA GUU CUC GCU GGG AGU CCC GCA GCC UGG CUA
AB040446        UCU UGG ACU AGA CAU CAU UAG UCC CUC CCA GUU CUC GCU GGG AGU CCC GCA GCC UGG CUA


DQ481606        --- --- --- --- --- --- --- --- --- --- --- --- --- --- --- --- --- --- --- -GU
EF091714        CGG GAA AUA UCG GUG GGG UUG AUC AGA CCU ACA CCU CUU CGA GAA AAG CGC UCU UCG AGU
AB040446        CGG GAA AUA UCG GUG GGG UUG AUC AGA CCU ACA CCU CUU CGA GAA AAG CGC UCU UCG AGU
                                                                                             **

                                                                        ORF2a (AUG2; strong context)
DQ481606        ACU CCC UCG GCA GAC UAU UCU GUU GCG UCU CCC AUA GUG AAG AUG UAC GGG UCA AUA GUA
EF091714        ACU CCC UCG GCA GAC UAU UCU GUU GCG UCU CCC AUA GUG AAG AUG UAC GGG UCA AUA GUA
AB040446        ACU CCC UCG GCA GAC UAU UCU GUU GCG UCU CCC AUA GUG AAG AUG UAC GGG UCA AUA GUA
                *** *** *** *** *** *** *** *** *** *** *** *** *** *** *** *** *** *** *** ***

DQ481606        AGC AUG GUG GUU CAG GCG GCG CUC GCU UUC CUA CUG UCC AAG GAC CCA GAG UCC AGG ACU
EF091714        AGC AUG GUG GUU CAG GCG GCG CUC GCU UUC CUA CUG UCC AAG GAC CCA GAG UCC AGG ACU
AB040446        AGC AUG GUG GUU CAG GCG GCG CUC GCU UUC CUA CUG UCC AAG GAC CCA GAG UCC AGG ACU
                *** *** *** *** *** *** *** *** *** *** *** *** *** *** *** *** *** *** *** ***

DQ481606        GCG GUG AGA GGG CUG CUA GCA UCA CUA ACG GGG ACG GCU CUG AUC CUG GGG UGG CAA CUG
EF091714        GCG GUG AGA GGG CUG CUA GCA UCA CUA ACG GGG ACG GCU CUG AUC CUG GGG UGG CAA CUG
AB040446        GCG GUG AGA GGG CUG CUA GCA UCA CUA ACG GGG ACG GCU CUG AUC CUG GGG UGG CAA CUG
                *** *** *** *** *** *** *** *** *** *** *** *** *** *** *** *** *** *** *** ***

DQ481606        CUG UGC GUC UUG CGC GCU UGG CUA GCA ACC UCU CUG GAA AAG GAA CCG GAA GCU CCG GAA
EF091714        CUG UGC GUC UUG CGC GCU UGG CUA GCA ACC UCU CUG GAA AAG GAA CCG GAA GCU CCG GAA
AB040446        CUG UGC GUC UUG CGC GCU UGG CUA GCA ACC UCU CUG GAA AAG GAA CCG GAA GCU CCG AAA
                *** *** *** *** *** *** *** *** *** *** *** *** *** *** *** *** *** *** ***  **

                              ORFx stop
DQ481606        GAC UCC UGG AUU GAU CUG UGU GGU CCG CCA CGC UUU GAU CCA UCG AAA GGA GUU UAU GGA
EF091714        GAC UCC UGG AUU GAU CUG UGU GGU CCG CCA CGC UUU GAU CCA UCG AAA GGA GUU UAU GGA
AB040446        GAC UCC UGG AUU GAU CUG UGU GGU CCG CCA CGC UUU GAU CCA UCG AAA GGA GUU UAU GGA
                *** *** *** *** *** *** *** *** *** *** *** *** *** *** *** *** *** *** *** ***
```

Velvet tobacco mottle virus

```
                                                                    ORF1 (AUG1; medium/weak
                  5'UTR                                                  context in dicots)
HM754263        --A CAA AAU AUA UUG AAG GGA AAG UCU UGC ACU CAG UAC CCA UUA UAU UUU GAU AUG CCC
HQ680399        --- --- --- AUA UUG AAG GGA AAG UCU UGC ACU CAG UAC CCA UUA UAU UUU GAU AUG CCC
                            *** *** *** *** *** *** *** *** *** *** *** *** *** *** *** *** ***


                         sORF (AUG2; weak context)
HM754263        AGC AUU GAU GUU GAA GUA GAA AAG AUC CUG CAC UUG AGC AAC AGA AAG AGA CUA AGG UCU
HQ680399        AGC AUU GAU GUU GAA GUA GAA AAG AUC CUG CAC UUG AGC AAC AGA AAG AGA CUA AGG UCU
                *** *** *** *** *** *** *** *** *** *** *** *** *** *** *** *** *** *** *** ***

HM754263        GUC UGU GUG GCG AGG AAG AAG ACG UUC GUA GAC AAG AUA UCC GAA GGA UUU GUC UGC ACU
HQ680399        GUC UGU GUG GCG AGG AAG AAG ACG UUC GUA GAC AAG AUA UCC GAA GGA UUU GUC UGC ACU
                *** *** *** *** *** *** *** *** *** *** *** *** *** *** *** *** *** *** *** ***

HM754263        CUU UAC GGA GAG UAC GAU CAC GAC UAC GUC ACC AGU GUG CAU UUG CAC AUU GUU UGC UCG
HQ680399        CUU UAC GGA GAG UAC GAU CAC GAC UAC GUC ACC AGU GUG CAU UUG CAC AUU GUU UGC UCG
                *** *** *** *** *** *** *** *** *** *** *** *** *** *** *** *** *** *** *** ***

HM754263        UGC GGU AGA GCU UUC UUU GAU UUU GUU GAG UUC AAG GAC AUA AAA CUC CGA GAU UUC CAA
HQ680399        UGC GGU AGA GCU UUC UUU GAU UUU GUU GAG UUC AAG GAC AUA AAA CUC CGA GAU UUC CAA
                *** *** *** *** *** *** *** *** *** *** *** *** *** *** *** *** *** *** *** ***

HM754263        UAU CAA AGU CAG UGU AUC UGU CCC ACC AGG AAU UGG UCG ACG GUC GCU UAC ACU GAU UUU
HQ680399        UAU CAA AGU CAG UGU AUC UGU CCC ACC AGG AAU UGG UCG ACG GUC GCU UAC ACU GAU UUU
                *** *** *** *** *** *** *** *** *** *** *** *** *** *** *** *** *** *** *** ***

HM754263        ACU ACC GUC AAC UGC CAA GUG GAA GGU UGU GAU UAC UGU GAG GGG AUU GAG UCA GAC UCU
HQ680399        ACU ACC GUC AAC UGC CAA GUG GAA GGU UGU GAU UAC UGU GAG GGG AUU GAG UCA GAC UCU
                *** *** *** *** *** *** *** *** *** *** *** *** *** *** *** *** *** *** *** ***

                                             Last upstream
                                             ORFx-frame stop
```

```
HM754263         GAU UCG GAU UCU GAG GCC AUC AUA GAG GAA UUU CUU CAA AAG UUU UCC GAG GUU GGC AUC
HQ680399         GAU UCG GAU UCU GAG GCC AUC AUA GAG GAA UUU CUU CAA AAG UUU UCC GAG GUU GGC AUC
                 *** *** *** *** *** *** *** *** *** *** *** *** *** *** *** *** *** *** *** ***

                                                         ORF1 stop
HM754263         UCU GGA UCG UCC AGU UCU CCC CAG ACU AAC UAG -GA CUC GGU UCC GUU UAU UUU CUU ACA
HQ680399         UCU GGA UCG UCC AGU UCU CCC CAG ACU AAC UAG -GA CUC GGU UCC GUU UAU UUU CUU ACA
                 *** *** *** *** *** *** *** *** *** *** ***  ** *** *** *** *** *** *** *** ***

                                                         ORF2a (AUG3; strong context)
HM754263         AAU CAU UUA AUU GUU UUG UAA UUG AGA AAG AUG UUG AGC GAG UUA GUC CAG UUG UGU CUU
HQ680399         AAU CAU UUA AUU GUU UUG UAA UUG AGA AAG AUG UUG AGC GAG UUA GUC CAG UUG UGU CUU
                 *** *** *** *** *** *** *** *** *** *** *** *** *** *** *** *** *** *** *** ***

HM754263         UCA GCA ACC AUG ACU CUA GCG CUG GUA GUG UCA AUG AUG CUG GAC CCG AGC UAC GGG UGG
HQ680399         UCA GCA ACC AUG ACU CUA GCG CUG GUA GUG UCA AUG AUG CUG GAC CCG AGC UAC GGG UGG
                 *** *** *** *** *** *** *** *** *** *** *** *** *** *** *** *** *** *** *** ***

HM754263         CUG AUC ACC AUG UCU GUG AUG UUA GUC UCC UUA GAG CUG ACA AUC AGG CCG UUC AGG CGC
HQ680399         CUG AUC ACC AUG UCU GUG AUG UUA GUC UCC UUA GAG CUG ACA AUC AGG CCG UUC AGG CGC
                 *** *** *** *** *** *** *** *** *** *** *** *** *** *** *** *** *** *** *** ***

                                                           ORFx stop
HM754263         UCU CUA GAC UAC AUG AAA AUU GUG GUC CGA GAU GAG CCC AAU GAG CCU GAA GUU GCU AGG
HQ680399         UCU CUA GAC UAC AUG AAA AUU GUG GUC CGA GAU GAG CCC AAU GAG CCU GAA GUU GCU AGG
                 *** *** *** *** *** *** *** *** *** *** *** *** *** *** *** *** *** *** *** ***
```

***Imperata* yellow mottle virus** AM990928

```
-GA UUU UGA GCC UAC UUC CAC CUA UAA CAC CCU ACC UGG CCU UGU AUG ACC CGU ACU GAG  ORF1 (AUG1)
AUC ACU CUC CGG GCC ACA UCA AGC ACC AGG AAG GCC GCU CGA CGU CAU AGU AGG GAC GGG
ACC AGG AAG GCC GCU CGA CGU CAU AGU AGG GAC GGG ACA CUC GUC UGG GCU GUG CAU UCG
CAC GAG AUC GAC GAC CCC GUC AUU ACU GCG CCU UAC GUC CCC GGC AUC UUC AGC CAG UUG
ACU CUG GUU GUG UUG UGC CAU ACU CCG GGU UGC CAU UCG AUC AUU GAG UCU ACC UCA GUG
CAG AUC GAG CAU UUC CGG UUG UUG UCC GAU AUC UAU AGG GCG AGA GCC UGC GAG UCG UAC
UCC AUU UGU GAG UCC UGC AGG CAG AAC UCU CCU AGA GUU CGC UCA CCC CGU GCU CCC UAU
UCC UCU UUG AGU GAA GGG AAC ACU AGA CAA GAU UCG GGC UCA GUG GAG AAG UGG GUG CGC
GAC ACC GAG UAU CUC GAG UCA CGA GUU UGC GGU GGU GGU AAC CCU UAU CAU UAC UGC UCU
CAU UGC UAC CCU CCG AAU ACU UCU GUC UAU UCA AGA GUG GUC AAU AAC UCU GAC UCU CGG
CCA UCG GAC AUA GAC GAG ACG ACU GGU GAG GAG UUC UAU CCC CGG UUU UCC AGA CUA UCU
GUC CAG GAU AGG UCG GAC UCG GAC UGA -GC GGU CGG GCU UUC GCG UCA CGC GAC AUG GCC  ORFx start/ORF1 stop
UCU UUU GUU GCU GGU CAG CUA CGG AAG GGG AUG CGG AUG AUG CAC CUU CCA GGU CAC AUC  ORF2a (AUG2)
UUC CUC CUU CUA GGC AUU UGG CUA GCG GGG UUA UGU UCG GAU CAA CUC CCC GUG ACG UCA
UGG UGG UGG GCG AUC CCG UUG ACU AUC AUA GUA AAU UGG AUC GCA UCC CAA GCA GCC GAG
GAU UUC AAU CGG CUG UGC GCA GGG UUG AAG CCC GAG CCA CUA GCU AAA CGC AAG UUU GGC  ORFx stop
```

***Artemisia* virus A** JN620802

```
-CA AAA UUA UUU AAG AGG UUU UAU GUC UAU CGC UUA UAA AGC UGU CAG UUU UAC UCU UAA  sORF (AUG1)
UAA AUU AAC AUC AUU UAC UGU AUG UCU UCA GUG AUU UUC GCG UUU GGC GUG AGC AAA ACU  ORF1 (AUG2)/sORF stop
UAU CCA ACU AUU GUU GAG AAG UAC UGU GGC AGU AGC AAG AAG GUU UUC UUC CGU GGA UUG
GCU CCU GGA AUU GAG ACA UCU UUG ACU GUU GAU UCU CCA AUA AAG GUG AAA GUU GCG AAG
UGG AAC AGG GAU UCA GUG GAC GAA CUA GAG AUA ACC UGC AAG UGU GAA ACU UGU GGU GCG
AAA GCU UUU GAG AUC UUA GAG UUC AAG UAC GCU GAU AAC GGA GAC GAA AUC UGG GAA ACA
GAG GUC GUC GCU GAC UGG UGU UCA UAC UGU GAG ACC AAG AAA UUG UUU GAC UCC GGU GAU
ACU GAA CAC UAU ACU CCC UGU CCU AGC CCG CCU GCU CCG AUU UCA AAC UGA AAG ACC GUG  ORFx start/ORF1 stop
CGA UAU CCA GUA GAC GGG UUU CUC UUU UCG AGA AAU AUC GUA ACC AUU GGU UUU GUU GCA
CUU AUC ACA AUG AAG UUA UCC AAU UUG AUG ACU UUG ACG AUG UUG GCC GGA CUG GUU UCA  ORF2a (AUG3)
GUC AUG AUC GAC AUG UAC CAG AGU CCU CAG UGG AGC CUA GCC UAC UGG CUA AGC CUA AGC
CUA UGG AUA GCA GCA CUU AUG CUG CAA UUC GCC UGG CUA AGC UGG CCG AGA GUG UGU CUC
GAA CCA GAA CCG GUG AAA ACU GAG UUG UGG GGC GAG AUG CUC GGU GAU CCU GAG UUC GAC  ORFx stop
```

Papaya lethal yellowing virus JX123318

```
--A CAA AGU UGG UUU UGU AAG AAA UUG UAU UUU CCA ACU UGU UAA ACC UAC AUG UCU GUG  ORF1 (AUG1)
GUG AUU GAA GUC UAU GAC GAA CAA UCA GUA AGC AAA GUU UUG AUC GAA AGU UUC AAG CUU  sORF (AUG2)
UGG CCU AAG AAA UUA GGU GAU UGU GUC AUC CAG UUG AAA GGU AAG UAC GAC UCU UCC GCA
GUU UGC AGA GGU GAA AUA ACC CUU UGU UGU GAG AAG UGU AGG CGU CAA GAG AUA AUC UCU
```

UUU GAA AAG AAG AGA AUC CUG AUA CAU UGU GAU UGG GAG AAU AAG CUC UGU GAG GAC CCU
UGU AUA GAC CGA UUU GUG AAA CCU GAC GAA GGU UUC UGC AAC UGG UGC GUG GAA GAU CAU
CAG UGC ACC UCC GAG GGU UGU UCA GAU CCC GAC UGU GUG UUU CAC UCA GAU UCC UUG GAA
UUU GGG UGG UCU GAA UCA CUG AAU AAG UGG AUA GAC GCA CAU AAA CAC GGG UUC GUG UUU ORFx start
GAC CGU UAC GAG GCC GAG UGG UAC CAC CCG AUU ACA GCC GAA AUA UUG CUG UCU AGG AGU
CCA GGU CUC UUC AGA GAU UGA -AG AUG ACG UUU AUA GAU UUC CUG CUA GUG UGU CUA GUA ORF1 stop/ORF2a (AUG3)
UGG AAU GUG GGG GCU UUA CUG GUA ACU CAA AAU ACC AAC UGG CUU CCU ACG UGG ACU GUA
CCC CUU GCU GUG AUA AUC CAG CUG GCU UUG AUC UGC AUU UGU AUG CUG UUG CUG AGC GUG
CUG UAC GUU UGG CGA UGG UUG ACU UUG AGG UCA AUA AAA GUA GAU AGU GCA AAC UAU CAC ORFx stop

Lucerne transient streak virus JQ782213

-AC AAA UAA UGU GAA GAA AGA ACU GAA GUU UAA GCA AUU UGU GUA UUC GUA CUU UUG UGC sORF (AUG1)
UUA AAC UUC CAA UCC UGU AUG CCA AGC GUA AUA GUU GAG UGU UAC ACA ACC GAC UCC CAU ORF1 (AUG2)
CGA GAC AUC CCC UUG UUC UCC CAG AAG AUU UGG UUC GAC AAC GUU GAC GAA UUA ACG UCC
GAC UAC GUC CGA GUC UAC AGG GCA GUU AGA GAC UAC UCC AAG GUU GAC UAC AUC GAG GUU
GAG UUG CGC UGU AAC UUU UGU CAC UAU UAC AAC AGU AGG GGG CAG AUC GUA CAA CGA UAU
CUU GGU UGU AAA GAC GUG GAG AUU AGA AAC AUC UCC UUA CCU GAC AAC GAC CCA UAC ACC
UAC ACG GUA UAU UCA GUU GAU UGC GCG AUU UGC CGG AAG AUU CCA ACG GAG UCU GAG GAC
GAA UCG GAC AGU UCU UUC UAA CCC UGG AAA CCC GAA CUA AGC GUA GAA UUA CAC GCU CUC ORF1 stop/ORFx start
GCA GUU UCA UUA ACU AUA AGU UGU UUA ACU GUU UCA CAA GUA UUA CAG UAC AAG AUG ACU ORF2a (AUG3)
UUG AAA CUC UGG GAA GCU CUC AAG GUU CUU GUG GUA GUG UCG AGC AGC UCG AUA GCU GCG
AGU CUA CUA CUU ACA UCC CAG GUG GCA GCA GGA GGU UUA GAC CCG AUG UUG CAA UCC CCA
UUG GCU CUG UGC GUC AUA UCG AUG CUG GUG CUG AUC GUG CGA UAC GCC UGG CAG AAC AUG
CUG CUA AAC UCA GUA GAG GUU AGG GAU GAA ACU CCC CCC GGC AUG UUC AUC AAU CUU GUA ORFx stop

Predicted Px peptide sequences for representative sobemovirus sequences.

Note that the ORFx initiation site is not always clear from sequence analysis. Moreover, multiple initiation sites may be utilized in some species. Where there is ambiguity, one possible initiation site is presented for each species. Since the initiation site is not definitively known, N-terminal amino acids are presented using the standard genetic code translation, even though non-AUG initiators are expected to be decoded as methionine by initiator-Met-tRNA.

>L20893 rice yellow mottle virus (**G**UC CUG C)
LPTSKERSWFRLTRDGLFCCWSLSHSHQGNDEGGLPPSHRLGWVDSGRDSLRAPTRDGVIVGNPLSYHSKLDRAVSSREL
QPVR*

>AM990928 *Imperata* yellow mottle virus (**A**GG AUA **G**)
IGRTRTERSGFRVTRHGLFCCWSATEGDADDAPSRSHLPPSRHLASGVMFGSTPRDVMVVGDPVDYHSKLDRIPSSRGFQ
SAVRRVEARATS*

>DQ680848 cocksfoot mottle virus (**G**GA AUU **G**)
IAATAVAGQSSTEVSWLRVTKNGLFCCWKLQVRDVDEQDELVKASSPHIRRNGGSHDRLPTHSDMYGDPSERGAQLDRLR
SMRRSFRANSGRFLGNDSS*

>EF091714 ryegrass mottle virus (**A**GC CUG **G**)
LATGNIGGVDQTYTSSRKALFEYSLGRLFCCVSHSEDVRVNSKHGGSGGARFPTVQGPRVQDCGERAASITNGDGSDPGV
ATAVRLARLASNLSGKGTGSSGRLLD*

>JN620802 *Artemisia* virus A (**A**AA CUG **A**)
LKDRAISSRRVSLFEKYRNHWFCCTYHNEVIQFDDFDDVGRTGFSHDRHVPESSVEPSLLAKPKPMDSSTYAAIRLAKLA
ESVSRTRTGEN*

>JQ782213 lucerne transient streak virus (**A**CC CUG **G**)
LETRTKRRITRSRSFINYKLFNCFTSITVQDDFETLGSSQGSCGSVEQLDSCESTTYIPGGSRRFRPDVAIPIGSVRHID
AGADRAIRLAEHAAKLSRG

>AF208001 subterranean clover mottle virus (**A**CA GUG **G**)
VAQKATGRNLQTTKTTHELHIVPATTKVTSYGCFNYKAFSLFKKETIKNEYRHYSSNLPILDEPSFEHGGGGPTGNKGGL
IKPPVVSYDDSHLAARTERAVRLARLAKMEGCDSEGGEPARGDKLSGGAFP*

>HM163159 sowbane mosaic (**A**GC AUU **G**)
IGRQDPVCGCGICTKVHSKTTLSTTTSRLKWFARSVTRCFRRLQHRRVKSPVVGQCSLTELTEELSRLRAERKPKSYKVN
PTVTSSDFRPPVKNASFTVWLPPTAHQPPSWSSDLRLRARGLPSRTQLSDDGSDDSAHYRNVGDSLSKPILDRATALSLS
RREDLTAEAIAAHWRSLLGSL*

>AM940437 *Rubus* chlorotic mottle viruses (**G**AC AUU **G**)

IGRQDPACVCGICTKANSRTARSTTTSLLKCVARSVTRCFRLPPHRRARSPVVGQYSLTESTEVLSRLRAERKLASYKVN
PTVTSSVSSLPAQNASFTVWLPPAAHQPPSWSSDLRLHKRGLPSRTQQFDDGSDDSTHYRNVVGSLAKPILDRAAALSLS
RREDLTAEAIAAHWRSLFGSL*

>M23021 southern cowpea mosaic virus (**A**CA AUU **U**)
ISILLKFVRCLVKPLLVCGGNPSSQSTTTGTFTSRTTPSLTREYNASSTPAENVSSELPFICPPSCEHVELRRVRKCVHL
RVIRPQPQYSNCGLDDPVRYRALVEHVRGVLRDSLREGASFA*

>JF495127 soybean yellow common mosaic virus (**A**CC CUG **U**)
LYPCGYPSTAEFVKLRLTLSSTRSWCLGIVKTASSCFVTPKGDLCESEHEEAFSLADFTTRKKRPFVSHASSRAVCVHAL
GREHGKLCYTPRFVHAGSIQVRSHGSSCGLDDPVRYRALVESVTRELPLRICPSSASSRS*

>AY004291 *Sesbania* mosaic virus (**A**GG CUG **G**)
LVEFRGSTLLVKTGKCFSGLCGAQQERNTSIRVERKFAENVSSELQGLCPPPSQYGKLRDPCGHVPYGGAQPLTRCPGGG
IDDFVRYSALVERLIRDLPAQVHVGEVVS*

>DQ875594 southern bean mosaic virus (**A**GA ACG **G**)
TGICLGLERSGRRERNTSTSVRNLSADNVSSRPLTIIPDNVSKCYLRGNLVRHPYGGVPTRITSSNSGLDDPVRYAALVE
RVIRDIPL*

>HM754263 velvet tobacco mottle virus (**A**GG UUG **G**)
LASLDRPVLPRLTRTRFRLFSYKSFNCFVIEKDVERVSPVVSFSNHDSSAGSVNDAGPELRVADHHVCDVSLLRADNQAV
QALSRLHENCGPR*

>JX123318 papaya lethal yellowing virus (**A**AC ACG **G**)
TGSCLTVTRPSGTTRLQPKYCCLGVQVSSEIEDDVYRFPASVSSMECGGFTGNSKYQLASYVDCTPCCDNPAGFDLHLYA
VAERAVRLAMVDFEVNKSR*

>TRoV-1 turnip rosette virus (**G**CG AUU **G**)
IAIGTKTDPKPATALSFSPSGKGSNSFLTKIHKTFTFLVIGKFKLWNHVVIKEYSEADRSCVERNVCRDDRSVCSSVSPR
EASELELCGATADPSASIDSIRAAYRAKKMDGVHRKGRRPSSTS*

T-COFFEE[#] alignments of Px for selected inter-species clades.

[#] Notredame C, Higgins DG, Heringa J (2000) T-Coffee: A novel method for fast and accurate multiple sequence alignment. *J Mol Biol* **302**:205-217.

Rice yellow mottle virus (L20893), *Imperata* yellow mottle virus (AM990928), and cocksfoot mottle virus (DQ680848).

```
L20893          L-------PTSKERSWFRLTRDGLFCCWSLSHSHQGNDE---GGLP--PS
AM990928        I------GRTRTERSGFRVTRHGLFCCWSATEGDA-DDAPSRSHLP--PS
DQ680848        IAATAVAGQSSTEVSWLRVTKNGLFCCWKLQVRDV-DEQ---DELVKASS
                :        : .* * :*:*:.******.    .  ::    . *   .*

L20893          HRLGWVDSGRDSLRAPTRDGVIVGNPLSYHSKLDRAVSSR-EL-------
AM990928        RH---LASGVMFGSTPR-DVMVVGDPVDYHSKLDRIPSSR-GFQSAVRRV
DQ680848        PH--IRRNGGSHDRLPT-HSDMYGDPSERGAQLDRLRSMRRSFRANSGRF
                 :     .*      *  .  : *:* .  ::***  * *  :

L20893          --QPVR
AM990928        EARATS
DQ680848        LGNDSS
                  .
```

Ryegrass mottle virus (EF091714), and *Artemisia* virus A (JN620802).

```
EF091714        LATGNIGGVDQTYTSSRKALFEYSLGRLFCCVSHSEDVRVNSKHGGSGGA
JN620802        LK-------DRAISSRRVSLFEKYRNHWFCCTYHNEVIQFDDFD-DVGRT
                *        *:: :* * :***   .: ***. *.* ::.:. . . * :

EF091714        RFPTVQGPRVQDCGERAASITNGDGSDPGVATAVRLARLASNLSGKGTGS
JN620802        GFS--HDRHVPESSVEPSLLAKPKPMDSSTYAAIRLAKLAESVSRTRTGE
                 *.  :. :* :.. ..: ::: .  *... :*:***:**..:* . **.

EF091714        SGRLLD
JN620802        -----N
                     :
```

Lucerne transient streak virus (JQ782213), and subterranean clover mottle virus (AF208001).

```
JQ782213        LETRTK-RRI-----------------TRSRSFINYKLFNCFTSITVQDD
AF208001        VAQKATGRNLQTTKTTHELHIVPATTKVTSYGCFNYKAFSLFKKETIKNE
                :  ::. *.:                 . * . :*** *. *.. *::::

JQ782213        FETLGSSQGSCGSVEQLDSCESTTYIPGGSRR--FRPDVAIPIGSVRHID
AF208001        YRHYSSNLP----ILDEPSFEHGGGGPTGNKGGLIKPPVVS--YDDSHLA
                :.  .*.      : :  * *     * *.:   ::* *.    .  *:

JQ782213        AGADRAIRLAEHA---------------AKLS---RG
AF208001        ARTERAVRLARLAKMEGCDSEGGEPARGDKLSGGAFP
                * ::**:***. *                ***
```

Southern cowpea mosaic virus (M23021), soybean yellow common mosaic virus (JF495127), *Sesbania* mosaic virus (AY004291), and southern bean mosaic virus (DQ875594).

```
M23021          I----------------------SILLKF----VRCLVKPL-LVCGGNPS
JF495127        LYPCGYPSTAEFVKLRLTLSSTRSWCLGIVKTASSCFVTPKGDLCES-EH
AY004291        LV--------EFRGS--------TLLVKT----GKCFSG----LCGA-QQ
DQ875594        ----------------------------T----GICLGL----ERSG-RR
                                                   *:         .

M23021          SQSTTTGTFTSRTTPSLTREYNASSTPAENVSSELPFICPPSCEHVELRR
JF495127        EEAFSLADFTTR----------KKRPFVSHASSRAVCVHALGREHGKLCY
AY004291        ERN-----TSIR----------VERKFAENVSSELQGLCPPPSQYGKLRD
DQ875594        ERN-----TSTS----------VRNLSADNVSSRPLTIIPDNVSKCYLRG
                ..       :                 ..:.**.   : .   .   *

M23021          VRKCVHLRVIRPQPQYSNCGLDDPVRYRALVEHVRGVLRDSL--REGASF
JF495127        TPRFVHAGSIQVRSHGSSCGLDDPVRYRALVESVTRELPLRICPSSASSR
AY004291        PCGHVPYGGAQPLTRCPGGGIDDFVRYSALVERLIRDLPAQV--HVGEVV
DQ875594        NLVRHPYGGVPTRITSSNSGLDDPVRYAALVERVIRDIP-----------
                                .. *:** *** **** :   :

M23021          A
JF495127        S
AY004291        S
DQ875594        L
```
