# Supplementary material for: An essential fifth coding ORF in the sobemoviruses
Source: Virology. 2013 Nov;446(1-2):397–408. doi: 10.1016/j.virol.2013.05.033 (PMC3791421; doi:10.1016/j.virol.2013.05.033)
Supplement: Supplementary file 2 — Supplementary data [file mmc2.doc]

**Table S1.** Primers used for RT-PCR and sequencing of TRoV RNA

| Strain/ Fragment | PCR primers | sequence | Sequencing primers | sequence |
| --- | --- | --- | --- | --- |
| 1/1 | T7TRoV  TRoV1497L | GCGGTCTAGACGCGTTAATACGACTCACTATAGGCAAAATAAATACAAGAAAGAAAGATTTTCTCC  GAAAGCCCCTAAGACCAACCCGACAT | TRoV374L  T7TRoV2  TRoV665U  TRoVconF1L  TRoV1497L | CACTCTCGGTCTTGGAATGATACTCG |
| 1/2 | TRoVconF2U  TRoVconF2L | CCCGGCAGGAAAGGAACCACCATCTCTCGT  CCTTCGACAGAGTCATCACCCATGGCAATACACCA | TRoV1757L  TRoV1540U  TRoVF2L2  TRoV2475U  TRoVconF2U | CAGTTTAAAGAGCTCACTGGTAGAGTTTCGA  GCTGATGAGGCTGCGGATAGAGA |
| 1/3 | TRoV2475U  TRoVend2 | CGCCAAAACCGGAAAGAAATCACA  ACCACGTGGGGTTCCCAAATGAAT | TRoV3135L  TRoV2475U  TRoVconF3U  TRoV3429U  TRoVend2 | CTGGCCTGATGAAATCTGGATCCTATTGCACATCTTC  TTCCGTGTTGCAAGTCATACC |
| 2/1 | T7TRoV  TRoV1497L  T7TRoV2  TRoVconF1L | ACCACGTGGGGTTCCCAAATGAAT  GCTTAGCCCACACCGTCGGTGGGAC | T7TRoV2  TRoVconF1L  TRoV374L2 | CACTCTCGGTCTCGGAATGATACTCG |
| 2/2 | TRoV665U  TRoV3135L  TRoVconF2U  TRoVconF2L2 | TGAACTGGAACTTTGTGGCGCTACTGC  GGTGGGTCCCTAACTCAGCCTCAAGTAT  CCTTCAACAGAGTCATCTCCCATGGCTATACACCAC | TRoV1757L  TRoV1540U  TRoVF2L2  TRoV2475U |  |
| 2/3 | TRoV2475U  TRoVend2 |  | TRoV3135L  TRoV2475U  TRoV3429U  TRoVend2 |  |

**Table S2.** Primers used for RACE and cloning

| Procedure | Primers | sequence |
| --- | --- | --- |
| 5'RACE | TRoV374L2 (TRoV-1)  TRoV374L3 (TRoV-2)  TRT5 (G tailed cDNA)  T3E (A tailed cDNA)  T-outer (G tailed cDNA)  C-outer (A tailed cDNA)  TRoV241L | CGCTTTCGGTCTCAGAGGGATACTCG  CTGCAGAATTCCCCCCCCCCCCCCCCC  AATTAACCCTCACTAAAGGTTTTTTTTTTTTTTT  GCGGCTGCAGTCTAGAATTAACCCTCACTAAAGGTTTTT  GCAAGGAGCTGCAGAATTCCCCC  GTACACGGTTTTCCCACACTCTTCAC |
| 3' RACE | TRoV3405U  T3E  TRoV3940U  T-outer | CTCAGTTTCGAACACGCCAACTCCTTCC  ATCGCCTCGTCCTTAAACGTATAGAATGG |
| Assembly and cloning | T7TRoV2  TRoVconF2L (TRoV-1)  TRoV3135L (TRoV-2)  TRoVconF3U (TRoV-1)  TRoVconF3Ub (TRoV-2)  TRoVend4 | CTGGTCTGATGAAATCTGGATCCTATTGCACGTCTTC  CGGCGCTGGCCAAAGACAGATAACCGCCTGTCT |

**Table S3.** Mutagenesis primers

| Strain/mutant | primer | sequence |
| --- | --- | --- |
| 1/PTC1  2/PTC1 | TR3PTC1U  TR3PTC1L  TR1PTC1U  TR1PTC1L | AAAGGCTCGAACTCTTTTCTGACTAAAATCTAATAGACATTTACTTTCTTAGTAATAGGTAAG  CTTACCTATTACTAAGAAAGTAAATGTCTATTAGATTTTAGTCAGAAAAGAGTTCGAGCCTTT  GATTGGGAAAGGTTCCAACTCTTTTCTGACTAAAATTTAATAGACATTCACTTTACTATCAA  TTGATAGTAAAGTGAATGTCTATTAAATTTTAGTCAGAAAAGAGTTGGAACCTTTCCCAATC |
| 1/PTC2  2/PTC2 | TR3PTC2U  TR3PTC22  TR1PTC1U  TR1PTC2L | TAAGTTTAAACTGTGGAATCATGTTGTCATTACGTAGTATAGTTAAGCTGATCGTAGCTGC  GCAGCTACGATCAGCTTAACTATACTACGTAATGACAACATGATTCCACAGTTTAAACTTA  GGAAAGTTTAGATTGTGGAATAATGTTGTCATTACGTAGTATAGTTAAGCTGATCGTAGCTGTG  CACAGCTACGATCAGCTTAACTATACTACGTAATGACAACATTATTCCACAATCTAAACTTTCC |
| 1/PTC3  2/PTC3 | TR3PTC3U  TR3PTC3L  TR1PTC1U  TR1PTC1L | CGAGTGTTAGCCCCTGAGAGGCCAGTGAA  TTCACTGGCCTCTCAGGGGCTAACACTCG  ACTCGGATGTTAGCCCCTGAGATGCCAATCAATTG  CAATTGATTGGCATCTCAGGGGCTAACATCCGAGT |
| 1/pol-mut  2/pol-mut | TR3PolmutU  TR3PolmutL  TR1PolmutU  TR1PolmutL | TGGTGTATTGCCATGGCTGATGCCTCTGTCGAAGGTTAC  GTAACCTTCGACAGAGGCATCAGCCATGGCAATACACCA  GTGGTGTATAGCCATGGCAGATGCCTCTGTTGAAGGTTACG  CGTAACCTTCAACAGAGGCATCTGCCATGGCTATACACCAC |

**Table S4. Primers used for insertion of TRoV constructs into pBI121**

| Strain | Primer | sequence |
| --- | --- | --- |
| 1 | TR35SU | GGAAGTTCATTTCATTTGGAGAGGCAAAATAAATACAAGAAAGAAAGATTTTCTCC |
| 1 | TR35SL | GGAGAAAATCTTTCTTTCTTGTATTTATTTTGCCTCTCCAAATGAAATGAACTTCC |
| 1 | 35SU | GATGGTTAGAGAGGCTTACGCAGC |
| 2 | pBI1U | CTTGCATGCCTGCAGGTCC |
| 1 | TRoV3Xend | CTCGCCTCTAGACTAGCTAAGAGGTGCTGGAAGGTC |
| 2 | TRoV1Xend | CTCGCCTCTAGACTAGCTAAGAGGCACGGGTAGGTC |
